# NMR-based investigations of acyl-functionalized piperazines concerning their conformational behavior in solution†

**DOI:** 10.1039/c8ra09152h

**Published:** 2018-12-06

**Authors:** Robert Wodtke, Janine Steinberg, Martin Köckerling, Reik Löser, Constantin Mamat

**Affiliations:** Institut für Radiopharmazeutische Krebsforschung, Helmholtz-Zentrum Dresden-Rossendorf Bautzner Landstraße 400 D-01328 Dresden Germany r.loeser@hzdr.de c.mamat@hzdr.de; Fakultät Chemie und Lebensmittelchemie, Technische Universität Dresden D-01062 Dresden Germany; Institut für Chemie – Anorganische Festkörperchemie, Universität Rostock Albert-Einstein-Straße 4a D-18059 Rostock Germany

## Abstract

Selected *N*-benzoylated piperazine compounds were synthesized to study their conformational behavior using temperature-dependent ^1^H NMR spectroscopy. All investigated piperazines occur as conformers at room temperature resulting from the restricted rotation of the partial amide double bond. In the case of selected mono-*N*-benzoylated and unsymmetrically *N*,*N*′-substituted derivatives, the appearance of the ^1^H NMR spectrum was further shaped by the limited interconversion of the piperazine chair conformations. Therefore, two different coalescence points *T*_C_ were determined and their resulting activation energy barriers Δ*G*^‡^ were calculated to be between 56 and 80 kJ mol^−1^. In most of the cases, *T*_C_ and Δ*G*^‡^ for the amide site appeared to be higher than the corresponding values for the ring inversion. The influences of substituents on rotational and inversion barriers were analyzed by correlation to Hammett constants. The obtained results are discussed and interpreted in the context of literature data. An additional aryl substituent connected at the amine site led to reduced rotational and inversion barriers compared to the free secondary amine. To support and evidence the findings from the NMR analyses, single crystals of selected piperazines were obtained and XRD analyses were performed. To underline the results, two potential TGase 2 inhibitors were investigated showing energy barriers with similar values.

## Abstract 2

The influence of substituents on rotational and inversion barriers in benzoylpiperazines was investigated by dynamic NMR and the observed effects were quantified by correlation analyses using Hammett substituent parameters.

## Graphical abstract

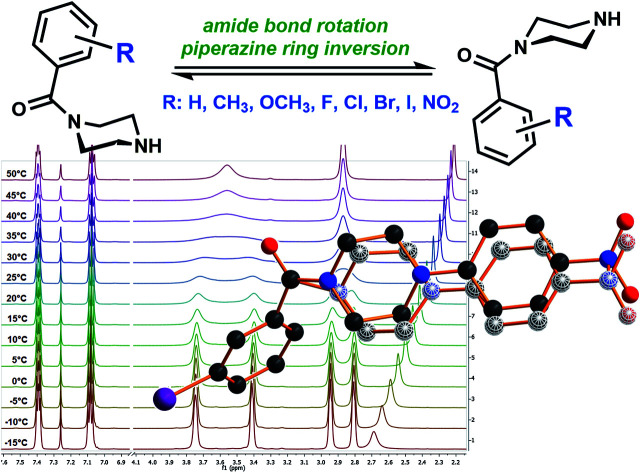

## Introduction

Piperazines belong to the important class of saturated heterocyclic compounds in organic chemistry containing two nitrogen atoms.^1^ The piperazine scaffold has been integrated as a privileged structure and is frequently found in bioactive and pharmacologically relevant compounds across a number of different therapeutic areas. A wide variety of *N*-functionalized piperazines serve as skeletons in pesticides, pharmaceuticals^2–4^ such as analgesics^5^ and other drugs,^6,7^ scaffolds in medicinal chemistry,^8–10^ intermediates in organic reactions,^11,12^ ligands for complexation purposes,^13–15^ for peptide syntheses and modifications^16,17^ as well as building blocks in material sciences *e.g.* for polymerizations.^18^ Furthermore, piperazines are also in use for the synthesis of labeling compounds and building blocks, *e.g.*, for inserting fluorescent dyes^19,20^ or for the introduction of radionuclides^21–23^ into biologically active molecules. The understanding of the conformational behavior of these functionalized piperazines is important not only to explain biological and/or pharmacological effects, but also for applications in material sciences.

Among the different *N*-functionalized piperazines, *N*-acylated piperazines exhibit a complex conformational behavior due to the hindered rotation of the amide bond in addition to the conformational phenomena of the piperazine ring itself. Regarding the first phenomenon, the hindered rotation of the C–N bond in secondary and tertiary amides is well known and has far-reaching implications towards peptide and protein folding.^24^ However, the NMR properties of tertiary benzamides were only rarely studied in the past with *N*,*N*-dimethyl benzamides^25–27^ and *N*,*N*-diethyl benzamides^28^ representing the best characterized compounds. Furthermore, the structural properties and conformational dynamics of tertiary amides including 1-acylpiperazines have been investigated by other spectroscopic methods within several studies.^29,30^ Recently, the NMR behavior of selected benzoylpiperazines, which were used as prosthetic groups for ^18^F-labeling, was investigated.^21^

To shed further light on the conformations of benzoylated piperazines including substituent effects at the benzoyl residue and different moieties at the piperazine ring, novel symmetrically and unsymmetrically *N*,*N*′-functionalized piperazine derivatives were synthesized and characterized in terms of their particular NMR behavior.

## Results and discussion

**Scheme 1:**
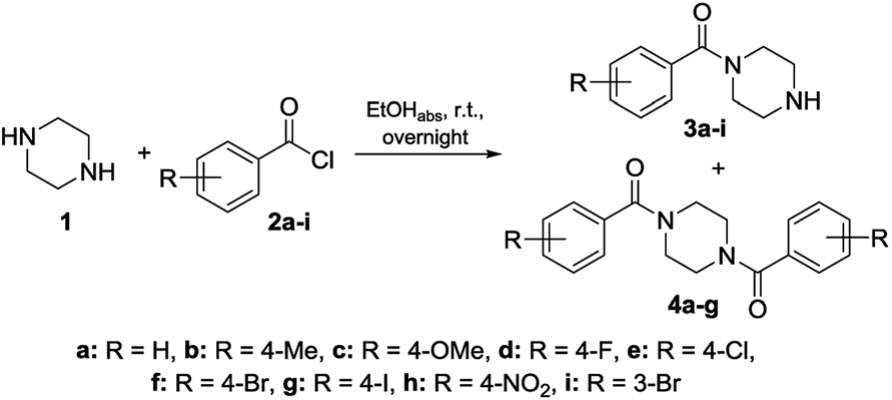
Synthesis of functionalized piperazines 3a–i and 4a–f used for the NMR studies.

**Scheme 2:**
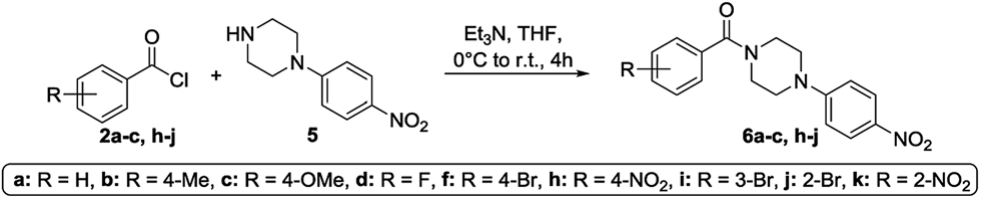
Synthesis of functionalized piperazines 6a–d, f, h–k used in the NMR studies.

To get a deeper understanding on the NMR behavior of acylated piperazines in terms of their conformation at room temperature, three groups of *N*-acylated piperazine derivatives were synthesized. Different substituted benzoyl moieties were used for functionalization to show their influence on the formation of rotamers and the resulting rotation barrier. The first group consists of mono *N*-acylated compounds 3a–i, the second group contains symmetrically *N*,*N*′-diacylated compounds 4a–f and the third group consists of *N*-acylated-*N*′-arylated derivatives 6a–k. The first and second group of benzoylated piperazines was obtained by the reaction of piperazine (1) in excess to the respective benzoyl chlorides 2a–i in a one-pot reaction. Both, the mono-*N*-acylated compounds 3a–i and the *N*,*N*′-diacylated compounds 4a–f were yielded from the reaction mixture.^31^ Most of the compounds are known from the literature and were proven by ^1^H and ^13^C NMR analyses. An overview of the synthesis route to mono-*N*-substituted and symmetrically *N*,*N*-disubstituted derivatives is given in Scheme 1. To determine the influence of an aryl moiety containing the electron withdrawing nitro group at the *N*′-position of the piperazine, a third set of compounds 6a–d, f, h–k was synthesized by using 1.1 equivalents of 1-(4-nitrophenyl)piperazine (5) and 1 equivalent of the respective benzoyl chloride 2a–d, f, h–k in anhydrous THF using triethylamine as base. Compounds 6a–d, f, h–k were obtained in yields ranging from 37 to 98%. Their structural identities were verified by ^1^H and ^13^C NMR analyses. An overview of the synthesis route to these derivatives is given in Scheme 2.

### NMR experiments

**Fig. 1:**
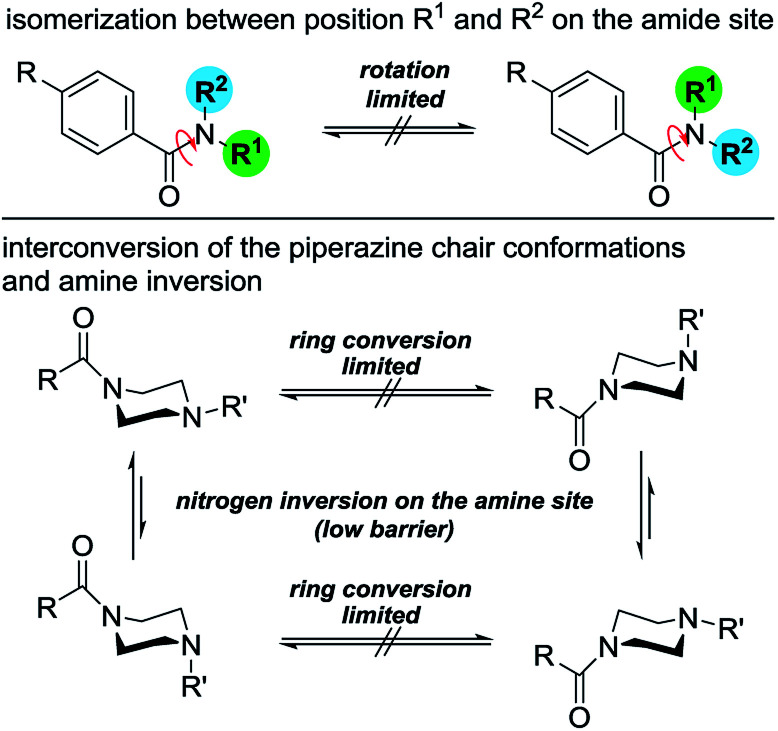
Conformational dynamics of benzoylated piperazines.

To determine the conformational behavior of these piperazine compounds, three different phenomena have to be considered (Fig. 1). The first results from the reduced rotation of the N–C-bond located in the amide moiety formed by the benzoyl residue and one or both of the tertiary piperazine nitrogen atoms. This is due to the partial double bond character, which results in the occurrence of two different rotamers. This effect is known from simple dialkylated carboxylic acid amides and also tertiary benzamides.^25,28,32,33^ The second phenomenon regards the conformation of the piperazine ring itself. Like cyclohexanes, piperazine rings can change their conformations (*e.g.* chair, half-chair, boat, twist-boat). Even though systematic experimental studies are lacking, the energetic barriers for the transformation of the chair conformations seem to be higher for piperazines than for cyclohexanes. This can be concluded from comparing the ring inversion barriers of *cis*-1,4-dimethylcyclohexane and *N*,*N*′-dimethylpiperazine, which are 40.0 kJ mol^−1^ in dichlorodifluoromethane^34^ and 55.7 kJ mol^−1^ in dichloromethane,^35^ respectively. The third phenomenon describes the pyramidal inversion of the nitrogen atom of the amine moiety; however, this should not play a major role.^36^ Subsequently, symmetrically disubstituted piperazines are discussed at first, followed by the mono-substituted and unsymmetrically substituted piperazines, due to the increasing complexity of the conformational behavior.

### Symmetrically disubstituted piperazines

**Fig. 2:**
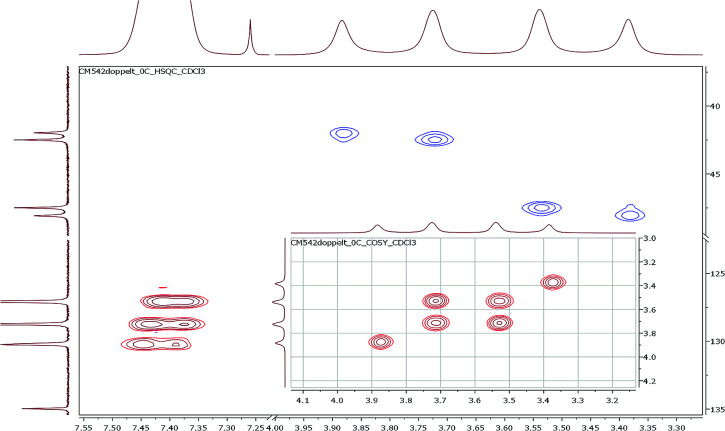
Part of the ^1^H,^13^C-HSQC and H,H-COSY (inset) spectra using the symmetrically disubstituted compound 4a at 0 °C.

To investigate the conformational behavior of symmetrically *N*,*N*′-disubstituted derivatives 4a–f, ^1^H-NMR spectra were recorded in CDCl_3_ and DMSO-*d*_6_. Normally, one summarized signal for the protons (NCH_2_) of the piperazine ring would be expected according to the symmetry of the molecule and the flexibility of the piperazine ring. This is only the case at elevated temperatures (>25–30 °C) when the molecules are flexible enough to rotate. At deeper temperatures (below 25 °C), four independent signals were found (*e.g.* for 4a: *δ* = 3.40, 3.55, 3.74, 3.90 ppm at −10 °C). H,H-COSY spectra measured in CDCl_3_ revealed that the inner two NCH_2_ signals couple in most of the cases (Fig. 2).

**Fig. 3:**
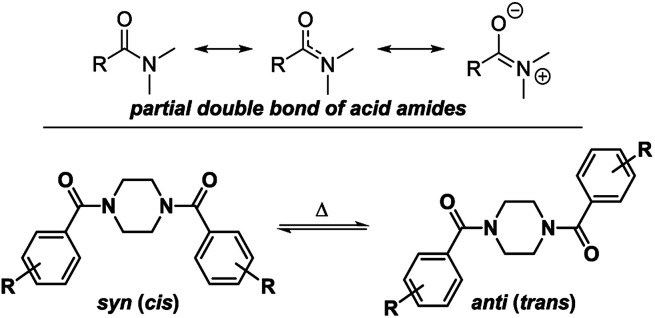
Possible rotational conformers as a result of the partial double bond character of the formed amide for symmetrically disubstituted piperazines 4a–f.

**Fig. 4:**
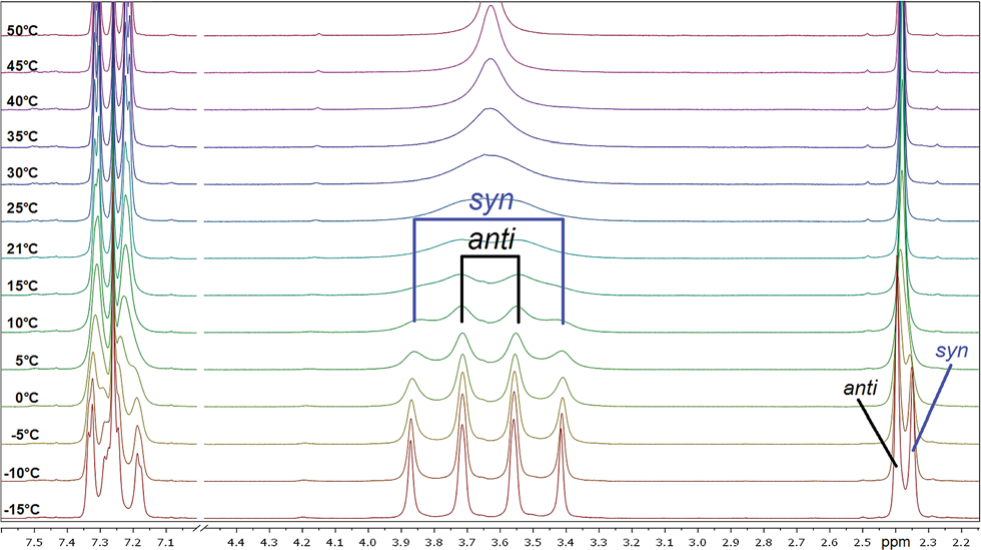
Temperature-dependent ^1^H NMR spectra of 4b measured in CDCl_3_ (region of interest from 2.00 to 7.6 ppm is shown).

These signals belong to the *anti* (*trans*) isomer whereas the two outer NCH_2_ signals belong to the *syn* (*cis*) isomer according to the results obtained by Sahoo and Chand (Fig. 3).^14^ The ^1^H–^13^C-HSQC spectrum of compound 4a measured at 0 °C verifies this behavior. Additionally, all ^13^C NMR spectra show also four independent signals (4a*syn*: *δ* = 42.0 and 48.1 ppm and *anti*: *δ* = 42.5 and 47.5 ppm at −10 °C) at low temperatures. This further evidenced the formation of two different rotational isomers. The next indication is given by the study of the methyl group and the signals in the aromatic region of 4-methylbenzoyl compound 4b. In this case, both doublets of the aromatic moiety and the methyl group (*δ* = 2.35 and 2.40 ppm) occur twice at temperatures below −10 °C (Fig. 4).

All investigated symmetrically disubstituted piperazines 4a–f show the interconversion between these two isomers: *syn* (*cis*) and *anti* (*trans*). In this case, one coalescence temperature *T*_C_ was observed, which is dependent on the solvent (Fig. 4). By warming the NMR sample, the four signals with an integration value of two hydrogen atoms each combine to one signal with a value of 8 at the coalescence point *T*_C_ resulting from a free rotation of the amide bonds present in the molecules. This allows the calculation of the Gibbs free activation energy Δ*G*^‡^. In general, the exchange rate at the coalescence temperature *T*_C_ is given by the equation *k*_exc_ = πΔ*ν*/2^1/2^.^37,38^ As a result, the Gibbs free activation energy for the rotation of the amide bond can be calculated using the Eyring eqn (II).^39^ The results are presented in Table 1.

**Table 1:** Physicochemical parameters of compounds 4a–f (measured in CDCl_3_)

No.	R	Ratio *anti* : *syn*	Δ*ν* [Hz]	*k*_exc_ [Hz]	*T*_C_ [K]	Δ*G*^‡^ [kJ mol^−1^]
4a	H	1.4 : 1	111.1 (a)	246.8 (a)	306.15	61.0 (a)
			299.4 (s)	665.1 (s)		58.5 (s)
4b	4-Me	1.5 : 1	94.2 (a)	209.3 (a)	299.15	60.0 (a)
			274.1(s)	608.9 (s)		57.3 (s)
4c	4-OMe	—	n.d.^a^	—	280.15	—
4d	4-F	1.5 : 1	103.2 (a)	229.3 (a)	296.15	59.1 (a)
			272.0 (s)	604.2 (s)		56.8 (s)
4e	4-Cl	1.6 : 1	108.6 (a)	241.2 (a)	300.15	59.8 (a)
			284.5 (s)	632.0 (s)		57.4 (s)
4f	4-Br	1.5 : 1	108.8 (a)	241.7 (a)	304.15	60.7 (a)
			285.9 (s)	635.1 (s)		58.2 (s)
4g	4-I		109.9 (a)	244.1 (a)	305.15	60.9 (a)
			287.2 (s)	638 (s)		58.4 (s)

a Not detectable; (a) *anti*; (s) *syn*.

Different Δ*ν* values each for the *syn* and for the *anti* derivatives were determined whereas only one *T*_C_ was found leading to different Δ*G*^‡^ values for the *syn* and the *anti* isomer. Notably, it was not possible to determine Δ*ν* values for the methoxy compound 4c in CDCl_3_ and for all compounds 4a–f in DMSO-*d*_6_ due to the limited cooling capacity of the NMR cooling unit and too low coalescence temperatures in relation to the melting point of the solvent, respectively.

As shown in Fig. 4, a clear distinction between the *syn* (*cis*) and *anti* (*trans*) rotamers was observed at low temperatures (<0 °C). Four independent signals occurred in the ^1^H NMR spectra of all compounds measured in CDCl_3_ with the *anti* isomer representing the energetically favored rotamer as a similar ratio of approx. 3 : 2 was determined for all compounds. In accordance to these results, all *anti* isomers have also a higher rotation barrier (59.1–61.5 kJ mol^−1^) than the corresponding *syn* derivatives (56.7–59.1 kJ mol^−1^) (Table 1).

### Mono-substituted piperazines

**Fig. 5:**
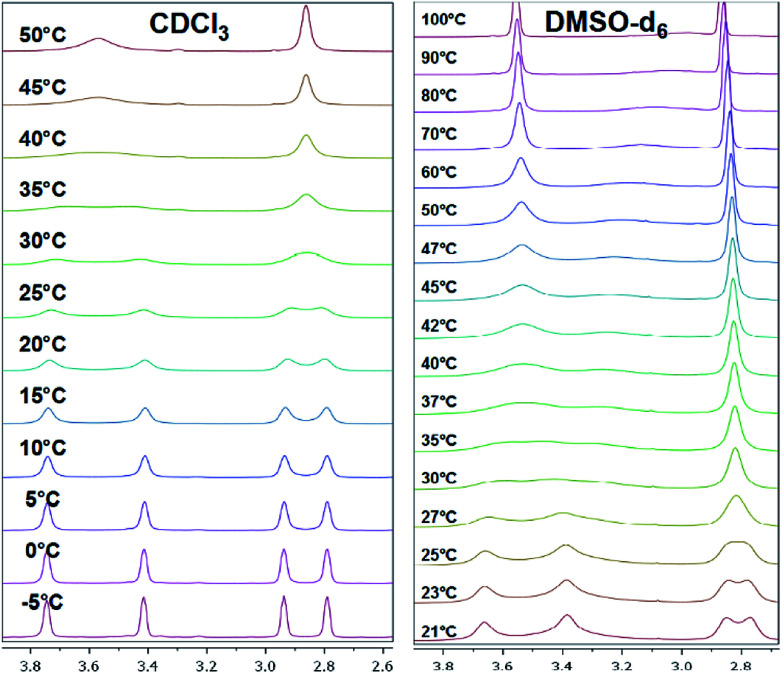
Temperature-dependent ^1^H NMR spectra of 3b measured in CDCl_3_ (left) and DMSO-*d*_6_ (right, only piperazine region from 2.6 to 4.0 ppm is shown).

Mono-*N*-benzoylated piperazines 3a–i contain only one amide bond. The other nitrogen site of the piperazine ring is unsubstituted and can therefore be considered as a typical secondary amine. Interestingly, also four signals occur at 25 °C for most of these compounds, two for the CH_2_ groups next to the amide moiety and two for the CH_2_ groups adjacent to the amine residue. As an example, the spectrum of 3b shows four broad signals (*δ* = 2.81, 2.91, 3.41, 3.70 ppm in CDCl_3_ at 25 °C) for the NCH_2_ groups of the piperazine moiety (Fig. 5). An evaluation of a H,H-COSY spectrum measured in CDCl_3_ showed again an independent coupling of two NCH_2_ groups in 3a. In this case, the equatorial proton of the amide site couples with the equatorial proton of the amine site of the piperazine ring and both axial protons couple.^40^ Furthermore, the ^1^H,^13^C-HSQC spectra showed the independent coupling of the protons to the appropriate carbon signals (ESI†). Additionally, broad signals for the carbons of the NCH_2_ groups (*e.g.*3b: *δ* = 43.3, 46.2, 49.0 ppm) are found when analyzing the ^13^C NMR spectra.

Two effects are responsible for this behavior of the mono-*N*-benzoylated piperazines 3a–i. Two different conformers (rotamers) occur due to the limited rotation of the C–N amide bond resulting from its partial double bond character (splitting of the NCH_2_ signals at the amide nitrogen atom). Thus, two distinct sets of signals are observed in the ^1^H NMR spectrum resulting from the different chemical environment that is faced by the substituents attached to the amide nitrogen atom as already shown for the symmetrically disubstituted compounds 4a–f. In general, this behavior of symmetric *N*,*N*-dialkylamide spin systems is describable as first-order process on the NMR time scale.^41^

The second effect is related to the hindered interconversion of the two piperazine ring chair conformations (Fig. 1, bottom). In the present case, this process is also decreased at room temperature (splitting of the NCH_2_ signals at the amine nitrogen atom). For simple non-acylated piperazines^42–44^ and morpholines^45^ the observation of distinct conformers by NMR is restricted to low temperatures (<−10 °C). The different energy barriers of both effects resulted in two different coalescence points. To further investigate the conformational behavior of the piperazines and to determine these energies, temperature-dependent NMR experiments^39^ were performed for all derivatives in CDCl_3_ and in DMSO-*d*_6_. Results are presented in Tables 2 and 3.

**Table 2:** Physicochemical parameters of compounds 3a–i measured in CDCl_3_

No.	R	Δ*ν* [Hz]	*k*_exc_ [Hz]	*T*_C_ [K]	Δ*G*^‡^ [kJ mol^−1^]
3a	H	90.9 (amine)	246.8 (amine)	308.15 (amine)	62.0 (amine)
		217.8 (amide)	665.1 (amide)	318.15 (amide)	61.7 (amide)
3b	4-Me	88.5 (amine)	196.6 (amine)	303.15 (amine)	61.0 (amine)
		198.3 (amide)	440.5 (amide)	313.15 (amide)	61.0 (amide)
3c	4-OMe	80.0 (amine)	177.7 (amine)	288.15 (amine)	58.1 (amine)
		165.1 (amide)	366.8 (amide)	295.15 (amide)	57.8 (amide)
3d	4-F	83.3 (amine)	185 (amine)	298.15 (amine)	60.1 (amine)
		204 (amide)	453.2 (amide)	309.15 (amide)	60.1 (amide)
3e	4-Cl	86.6 (amine)	192.4 (amine)	305.15 (amine)	61.5 (amine)
		217.1 (amide)	482.3 (amide)	319.15 (amide)	62.0 (amide)
3f	4-Br	87.5 (amine)	194.4 (amine)	304.15 (amine)	61.2 (amine)
		214.1 (amide)	475.6 (amide)	316.15 (amide)	61.4 (amide)
3g	4-I	87.3 (amine)	193.9 (amine)	310.15 (amine)	62.5 (amine)
		215.0 (amide)	477.6 (amide)	315.15 (amide)	61.2 (amide)
3h	4-NO_2_	92.14 (amine)	204.7 (amine)	320.15 (amine)	64.4 (amine)
		267.2 (amide)	596.6 (amide)	n.d. (amide)	n.d. (amide)
3i	3-Br	81.4 (amine)	180.8 (amine)	309.15 (amine)	62.5 (amine)
		215.13 (amide)	477.9 (amide)	319.15 (amide)	62.0 (amide)

**Table 3:** Physicochemical parameters of compounds 3b, d–i measured in DMSO-*d*_6_

No.	R	Δ*ν* [Hz]	*k*_exc_ [Hz]	*T*_C_ [K]	Δ*G*^‡^ [kJ mol^−1^]
3b	4-Me	48.28 (amine)	107.3 (amine)	299.15 (amine)	61.6 (amine)
		166.58 (amide)	370.0 (amide)	309.15 (amide)	60.6 (amide)
3d	4-F	n.d. (amine)	n.d. (amine)	296.15 (amine)	n.d. (amine)
		168.9 (amide)	375.2 (amide)	308.15 (amide)	60.4 (amide)
3e	4-Cl	59.36 (amine)	131.9 (amine)	307.15 (amine)	62.8 (amine)
		190.68 (amide)	423.6 (amide)	321.15 (amide)	62.7 (amide)
3f	4-Br	59.6 (amine)	132.4 (amine)	305.15 (amine)	62.4 (amine)
		189.1 (amide)	420.1 (amide)	330.15 (amide)	64.6 (amide)
3g	4-I	58.2 (amine)	129.3 (amine)	304.15 (amine)	62.2 (amine)
		183.2 (amide)	407 (amide)	318.15 (amide)	62.2 (amide)
3h	4-NO_2_	75.5 (amine)	167.7 (amine)	325.15 (amine)	66.0 (amine)
		236.1 (amide)	524.5 (amide)	338.15 (amide)	65.6 (amide)
3i	3-Br	62.60 (amine)	139.1 (amine)	311.65 (amine)	63.7 (amine)
		196.23 (amide)	435.9 (amide)	328.15 (amide)	64.1 (amide)

When monitoring the piperazine derivatives 3a–i over a range of up to 80 K, the four signals of the NCH_2_ groups gradually disappear and coalesce to the two expected signals (one signal for the amide site and one for the amine site of the piperazine ring) at increased temperatures (*T*_C,amine_ = 303 K, *T*_C,amide_ = 313 K for 3b in CDCl_3_). The determined Δ*G*^‡^ values for both barriers lie between 57 and 66 kJ mol^−1^ with higher values obtained in DMSO-*d*_6_ (Tables 2 and 3). The determined values for the amide bond rotation barriers are in agreement to those reported for other benzamides, which are in the range of 57–73 kJ mol^−1^.^46–48^ The observed solvent effect on the rotational barriers is also in accord to literature reports and can be explained by the fact that more polar solvent molecules can better stabilize the zwitterionic canonical form of the amide bond (Fig. 3 top right).^25–27,49^

**Fig. 6:**
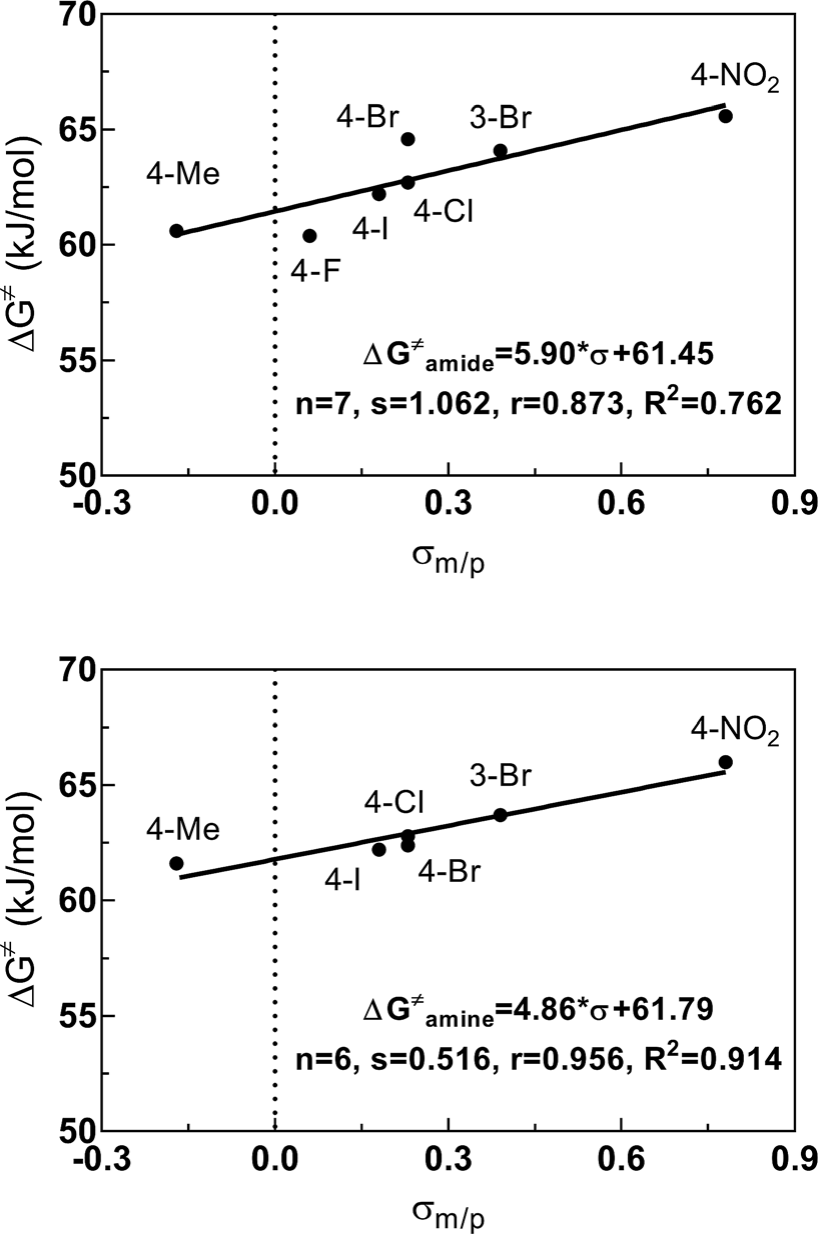
Relationship between the electronic properties (*σ*_m/p_) of the substituents at the benzoyl residue and the activation energies Δ*G*^‡^ for the amide (top) and amine (bottom) sites of compounds 3 determined in DMSO-*d*_6_. Plot of Δ*G*^‡^ = *f*(*σ*_m/p_) using the Δ*G*^‡^ values listed in Table 3 and the following values for *σ*_m_: 0.39 (3-Br) and for *σ*_p_: −0.17 (Me), 0.06 (F), 0.23 (Cl), 0.23 (Br), 0.18 (I) and 0.78 (NO_2_).^51^ Regression analysis was performed by linear regression.

As obvious from Tables 2 and 3, the Δ*G*^‡^ values for the amide bond and for the amine site strongly depend on the substituent at the benzoyl residue. Generally, substituents in *meta* and *para* position to the amide bond influence its rotational barrier by inductive and mesomeric effects *via* the π-system of the phenyl ring. To quantify this interaction, correlation analyses were performed using Hammett *σ* constants (*σ*_m/p_, *σ*_p_^−^, *σ*_p_^+^). For DMSO-*d*_6_, the correlations of the Δ*G*^‡^ values *versus σ*_m/p_ are shown in Fig. 6 (for further correlations see ESI†). All correlations resulted in fair to good linear relationships with a positive slope. Thus, the rotational barriers increase with increasing electron withdrawing character of the substituents defined by their *σ*_m/p_ values. Consequently, substituents with a higher electron withdrawing effect promote the formation of the zwitterionic form of the amide bond and/or destabilize the transition state of the isomerization, in which the carbonyl bond is oriented perpendicular to the nitrogen lone electron pair. Both effects will lead to a higher activation enthalpy for the C–N bond rotation. Worth of note, for the activation energies determined in DMSO-*d*_6_, similar correlations were obtained for the different Hammett *σ* constants, whereas for the energies determined in CDCl_3_, the *σ*_p_^+^ values, which describe the capacity of substituents to delocalize a positive charge,^50^ give significant better correlations than using the other Hammett *σ* constants for both amide and amine sites (see ESI†). This result can be interpreted in the context of the fact that the zwitterionic ground state is less stabilized in this solvent compared to DMSO-*d*_6_ and therefore the donating effect of the *para*-substituent to the positively charged carbonyl carbon in the transition state is more predominant in CDCl_3_.

**Fig. 7:**
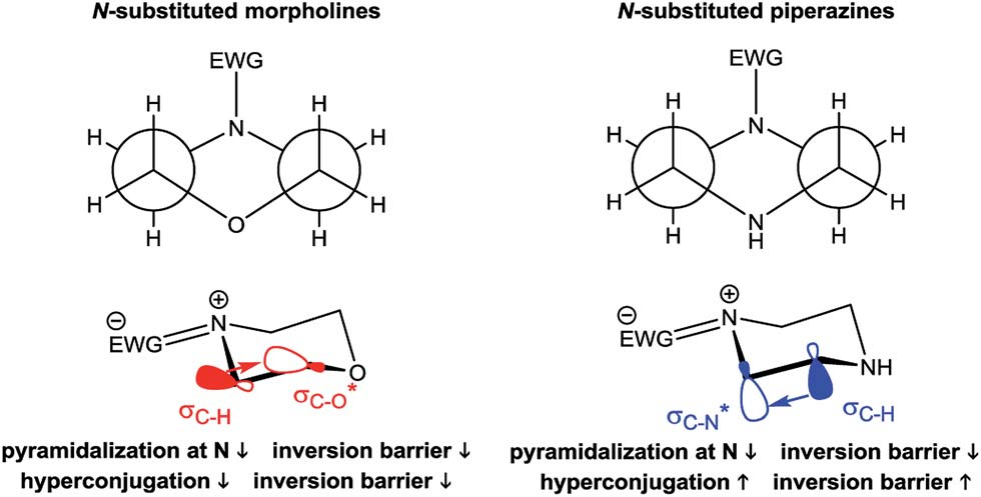
Proposed relationship between ring flattening and hyperconjugation (*gauche* effect) in *N*-substituted morpholines and piperazines. The Newman projections shown on top are indicating the *gauche* arrangement of both heteroatoms in the ring. Below are shown the chair models and the main direction of charge transfer by the *gauche* effect and the influence of electron-withdrawing groups (EWG) at the ring nitrogen. See text for further explanation.

The derived relationships are in accordance to studies on the rotational barriers for the tertiary amide bond in *N*,*N*-dimethyl benzamides^25–27^ and *N*,*N*-diethylbenzamides.^28^ The strengthened zwitterionic character of the amide bond in turn results in a reduced conformational flexibility of the piperazine ring and requires higher energies for the ring conversion. Worth of note, irrespective of the kind of electronic substituent parameter, the free activation enthalpies for the ring inversion barriers (Δ*G*^‡^ of amine) gave significantly better correlations than the barriers for amide bond rotation. To the best of our knowledge, systematic investigations of the substituent effects on the ring inversion barriers in piperazines have not been reported so far. The observed trend that the free activation enthalpies increase with higher electron pull of the substituent at the benzoyl moiety is in contrast to results obtained for *N*-substituted morpholines, for which a higher degree of conjugation at the ring nitrogen results in lower ring inversions barriers.^52–54^ This effect has been mainly attributed to larger bond angles around the ring nitrogen upon introduction of acyl substituents, which diminishes the angle strain in the transition state. Furthermore, we hypothesize that the stereoelectronic *gauche* effect, which should be in operation in both piperazine and morpholine rings, acts towards stabilization of the chair conformations. In morpholines, the *gauche* effect should act mainly through charge transfer from the equatorial *σ*_C–H_ orbitals at C-3 and C-5 into the anti-bonding 
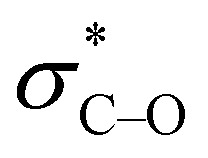
 orbitals (Fig. 7). Consequently, substituents at nitrogen with increasing electron pull will lower this hyperconjugation as the electron density in the respective *σ*_C–H_ orbitals is reduced, and therefore the ring inversion barriers are decreased. Thus, ring flattening and decreased hyperconjugation act additively.

In contrast, in the piperazine derivatives of compound series 3, in which the oxygen atom is replaced by less electronegative nitrogen, the charge transfer by the *gauche* effect is acting mainly in the counter direction, *i.e.* from the *σ*_C–H_ orbitals at C-3 and C-5 into the anti-bonding 
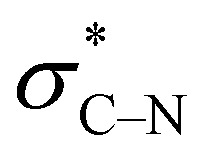
 orbitals involving the N-1 nitrogen. Higher positive partial charge at N-1 should increase this hyperconjugation, as it has been found for 2-fluoroethylamine derivatives,^55^ and therefore the chair form should be more stabilized by this effect. Consequently, the influence of electron-withdrawing group at the nitrogen in *N*-monosubstituted piperazines on ring flattening and hyperconjugation by the *gauche* effect are acting in opposite directions. In this context, it is worth of note that the activation energy for *N*-benzoylmorpholine is with 31.0 kJ mol^−1^ (in dichlorofluoromethane) considerably lower than that found for compound 3a (61.02 kJ mol^−1^ in CDCl_3_).^52–54^ The influence of the substituents on the *gauche* effect seems to be stronger than that on ring flattening with regards to the inversion barriers in piperazines, which results in a correlation with positive slope between the Hammett substituent constants and the free activation enthalpies for ring inversion (Fig. 6 and ESI†). This effect can be further illustrated by comparing the ring inversion barriers of methoxy- and nitro-substituted compounds 3c and 3h in CDCl_3_, which exhibit the lowest and highest values for Δ*G*^‡^, respectively (see Table 2; 58.08 *vs.* 64.43 kJ mol^−1^).

### Unsymmetrically substituted piperazines

Based on the results for the mono-substituted piperazines, we were interested in the NMR behavior of mono-benzoyl-substituted piperazines where the secondary amine group is replaced by a tertiary amine group. For this purpose, 4-nitrophenylpiperazine was reacted with different benzoyl chlorides to yield the series of analogues 6a–k. Dynamic NMR experiments were performed in CDCl_3_. Again, four signals occur at 25 °C for most of these compounds.

For the *meta* and *para* substituted derivatives, the Δ*G*^‡^ values for the rotation of the amide bond and ring inversion were derived (Table 4). Generally, the activation energies for both phenomena are up to 2.5 kJ mol^−1^ lower compared to those energies for the respective mono-substituted piperazines 3. The presence of the phenyl group at the former secondary amine results in a partial double bond character of the N–C_1,Phenyl_ bond due to delocalization of the nitrogen lone pair towards the substituent. The nitro group in *para* position to the piperazine nitrogen (push–pull system) further favors this delocalization. This increases the bond angle around the nitrogen atom and lowers the ring inversion barriers (ring flattening). Simultaneously, the electron density within the *σ*_C–H_ orbital is reduced, which might decline the charge transfer to the 
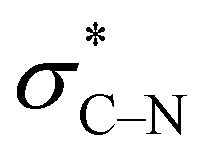
 orbital and thus, lowers the barrier for ring inversion (Fig. 7). Similar to compounds 3, correlation analyses of the Δ*G*^‡^ values with the Hammett *σ* constants resulted in linear relationships with positive slopes. This indicates, that the *gauche* effect is still predominant compared to the ring flattening.

**Table 4:** Physicochemical parameters of compounds 6a–d, f, h–k

No.	R	Solvent	Δ*ν* [Hz]	*k*_exc_ [Hz]	*T*_C_ [K]	Δ*G*^‡^ [kJ mol^−1^]
6a	H	CDCl_3_	99.0 (amine)	219.9 (amine)	299.15 (amine)	59.9 (amine)
			183.9 (amide)	408.5 (amide)	307.15 (amide)	60.0 (amide)
6b	4-Me	CDCl_3_	96.3 (amine)	213.9 (amine)	294.15 (amine)	58.9 (amine)
			160.2 (amide)	355.9 (amide)	302.15 (amide)	59.3 (amide)
6c	4-OMe	CDCl_3_	85.9 (amine)	190.8 (amine)	281.15 (amine)	56.4 (amine)
			126.6 (amide)	281.2 (amide)	286.15 (amide)	56.6 (amide)
6d	4-F	CDCl_3_	90.2 (amine)	200.4 (amine)	291.15 (amine)	58.4 (amine)
			166.5 (amide)	369.9 (amide)	297.15 (amide)	58.2 (amide)
6f	4-Br	CDCl_3_	92.5 (amine)	205.5 (amine)	297.15 (amine)	59.6 (amine)
			177.0 (amide)	393.2 (amide)	305.15 (amide)	59.6 (amide)
6h	4-NO_2_	CDCl_3_	99.9 (amine)	221.9 (amine)	309.15 (amine)	61.9 (amine)
			232.0 (amide)	515.4 (amide)	323.15 (amide)	62.6 (amide)
6i	3-Br	CDCl_3_	88.3 (amine)	196.2 (amine)	300.15 (amine)	60.4 (amine)
			183.0 (amide)	406.5 (amide)	311.15 (amide)	60.8 (amide)
6j	2-Br	DMSO-*d*_6_	74.5 (amine)	165.5 (amine)	>393.15 (amine)	— (amine)
6k	2-NO_2_	CDCl_3_	232.4^a^	516.3	312.15^a^	60.4
6k	2-NO_2_	DMSO-*d*_6_	105.3 (amine)	233.9 (amine)	391.15 (amine)	78.9 (amine)
			262.7 (amide)	583.6 (amide)	>393.15 (amide)	— (amide)

a Coalescence of two protons from one of the NCH_2_ groups of the amide site, complete coalescence of the amide site was not detectable.

**Fig. 8:**
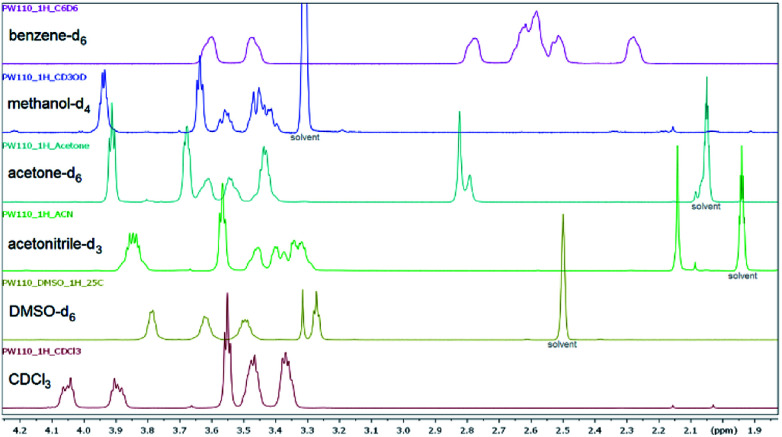
^1^H NMR spectra (aliphatic region) of *ortho*-bromo derivative 6j measured in six different deuterated solvents at 25 °C to show the pattern of the piperazine protons in dependence of the solvent.

Furthermore, the substituent parameter *σ*_p_^+^ gives again significantly better correlations than parameters *σ*_p_ and *σ*_p_^−^ for both amide and amine sites (ESI†). The three bromobenzoyl derivatives 6f, 6i, and 6j were used to compare the influence of the position (*ortho*, *meta* and *para*). Changing the position of the bromine atom from *para* (6f) to *meta* (6i) did not result in significantly different rotational barriers of the amide bond as the value of Δ*G*^‡^ is approx. 60 kJ mol^−1^ for both compounds. In contrast, a different chemical shift pattern of the NCH_2_ groups in CDCl_3_ and DMSO-*d*_6_ was found for 6j due to the steric hindrance of the bromine in *ortho* position and therefore a reduced rotation about the C_*ipso*_–C(O) bond.^56^ Thus, additional NMR spectra of 6j were recorded in acetonitrile-*d*_3_, acetone-*d*_3_, benzene-*d*_6_, and methanol-*d*_4_ (Fig. 8, ESI†), which revealed a different splitting of the NCH_2_ protons depending on the solvent. In CDCl_3_ the protons are further split (ESI†) whereas in DMSO-*d*_6_ three broadened signals and a triplet with two protons each was found. H,H-COSY revealed, that two of them coupled indicating that the two downfield-shifted signals are belonging to the amide site and the two other belonging to the amine site of the piperazine ring.

A similar situation was found for the nitro derivatives 6k and 6h. Derivative 6k, with the nitro group in *ortho* position, shows five independent signals in the ^1^H NMR spectrum at temperatures below 38 °C in contrast to *para*-nitro derivative 6h which shows the expected four signals when measured in CDCl_3_. Interestingly, both compounds 6k and 6h show four signals when measured in DMSO-*d*_6_. This can be explicated by similar mesomeric and electronic effects of both compounds making *ortho* and *para* position comparable. Thus, the behavior of 6k can be again ascribed to the additional steric hindrance of the nitro group in *ortho* position of the benzoyl residue resulting in additional rotamers due to the hindered rotation of the C_*ipso*_–C(O) bond. As also found for *ortho*-bromo compound 6j, two protons from one of the NCH_2_ groups of the amide site are split and a coalescence temperature of *T*_C_ = 41 °C and a Δ*G*^‡^ = 60.4 kJ mol^−1^ was found (ESI†). The complete coalescence of amide site and amine site, respectively, of 6j and 6k was not reached due to the limitations imposed by the experimental setup.

**Fig. 9:**
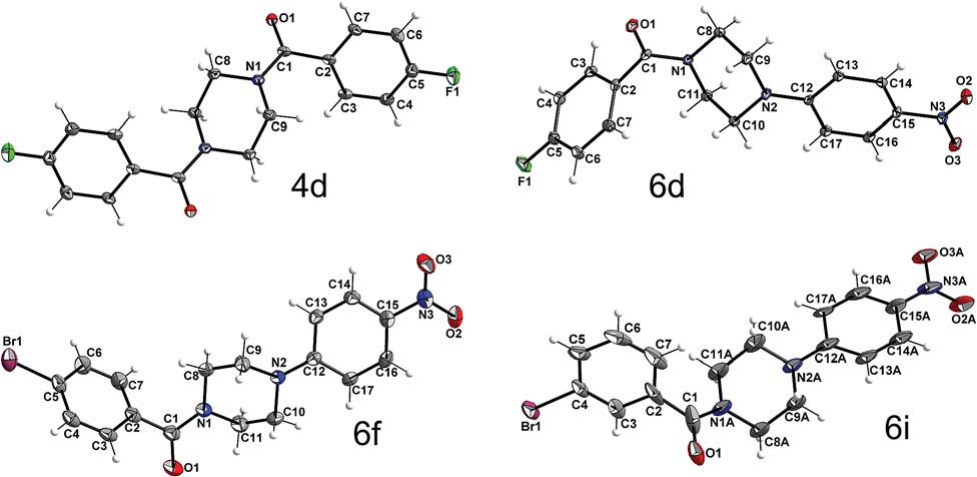
Molecular structures of 4d, 6d, 6f, and 6i with atom labeling scheme (ORTEP plots, thermal displacement ellipsoids are shown with 50% probability at −150 °C).

**Fig. 10:**
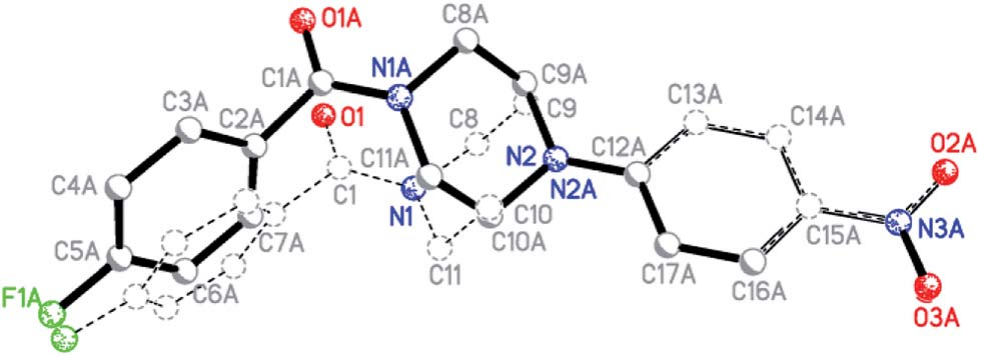
Superimposition fit of the two conformers, which exist in the ratio of 1 : 1 in the solid-state structure of compound 6d (label A marks the atoms of the second conformer; not all labels shown).

To obtain further insight into the causes for the limited rotation of the amide bond and ring inversion as studied by NMR, single crystals of 4d, 6d, 6f, and 6i were used to determine the solid-state structures of these compounds. They all consist of neutral molecules, packed in monoclinic lattices with space groups *P*2_1_/*n* (4d and 6d), *P*2_1_/*c* (6i), and *C*2/*c* (6f). The molecular structures with atom numbering schemes are shown in Fig. 9. Whereas the molecules of 6d, 6f, and 6i, which comprise the asymmetric unit, are located on general positions and not located on any symmetry element (besides identity), the center of the piperazine ring of 4d is located on an inversion center, such that the molecule has inversion symmetry. For compounds 6d, 6f, and 6i, two conformers exist in a 1 : 1 ratio, which are not superimposable due to the inversion centers in the crystal which are located outside of the molecules. Fig. 10 shows the superposition of the two ringconformers of 6d, showing the different orientations, especially of the piperazine moieties, when the 4-nitro-phenyl rings are fitted on top of each other. For compound 6i the structure is affected by some disorder, which could best be described using a split model. Relevant crystallographic details of all structures are collected in the ESI.†

An interesting question concerning these structures is if the rotational barrier as observed in the NMR spectra (see above) goes along with structural indication of at least double bond character of the carbonyl C–N bond of compounds 6d, 6f, and 6i. An inspection of the bond lengths and angles, exemplarily for 6d, shows that the N1 as well as the N2 atoms are surrounded by carbon atoms in a close to planar manner. N1 deviates out of the plane formed by C1, C8 and C11 carbon atoms by 0.07 Å. A similar value of 0.02 Å was determined for the distance of N2 from the plane defined by C9, C10, and C12. The average C–N–C bond angles at the piperazine nitrogen atoms are found to be close to 120° (119.8° for N1 and 120.0° for N2). In this context, it should be noted that for the tertiary amine nitrogen in 4-(3-butyn-1-yl)-1-(4-fluorobenzoyl)piperazine, an average C–N–C bond angle of 109.8° was recently determined from a single-crystal structure of this compound,^21^ which clearly highlights the broadening of the bond angle by the 4-nitrophenyl group and therefore the considerable depyramidalization of the corresponding nitrogen atom. Moreover, the increased flattening of the piperazine ring by the 4-nitrophenyl substituent is illustrated by comparing the bond angle at the piperazine nitrogen atom inside the ring (C9(A)–N2(A)–C10(A)); Table 5. Whereas for 4-(3-butyn-1-yl)-1-(4-fluorobenzoyl)-piperazine a bond angle of 109.0° was determined, those values for compounds 6d, f, i were significantly higher (111.4–113.8°). The distinct involvement of the amide and amine nitrogen in partial double bonds is further expressed by the respective bond lengths, as the bond length of N1 to the carbonyl C1 atom is significantly shorter than to the other two C atoms, N1–C1: 1.355(1) Å *vs.* N1–C8: 1.460(1) Å, N1–C11: 1.462(1) Å. Similarly, the N2 contact to the aromatic carbon atom C12 is shorter than the other two contacts: N2–C12: 1.367(1) Å *vs.* N2–C9: 1.463(1), N2–C10: 1.524(1) Å. In contrast, a length of 1.464 Å was determined for the corresponding C–N bond in 4-(3-butyn-1-yl)-1-(4-fluorobenzoyl)piperazine.^21^ These structural features imply the overlap of the free electron pair of N2 with the π-system of the electron-deficient nitrophenyl residue and explain the lower ring inversion barriers of compounds 6 compared to their monosubstituted analogues 3 because of the flattened C–N–C bond angle. Moreover, the increased positive charge at the amine nitrogen might attenuate the hyperconjugation shown in Fig. 7 and therefore contribute to the destabilization of the chair conformation. Selected geometrical data of all four XRD structurally characterized compounds are summarized in Table 5.

**Table 5:** Geometrical parameters around N1 and N2 in the four XRD structurally characterized compounds

	4d	6d	6f	6i
Δ(plane) N1/Å^a^	0.08	0.07	0.01	0.00
Δ(plane) N2/Å	—	0.02	0.18	0.237
Av. bond angle N1/°	119.7	119.8	120.0	120.0
Av. bond angle N2/°	—	120.0	118.5	117.3
Bond angle C9(A)–N2(A)–C10(A)	—	112.2	111.4	113.8
Bond angle C9(A)–N2(A)–C12(A)	—	123.7	122.0	118.4
Bond angle C10(A)–N2(A)–C12(A)	—	124.1	122.0	119.7
N1–C1/Å^b^	1.3569(8)	1.355(1)	1.356(2)	1.373(6)
N1–C8/Å	1.4588(8)	1.460(1)	1.456(2)	1.458(7)
N1–C11/Å	1.4593(8)	1.462(1)	1.452(2)	1.474(6)
N2–C12/Å	—	1.367(1)	1.372(2)	1.394(9)
N2–C9/Å	—	1.463(1)	1.463(2)	1.465(6)
N2–C10/Å	—	1.524(1)	1.456(2)	1.46(1)

a Δ(plane) N1: deviation of the position of N1 out of the plane of surrounding three C atoms.

b For 6i: the labels for N1, N2, and C8–C16 need to be replaced by N1A, N2A, C8A–C16A, because of the atom naming in the disorder split model.

### Conformations of inhibitors for transglutaminase 2 (TGase 2)

**Scheme 3:**
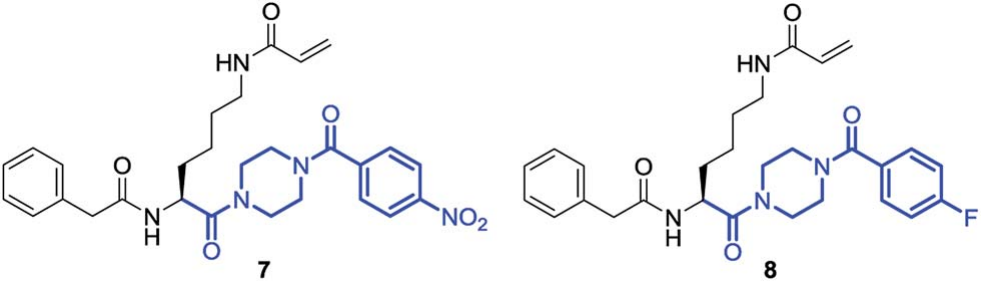
Structures of TGase 2 inhibitors 7 and 8.

Amongst others, the structural element of *N*,*N*′-unsymmetrically substituted piperazines is found in TGase 2-inhibitors^57–59^ as shown in Scheme 3 (the structural element is highlighted in blue). The beneficial influence of the piperazine ring as linker between the benzoyl moiety and the remaining parts of the molecule on the inhibitory activity towards TGase 2 has been demonstrated.^58^ Fluorine derivative 8 is a potential ^18^F-radiotracer for PET (positron emission tomography) imaging of TGase 2 when fluorine-18 (β^+^-emitter, half life 110 min) is introduced and compound 7 could be potentially used as precursor (starting compound) for radiolabeling *via* nucleophilic introduction of [^18^F]fluoride. The preparation and analyses of both compounds 7 and 8 is published elsewhere together with their inhibitory activities.^57^

**Fig. 11:**
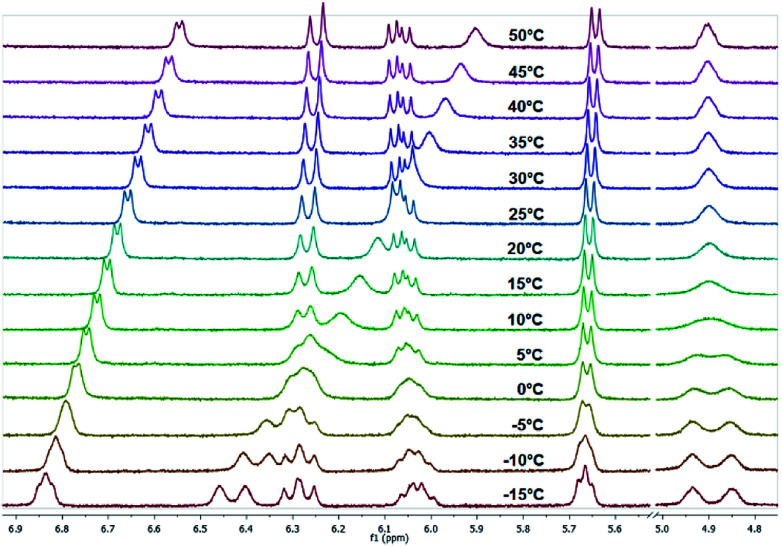
Temperature-dependent ^1^H NMR spectra of fluorine compound 8 measured in CDCl_3_ (region of interest from 4.70 to 7.00 ppm is shown).

As expected from the previous investigations, two rotamers were observed for compounds 7 and 8 due to the partial double bond character of tertiary amides that involve the piperazine ring. A good indication for this phenomenon is the proton of the α-carbon atom in the lysine residue. In case of compound 7, two broad singlets were observed for this proton at 25 °C in CDCl_3_ as well as in DMSO-*d*_6_. The determination of the rotation barriers for both compounds was accomplished in CDCl_3_ on the basis of the signals for the C_α_H proton. The temperature- dependent spectra of fluorine compound 8 are presented in Fig. 11. The coalescence temperature *T*_C_ was found at 7 °C for fluorine compound 8 and at 28 °C for nitro compound 7 in CDCl_3_. The rotation barrier Δ*G*^‡^ was calculated to be 57.4 kJ mol^−1^ for 8 and 61.7 kJ mol^−1^ for 7. The higher rotation barrier for compound 7 is in accordance to the previous results for compounds 3 and 6, as the nitro group increases the partial double bond character of the benzoyl amide bond compared to fluorine.

## Experimental

### General

All chemicals were purchased from commercial suppliers and used without further purification unless otherwise specified. Anhydrous THF was purchased from Acros. NMR spectra of all compounds were recorded on an Agilent DD2-400 MHz or DD2-600 MHz NMR spectrometer with ProbeOne. Chemical shifts of the ^1^H, ^19^F, and ^13^C spectra were reported in parts per million (ppm) using TMS as internal standard for ^1^H/^13^C and CFCl_3_ for ^19^F spectra at 25 °C unless otherwise stated. Spectra were calibrated to their solvent signals. Dynamic NMR measurements were carried out in a temperature range of 253–393 K using the Agilent VT accessory with digital temperature controller. *T*_C_ was estimated from the NMR spectrum showing the collapse of the methylene signals from the piperazine moiety and Δ*ν* values were extracted from the maximum split signals of the piperazine methylene protons, which was not varied with decreasing temperature. Values of the exchange rate, *k*_exc_, were calculated using equation *k*_exc_ = πΔ*ν*/2^1/2^.^37,38^ Subsequently, values of the free activation energy, Δ*G*^‡^, were obtained by solving the Eyring eqn (I) for Δ*G*^‡^(II).^37,39^ In accordance to other studies on amide *cis*/*trans* isomerization, the transmission coefficient, *κ*, was assumed to be 1.^60^I
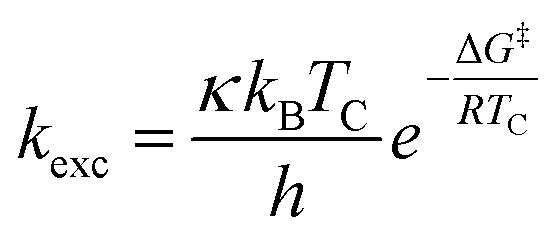
II
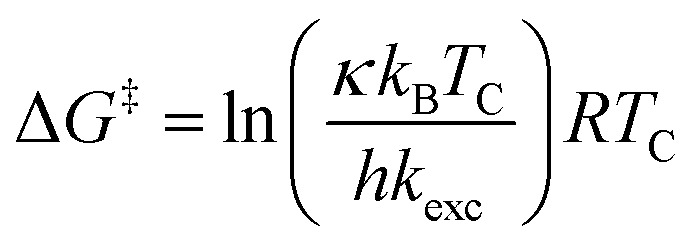


Mass spectrometric (MS) data were obtained on a Xevo TQ-S mass spectrometer (Waters) by electron spray ionization (ESI). The melting points were determined on a Galen III melting point apparatus (Cambridge Instruments & Leica) and are uncorrected. Microanalyses were carried out with a LECO CHNS 932 elemental analyzer. Chromatographic separations and TLC detections were performed using Merck Silica Gel 60 (63–200 μm) and Merck Silica Gel 60 F254 sheets, respectively. TLCs were developed by visualization under UV light (*λ* = 254 nm). Single crystal X-ray diffraction data of 4d, 6d, 6f, and 6i were collected with a Bruker-Nonius Apex-II-diffractometer using graphite-monochromated Mo-K_α_ radiation (*λ* = 0.71073 Å). Diffraction measurements were done at −150 °C (4d, 6d, 6i) or 23 °C (6f). The structures was solved by direct methods and refined against *F*^2^ by full-matrix least-squares using the program suites from G. M. Sheldrick.^61–63^ All non-hydrogen atoms were refined anisotropically; all hydrogen atoms were placed on geometrically calculated positions and refined using riding models. CCDC-1816843 (4d), CCDC-1588729 (6d), CCDC-1825578 (6f), and CCDC-1838271 (6i) contain the supplementary crystallographic data.

### Chemistry

#### General procedure for the preparation of compounds 3a–i and 4a–f

Piperazine (2 equiv.) was dissolved in absolute ethanol or THF, the respective acid chloride (1 equiv.) was slowly added at 0 °C and the mixture was allowed to stir at r.t. overnight. Afterwards, the solvent was removed and the crude mixture of products was purified *via* column chromatography yielding the mono and the diacylated compound from the one-pot reaction.

#### 1-Benzoyl-4-(4-nitrophenyl)piperazine 6a^64^

1-(4-Nitrophenyl)-piperazine (5, 150 mg, 0.72 mmol) and Et_3_N (100 mg, 0.99 mmol) were dissolved in anhydrous THF (5 mL), the solution was cooled to 0 °C and benzoyl chloride (2a, 90 mg, 0.66 mmol) was slowly added. The resulting mixture was warmed to r.t. and stirred for another 4 h. After reaction control by TLC, THF was changed by ethyl acetate (15 mL), water (15 mL) was added and the aqueous layer was extracted with ethyl acetate (3 × 15 mL). The combined organic layers were dried over Na_2_SO_4_, the solvent was removed and the crude product was purified *via* column chromatography (petroleum ether : ethyl acetate 1 : 1) to yield 6a as yellow solid (150 mg, 73%). Mp 153–156 °C; *R*_f_ = 0.30 (petroleum ether : ethyl acetate 1 : 2); ^1^H NMR (400 MHz, CDCl_3_): *δ* = 3.33–4.04 (m, 8H, NCH_2_), 6.83 (d, ^3^*J* = 9.3 Hz, 2H, Ar–H), 7.40–7.49 (m, 5H, Ar–H), 8.13 (d, ^3^*J* = 9.3 Hz, 2H, Ar–H) ppm; ^13^C NMR (101 MHz, CDCl_3_): *δ* = 42.1 (br. s, NCH_2_), 47.3 (br. s, NCH_2_), 113.3, 126.1, 127.3, 128.8, (4× CH_Ar_), 130.3, 135.2, 139.3, 154.6 (4× C_Ar_), 170.7 (C=O). MS (ESI+) *m*/*z*: 312 (M^+^ + H, 100%).

#### 1-(4-Methylbenzoyl)-4-(4-nitrophenyl)piperazine 6b

1-(4-Nitrophenyl)piperazine (5, 150 mg, 0.72 mmol) and Et_3_N (100 mg, 0.99 mmol) were dissolved in anhydrous THF (5 mL), the solution was cooled to 0 °C and 4-methylbenzoyl chloride (2b, 102 mg, 0.66 mmol) was slowly added. The resulting mixture was warmed to r.t. and stirred for another 4 h. After reaction control by TLC, THF was changed by ethyl acetate (15 mL), water (15 mL) was added and the aqueous layer was extracted with ethyl acetate (3 × 15 mL). The combined organic layers were dried over Na_2_SO_4_, the solvent was removed and the crude product was purified *via* column chromatography (petroleum ether : ethyl acetate 1 : 1) to yield 6a as yellow solid (120 mg, 56%). Mp 193 °C; *R*_f_ = 0.20 (petroleum ether : ethyl acetate 1 : 2); ^1^H NMR (600 MHz, CDCl_3_): *δ* = 2.39 (s, 3H, CH_3_), 3.33–4.00 (m, 8H, NCH_2_), 6.82 (d, ^3^*J* = 9.1 Hz, 2H, Ar–H), 7.24 (d, ^3^*J* = 7.8 Hz, 2H, Ar–H), 7.34 (d, ^3^*J* = 9.1 Hz, 2H, Ar–H), 8.12 (d, ^3^*J* = 9.1 Hz, 2H, Ar–H) ppm; ^13^C NMR (150 MHz, CDCl_3_): *δ* = 21.6 (CH_3_), 41.9 (br. s, NCH_2_), 47.2 (br. s, NCH_2_), 113.2, 126.0, 127.4, 129.4 (4× CH_Ar_), 132.2, 139.2, 140.6, 154.6 (4× C_Ar_), 170.9 (C=O). MS (ESI+) *m*/*z*: 326 (M^+^ + H, 100%).

#### 1-(4-Methoxybenzoyl)-4-(4-nitrophenyl)piperazine 6c

1-(4-Nitrophenyl)piperazine (5, 150 mg, 0.72 mmol) and Et_3_N (100 mg, 0.99 mmol) were dissolved in anhydrous THF (5 mL), the solution was cooled to 0 °C and 4-methoxybenzoyl chloride (2c, 112 mg, 0.66 mmol) was slowly added. The resulting mixture was warmed to r.t. and stirred for another 4 h. After reaction control by TLC, THF was changed by ethyl acetate (15 mL), water (15 mL) was added and the aqueous layer was extracted with ethyl acetate (3 × 15 mL). The combined organic layers were dried over Na_2_SO_4_, the solvent was removed and the crude product was purified *via* column chromatography (petroleum ether : ethyl acetate 1 : 1) to yield 6c as yellow solid (220 mg, 98%). Mp 133 °C; *R*_f_ = 0.24 (petroleum ether : ethyl acetate 1 : 2); ^1^H NMR (600 MHz, CDCl_3_): *δ* = 3.46 (br. s, 4H, NCH_2_), 3.70–3.90 (m, 7H, NCH_2_ + CH_3_), 6.82 (d, ^3^*J* = 9.3 Hz, 2H, Ar–H), 6.93 (d, ^3^*J* = 8.6 Hz, 2H, Ar–H), 7.42 (d, ^3^*J* = 8.6 Hz, 2H, Ar–H), 8.12 (d, ^3^*J* = 9.3 Hz, 2H, Ar–H) ppm; ^13^C NMR (150 MHz, CDCl_3_): *δ* = 47.3 (br. s, NCH_2_), 55.5 (CH_3_), 113.2, 114.0, 126.1, 129.4 (4× CH_Ar_), 127.1, 139.2, 154.6, 161.3 (4× C_Ar_), 170.7 (C=O). MS (ESI+) *m*/*z*: 342 (M^+^ + H, 100%).

#### 1-(4-Fluorobenzoyl)-4-(4-nitrophenyl)piperazine 6d

1-(4-Nitrophenyl)piperazine (5, 150 mg, 0.72 mmol) and Et_3_N (100 mg, 0.99 mmol) were dissolved in anhydrous THF (5 mL), the solution was cooled to 0 °C and 4-fluorobenzoyl chloride (2d, 104 mg, 0.66 mmol) was slowly added. The resulting mixture was warmed to r.t. and stirred for another 4 h. After reaction control by TLC, THF was changed by ethyl acetate (15 mL), water (15 mL) was added and the aqueous layer was extracted with ethyl acetate (3 × 15 mL). The combined organic layers were dried over Na_2_SO_4_, the solvent was removed and the crude product was purified *via* column chromatography (petroleum ether : ethyl acetate 1 : 1) to yield 6c as yellow solid (200 mg, 93%). Mp 173 °C; *R*_f_ = 0.52 (chloroform : ethanol 20 : 1); ^1^H NMR (600 MHz, CDCl_3_): *δ* = 3.31–4.02 (m, 8H, NCH_2_), 6.83 (d, ^3^*J* = 8.8 Hz, 2H, Ar–H), 7.12 (t, ^3^*J* = 8.4 Hz, ^3^*J*_H,F_ = 8.4 Hz, 2H, Ar–H), 7.42 (d, ^3^*J* = 8.4 Hz, ^4^*J*_H,F_ = 5.3 Hz, 2H, Ar–H), 8.12 (d, ^3^*J* = 8.8 Hz, 2H, Ar–H) ppm; ^13^C NMR (150 MHz, CDCl_3_): *δ* = 44.7, 50.0 (2× br. s, NCH_2_), 115.8 (CH_Ar_), 118.4 (d, ^2^*J*_C,F_ = 21.9 Hz, CH_Ar_), 128.6 (CH_Ar_), 132.2 (d, ^3^*J*_C,F_ = 8.3 Hz, CH_Ar_), 131.7 (d, ^4^*J*_C,F_ = 3.4 Hz, C_Ar_), 157.1 (C_Ar_), 166.3 (d, ^1^*J*_C,F_ = 246.4 Hz, C_Ar_), 172.3 (C=O); ^19^F NMR (565 MHz, CDCl_3_): *δ* = −109.4. MS (ESI+) *m*/*z*: 330 (M^+^ + H, 100%).

#### 1-(4-Bromobenzoyl)-4-(4-nitrophenyl)piperazine 6f

1-(4-Nitrophenyl)piperazine (5, 226 mg, 1.09 mmol) and Et_3_N (150 mg, 1.48 mmol) were dissolved in anhydrous THF (6 mL), the solution was cooled to 0 °C and 4-bromobenzoyl chloride (2f, 218 mg, 0.99 mmol) was slowly added. The resulting mixture was warmed to r.t. and stirred for another 4 h. After reaction control by TLC, THF was changed by ethyl acetate (15 mL), water (15 mL) was added and the aqueous layer was extracted with ethyl acetate (3 × 15 mL). The combined organic layers were dried over Na_2_SO_4_, the solvent was removed and the crude product was purified *via* column chromatography (petroleum ether : ethyl acetate 1 : 1) to yield 6i as yellow solid (145 mg, 37%). Mp 187–189 °C; *R*_f_ = 0.24 (petroleum ether : ethyl acetate 1 : 1); ^1^H NMR (400 MHz, CDCl_3_): *δ* = 3.25–4.01 (m, 8H, NCH_2_), 6.82 (d, ^3^*J* = 9.4 Hz, 2H, Ar–H), 7.32 (d, ^3^*J* = 7.6 Hz, ^3^*J* = 8.4 Hz, 1H, Ar–H), 7.57 (d, ^3^*J* = 8.4 Hz, 1H, Ar–H), 8.11 (d, ^3^*J* = 9.4 Hz, 2H, Ar–H) ppm; ^13^C NMR (150 MHz, CDCl_3_): *δ* = 41.9 (br. s, NCH_2_), 47.3 (br. s, NCH_2_), 113.3, 126.0, 129.0, 132.0 (4× CH_Ar_), 124.7, 134.0, 139.3, 154.5 (C_Ar_), 169.7 (C=O). MS (ESI+) *m*/*z*: 390 (M^+^ + H, ^35^Br 100%), 392 (M^+^ + H, ^37^Br, 100%).

#### 1-(4-Nitrobenzoyl)-4-(4-nitrophenyl)piperazine 6h^65^

1-(4-Nitrophenyl)piperazine (5, 150 mg, 0.72 mmol) and Et_3_N (100 mg, 0.99 mmol) were dissolved in anhydrous THF (5 mL), the solution was cooled to 0 °C and 4-nitrobenzoyl chloride (2h, 122 mg, 0.66 mmol) was slowly added. The resulting mixture was warmed to r.t. and stirred for another 4 h. After reaction control by TLC, THF was changed by ethyl acetate (15 mL), water (15 mL) was added and the aqueous layer was extracted with ethyl acetate (3 × 15 mL). The combined organic layers were dried over Na_2_SO_4_, the solvent was removed and the crude product was purified *via* column chromatography (petroleum ether : ethyl acetate 1 : 1) to yield 6x as yellow solid (190 mg, 81%). Mp 145 °C; *R*_f_ = 0.14 (petroleum ether : ethyl acetate 1 : 1); ^1^H NMR (400 MHz, CDCl_3_): *δ* = 3.27–3.73 (m, 6H, NCH_2_), 3.97 (br. s, 2H, NCH_2_), 6.86 (d, ^3^*J* = 9.3 Hz, 2H, Ar–H), 7.63 (d, ^3^*J* = 8.8 Hz, 2H, Ar–H), 8.16 (d, ^3^*J* = 9.3 Hz, 2H, Ar–H), 8.32 (d, ^3^*J* = 8.8 Hz, 2H, Ar–H), ppm; ^13^C NMR (150 MHz, CDCl_3_): *δ* = 47.2 (br. s, NCH_2_), 47.8 (br. s, NCH_2_), 113.7, 124.2, 126.1, 128.4, (4× CH_Ar_), 126.1, 141.3, 148.8, 154.4 (4× C_Ar_), 168.3 (C=O). MS (ESI+) *m*/*z*: 357 (M^+^ + H, 100%).

#### 1-(3-Bromobenzoyl)-4-(4-nitrophenyl)piperazine (6i)

1-(4-Nitrophenyl)piperazine (5, 150 mg, 0.72 mmol) and Et_3_N (100 mg, 0.99 mmol) were dissolved in anhydrous THF (5 mL), the solution was cooled to 0 °C and 3-bromobenzoyl chloride (2i, 144 mg, 0.66 mmol) was slowly added. The resulting mixture was warmed to r.t. and stirred for another 4 h. After reaction control by TLC, THF was changed by ethyl acetate (15 mL), water (15 mL) was added and the aqueous layer was extracted with ethyl acetate (3 × 15 mL). The combined organic layers were dried over Na_2_SO_4_, the solvent was removed and the crude product was purified *via* column chromatography (petroleum ether : ethyl acetate 1 : 1) to yield 6i as yellow solid (240 mg, 93%). Mp 133 °C; *R*_f_ = 0.40 (petroleum ether : ethyl acetate 1 : 2); ^1^H NMR (400 MHz, CDCl_3_): *δ* = 3.25–4.07 (m, 8H, NCH_2_), 6.84 (d, ^3^*J* = 9.3 Hz, 2H, Ar–H), 7.32 (dd, ^3^*J* = 7.6 Hz, ^3^*J* = 8.4 Hz, 1H, Ar–H), 7.37 (d, ^3^*J* = 7.6 Hz, 1H, Ar–H), 7.57–7.62 (m, 2H, Ar–H), 8.13 (d, ^3^*J* = 9.3 Hz, 2H, Ar–H) ppm; ^13^C NMR (150 MHz, CDCl_3_): *δ* = 41.9 (br. s, NCH_2_), 47.3 (br. s, NCH_2_), 113.4, 122.9, 125.8, 126.1, 130.3, 130.4, 133.4, 137.2, 139.4, 168.9 (C=O). MS (ESI+) *m*/*z*: 390 (M^+^ + H, ^35^Br 100%), 392 (M^+^ + H, ^37^Br, 100%).

#### 1-(2-Bromobenzoyl)-4-(4-nitrophenyl)piperazine (6j)

1-(4-Nitrophenyl)piperazine (5, 226 mg, 1.09 mmol) and Et_3_N (150 mg, 1.48 mmol) were dissolved in anhydrous THF (6 mL), the solution was cooled to 0 °C and 2-bromobenzoyl chloride (2j, 218 mg, 0.99 mmol) was slowly added. The resulting mixture was warmed to r.t. and stirred for another 4 h. After reaction control by TLC, THF was changed by ethyl acetate (15 mL), water (15 mL) was added and the aqueous layer was extracted with ethyl acetate (3 × 15 mL). The combined organic layers were dried over Na_2_SO_4_, the solvent was removed and the crude product was purified *via* column chromatography (petroleum ether : ethyl acetate 1 : 1) to yield 6j as yellow solid (210 mg, 54%). Mp 179 °C; *R*_f_ = 0.20 (petroleum ether : ethyl acetate 1 : 1); ^1^H NMR (600 MHz, CDCl_3_): *δ* = 3.32–3.41 (m, 2H, NCH_2_), 3.42–3.51 (m, 2H, NCH_2_), 3.55 (t, ^3^*J* = 5.2 Hz, 2H, NCH_2_), 3.85–3.94 (m, 1H, NCH_2_), 4.01–4.09 (m, 1H, NCH_2_), 6.82 (d, ^3^*J* = 9.4 Hz, 2H, Ar–H), 7.26–7.32 (m, 2H, Ar–H), 7.39 (t, ^3^*J* = 7.4 Hz, 1H, Ar–H), 7.60 (d, ^3^*J* = 8.3 Hz, 2H, Ar–H), 8.11 (d, ^3^*J* = 9.4 Hz, 2H, Ar–H) ppm; ^13^C NMR (150 MHz, CDCl_3_): *δ* = 43.7, 48.6, 49.5, 50.1 (4× NCH_2_), 115.9 (CH_Ar_), 121.8 (C_Ar_), 128.6, 130.4, 130.6, 133.3, 135.6 (5× CH_Ar_), 140.0, 141.8, 157.1 (3× C_Ar_), 170.4 (C=O). MS (ESI+) *m*/*z*: 390 (M^+^ + H, ^35^Br 100%), 392 (M^+^ + H, ^37^Br, 100%).

#### 1-(2-Nitrobenzoyl)-4-(4-nitrophenyl)piperazine 6k

1-(4-Nitrophenyl)piperazine (5, 150 mg, 0.72 mmol) and Et_3_N (100 mg, 0.99 mmol) were dissolved in anhydrous THF (5 mL), the solution was cooled to 0 °C and 2-nitrobenzoyl chloride (2x, 122 mg, 0.66 mmol) was slowly added. The resulting mixture was warmed to r.t. and stirred for another 4 h. After reaction control by TLC, THF was changed by ethyl acetate (15 mL), water (15 mL) was added and the aqueous layer was extracted with ethyl acetate (3 × 15 mL). The combined organic layers were dried over Na_2_SO_4_, the solvent was removed and the crude product was purified *via* column chromatography (petroleum ether : ethyl acetate 1 : 1) to yield 6k as yellow solid (200 mg, 85%). Mp 182–186 °C; *R*_f_ = 0.09 (petroleum ether : ethyl acetate 1 : 1); ^1^H NMR (600 MHz, CDCl_3_): *δ* = 3.37–3.47 (m, 4H, NCH_2_), 3.57–3.64 (m, 2H, NCH_2_), 3.80–3.96 (br. m, 1H, NCH_2_), 3.85–3.94 (m, 1H, NCH_2_), 4.08–4.25 (br. m, 1H, NCH_2_), 6.85 (d, ^3^*J* = 9.3 Hz, 2H, Ar–H), 7.44 (d, ^3^*J* = 7.6 Hz, 1H, Ar–H), 7.63 (t, ^3^*J* = 8.3 Hz, 1H, Ar–H), 7.76 (t, ^3^*J* = 7.4 Hz, 1H, Ar–H), 8.15 (d, ^3^*J* = 9.3 Hz, 2H, Ar–H), 8.23 (d, ^3^*J* = 8.3 Hz, 1H, Ar–H) ppm; ^13^C NMR (150 MHz, CDCl_3_): *δ* = 29.9, 41.5, 46.2, 47.1 (4× NCH_2_), 115.9 (CH_Ar_), 113.8, 123.6 (2× C_Ar_), 125.1, 126.1, 128.2, 130.4, 134.8 (4× CH_Ar_), 142.8, 145.6 (2× C_Ar_), 166.8 (C=O). MS (ESI+) *m*/*z*: 357 (M^+^ + H, 100%).

## Conclusions

We systematically investigated the influence of various functional groups and their position at the benzoyl residue on the ^1^H NMR behavior and conformational transitions in benzamide systems containing piperazines. For mono-benzoyl substituted piperazines, positive correlations of the activation energies for amide bond rotation and ring inversion with the Hammett *σ* constants of the substituents at the benzoyl group have been derived. The remarkable correlation between these parameters and the inversion barrier in piperazines has been discussed considering reported findings for *N*-substituted morpholines. In this context, a model for the relationship of ring flattening and *gauche* effect and their impact on the ring inversion barrier is proposed. This sheds light on the complex influences on energetic barriers for conformational transitions in acylated heterocycloaliphates, which are commonly encountered structural elements in active pharmaceutical ingredients and potential drug molecules. Furthermore, activation energies were determined for benzoyl substituted 4-nitrophenyl piperazines, which were slightly lower compared to the monosubstituted series with substituent effects that are in accordance to the results obtained for mono-benzoyl substituted piperazines. Furthermore, the influence of the substituent position at the benzoyl moiety on the ^1^H NMR behavior was demonstrated for this compound series.

Additionally, two TGase 2 inhibitors as pharmacologically relevant compounds exhibiting benzoylpiperazine partial structures were investigated. Δ*G*^‡^ values of 61.7 kJ mol^−1^ and 57.4 kJ mol^−1^ were found for the compounds containing the 4-nitrobenzoyl and 4-fluorobenzoyl residues, respectively.

## Conflicts of interest

There are no conflicts to declare.

## Supplementary Material

RA-008-C8RA09152H-s001

RA-008-C8RA09152H-s002
