# Supplementary material for: NMR-based investigations of acyl-functionalized piperazines concerning their conformational behavior in solution
Source: RSC Adv. 2018 Dec 6;8(71):40921–33. doi: 10.1039/c8ra09152h (PMC9091627; doi:10.1039/c8ra09152h)
Supplement: RA-008-C8RA09152H-s001 [file RA-008-C8RA09152H-s001.pdf]

Electronic Supplementary Material (ESI) for RSC Advances.
This journal is © The Royal Society of Chemistry 2018

Supporting Information for

NMR-based investigations of acyl-functionalized piperazines concerning their conformational behavior†

Robert Wodtke,[a] Janine Steinberg,[a,b] Martin Köckerling,[c] Reik Löser,[*a,b] and Constantin Mamat[*a,b]

a Institut für Radiopharmazeutische Krebsforschung, Helmholtz-Zentrum Dresden-Rossendorf, Bautzner Landstraße 400, D-01328 Dresden, Germany

b Fakultät Chemie und Lebensmittelchemie, Technische Universität Dresden, D-01062 Dresden, Germany

c Institut für Chemie – Anorganische Festkörperchemie, Universität Rostock, Albert-Einstein-Straße 4a, D-18059 Rostock, Germany

Content:

Temperature-depended ^{1}H NMR spectra of compounds **3a-i** S2

Temperature-depended ^{1}H NMR spectra of compounds **4a-g** S11

^{1}H/^{13}C NMR spectra and Temperature-depended ^{1}H NMR spectra of compounds **6a-k** S18

Equations of correlation analyses and summary of used substituent parameters.............................S44

Crystal data and structure refinement for compounds **4d**, **6d**, **6f**, and **6i** S46

N-Benzoylpiperazine (**3a**)

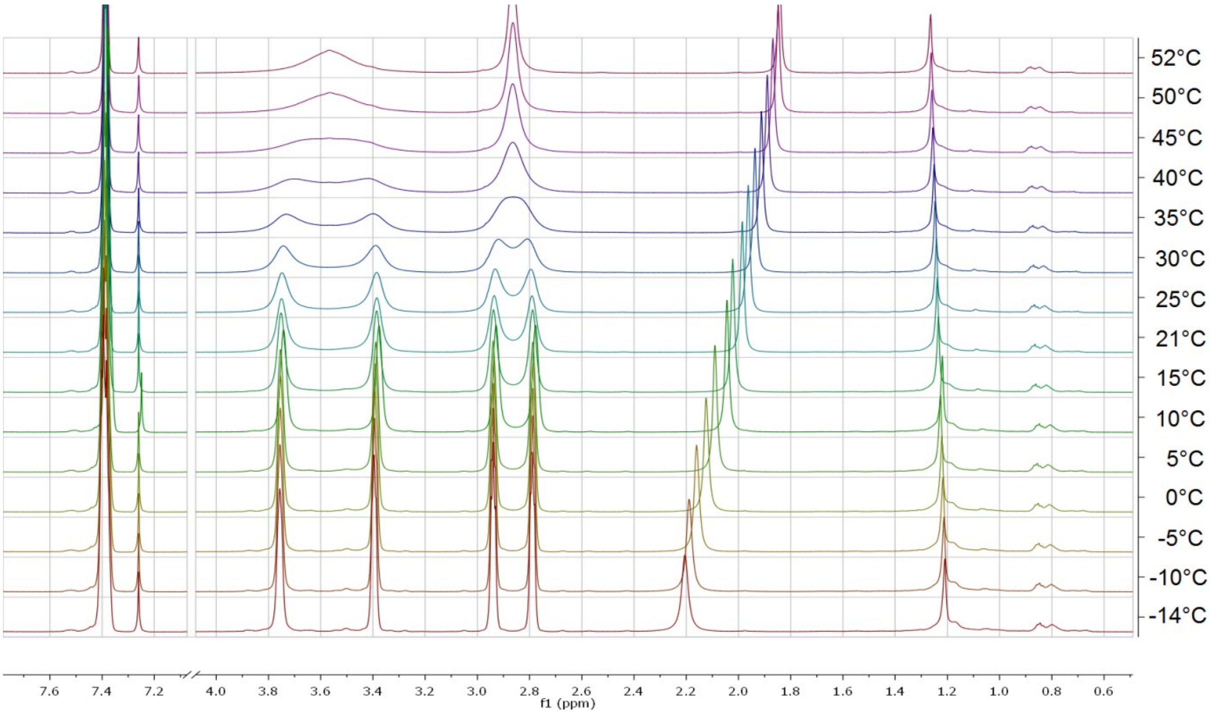

Figure S1. Temperature-depended ^{1}H NMR spectrum of compound **3a** measured in $CDCl_3$.

N-(4-Methylbenzoyl)piperazine (**3b**)

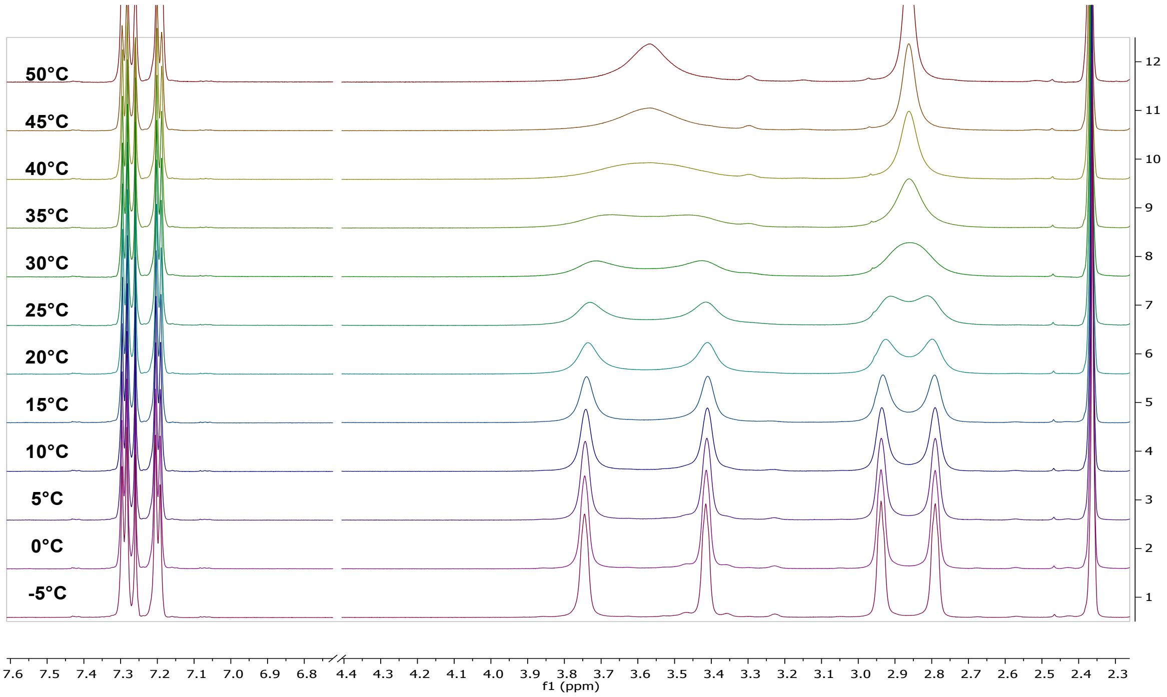

Figure S2. Temperature-depended ^{1}H NMR spectrum of compound **3b** measured in $CDCl_3$.

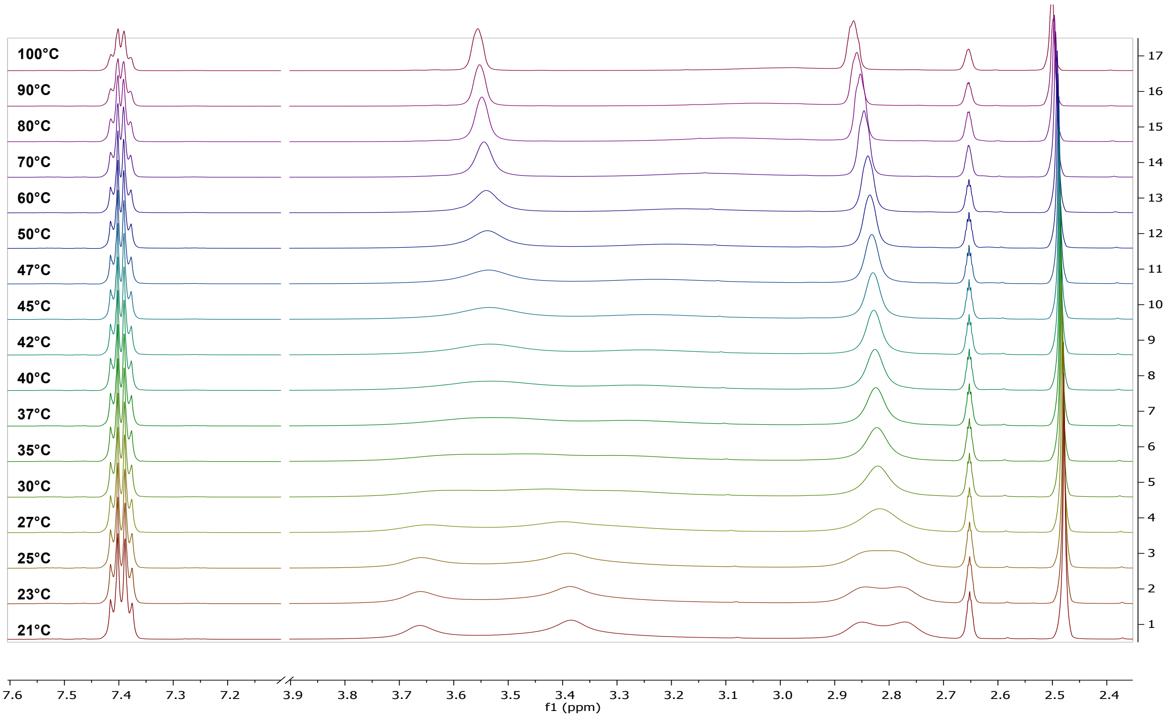

Figure S3. Temperature-depended ^{1}H NMR spectrum of compound **3b** measured in DMSO-d_6.

N-(4-Methoxybenzoyl)piperazine (**3c**)

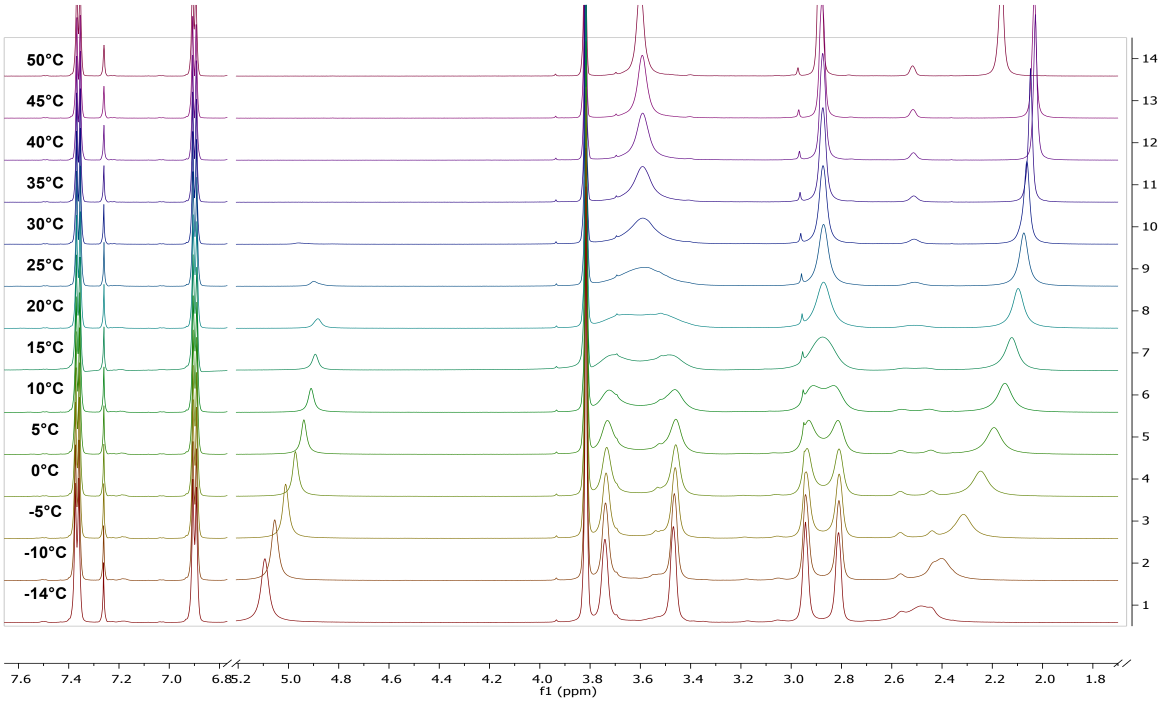

Figure S4. Temperature-depended ^{1}H NMR spectrum of compound **3c** measured in $CDCl_3$.

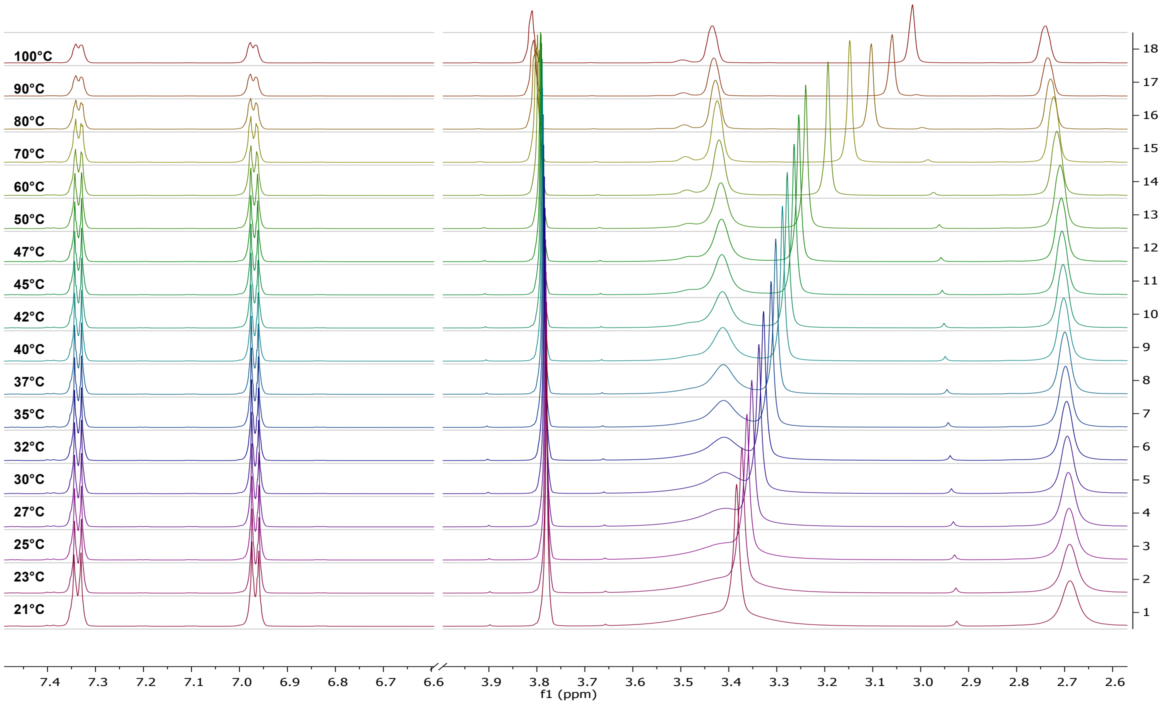

Figure S6. Temperature-depended ^{1}H NMR spectrum of compound **3c** measured in DMSO-d_6.

N-(4-Fluorobenzoyl)piperazine (**3d**)

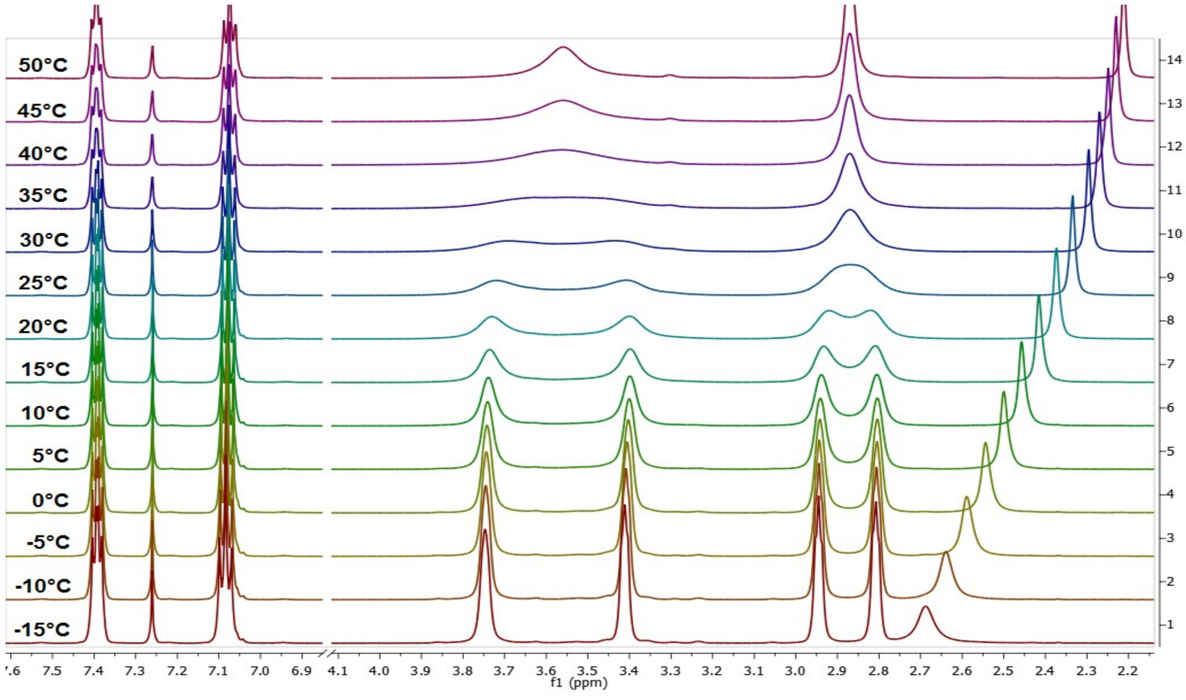

Figure S7. Temperature-depended ^{1}H NMR spectrum of compound **3d** measured in $CDCl_3$.

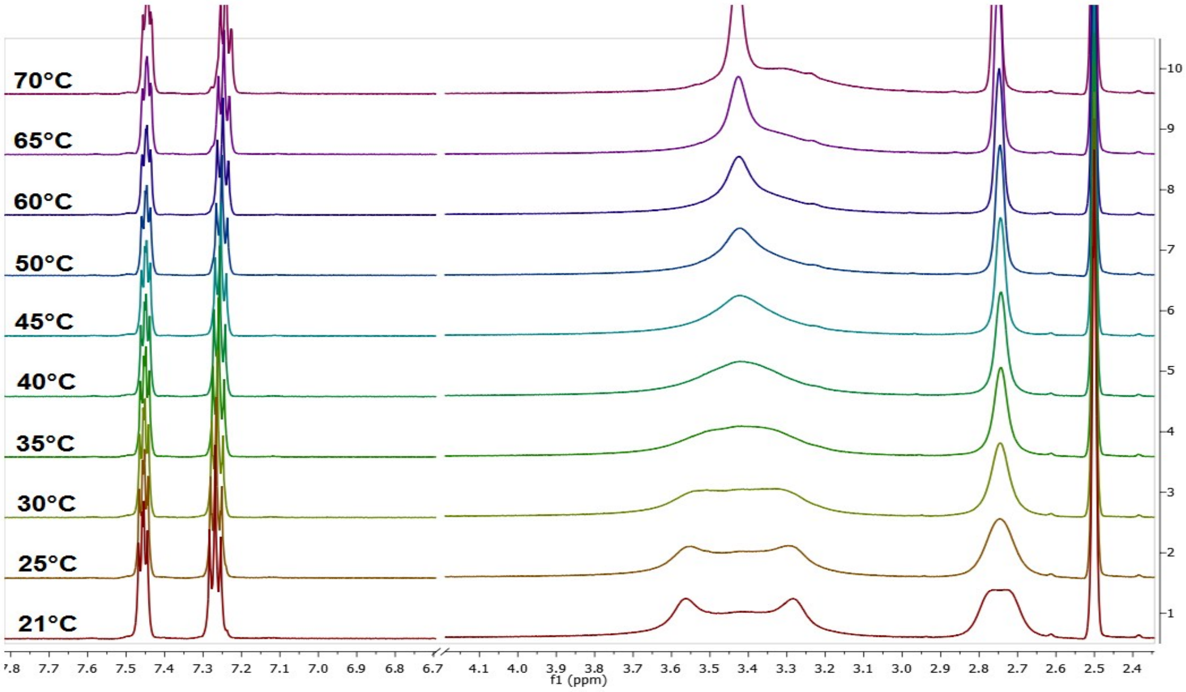

Figure S8. Temperature-depended ^{1}H NMR spectrum of compound **3d** measured in DMSO-d_6.

N-(4-Chlorobenzoyl)piperazine (**3e**)

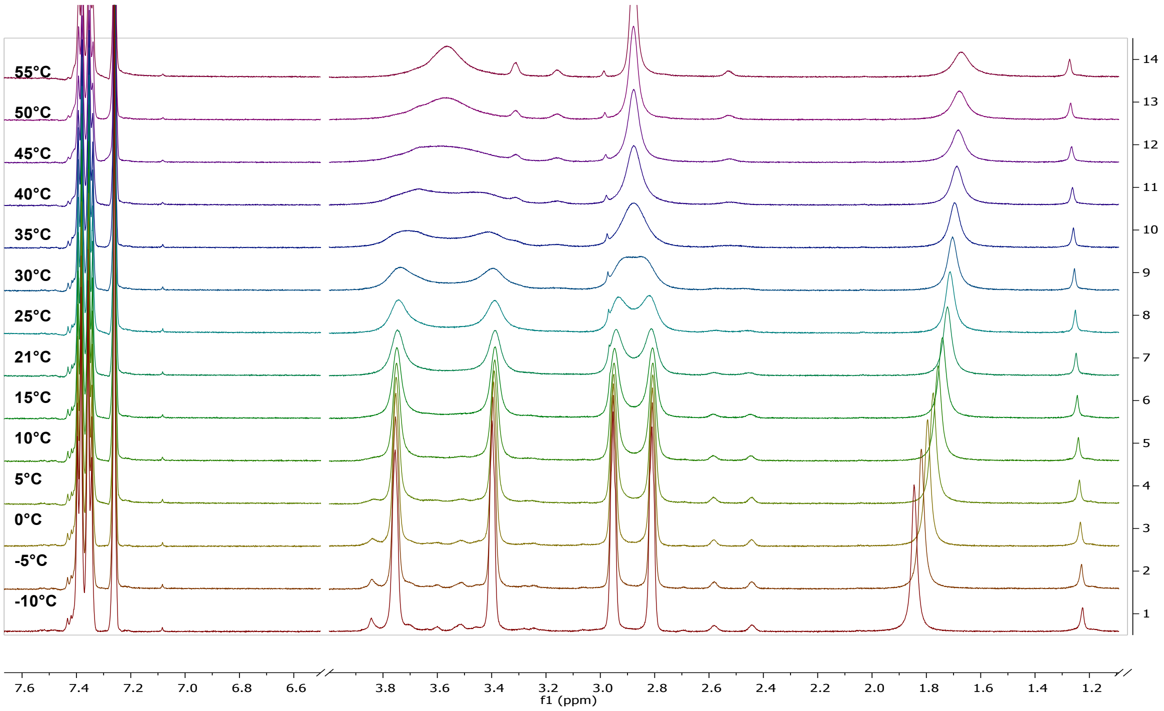

Figure S9. Temperature-depended ^{1}H NMR spectrum of compound **3e** measured in $CDCl_3$.

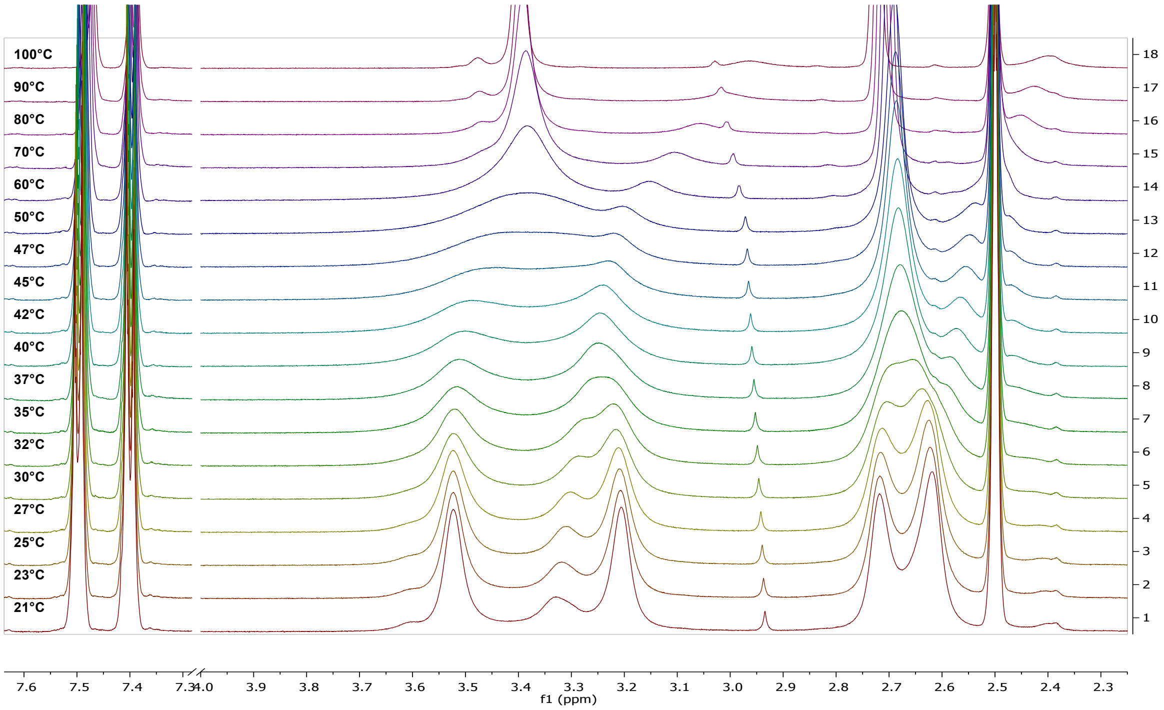

Figure S10. Temperature-depended ^{1}H NMR spectrum of compound **3e** measured in DMSO-d_6.

N-(4-Bromobenzoyl)piperazine (**3f**)

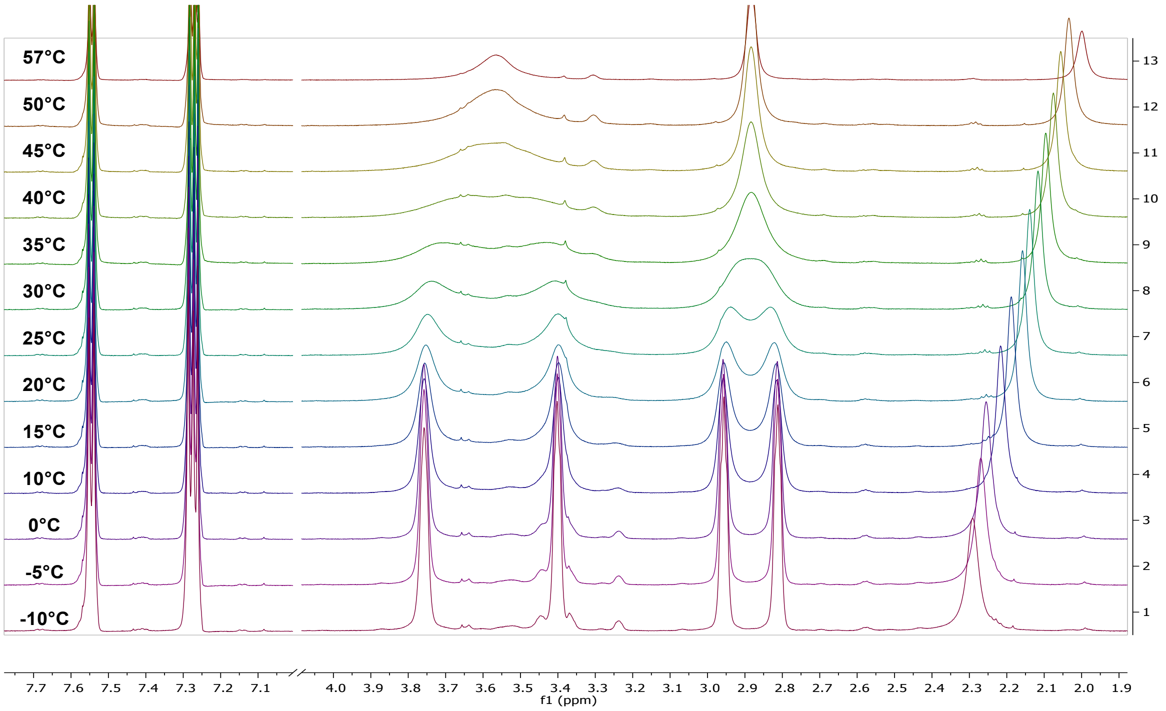

Figure S11. Temperature-depended ^{1}H NMR spectrum of compound **3f** measured in $CDCl_3$.

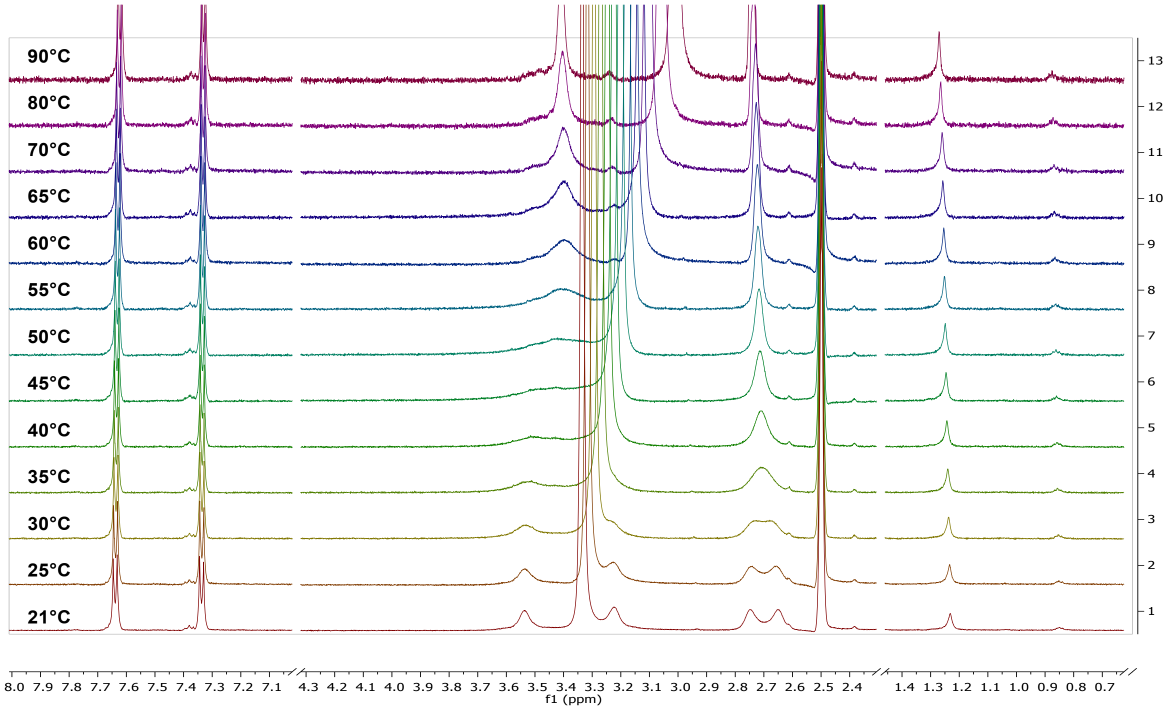

Figure S12. Temperature-depended ^{1}H NMR spectrum of compound **3f** measured in DMSO-d_6.

N-(4-Iodobenzoyl)piperazine (**3g**)

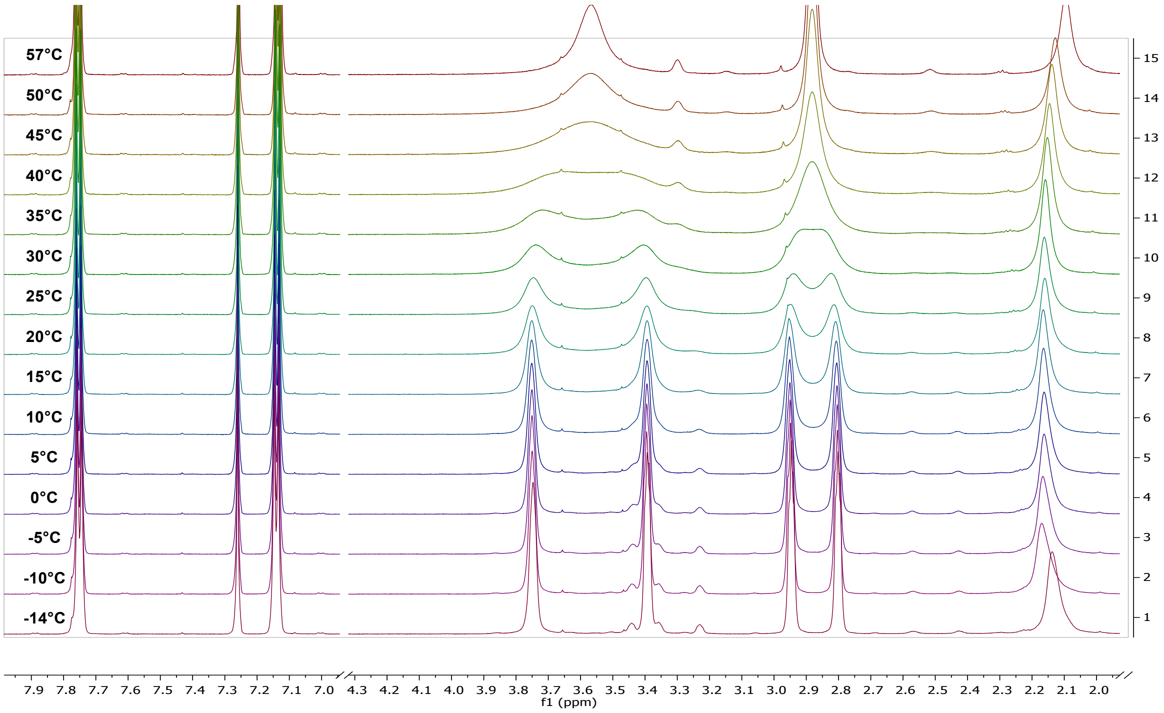

Figure S13. Temperature-depended ^1H NMR spectrum of compound **3g** measured in $CDCl_3$.

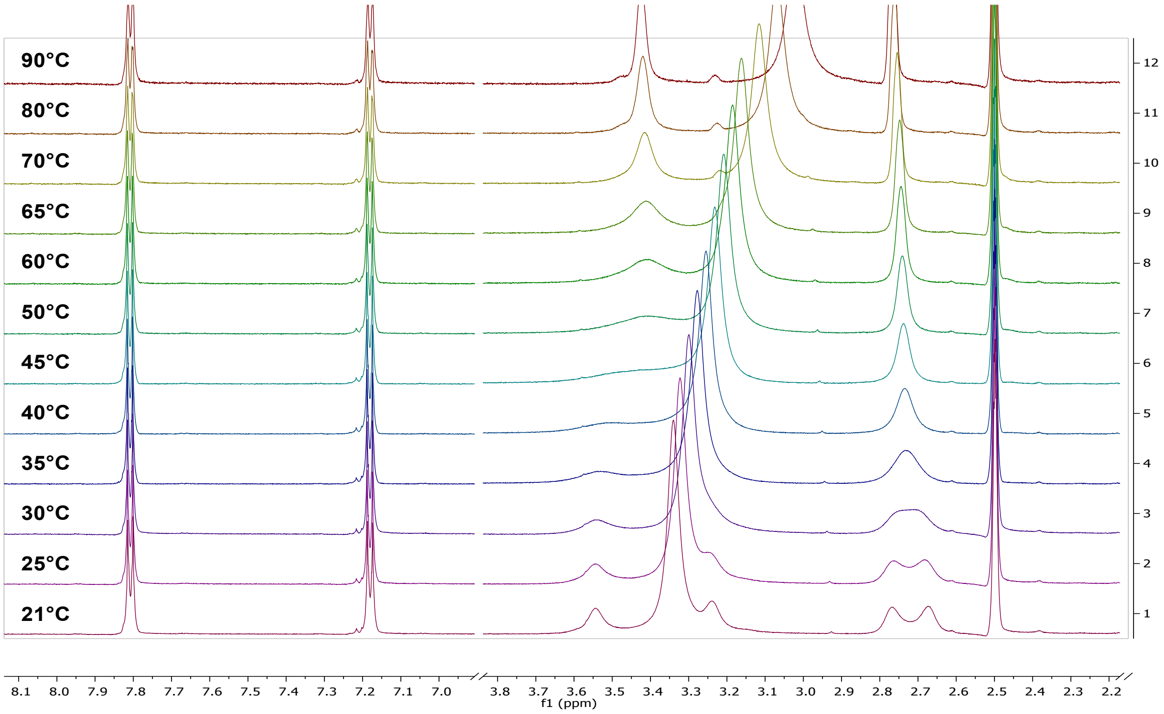

Figure S14. Temperature-depended ^1H NMR spectrum of compound **3g** measured in DMSO-d_6.

N-(4-Nitrobenzoyl)piperazine (**3h**)

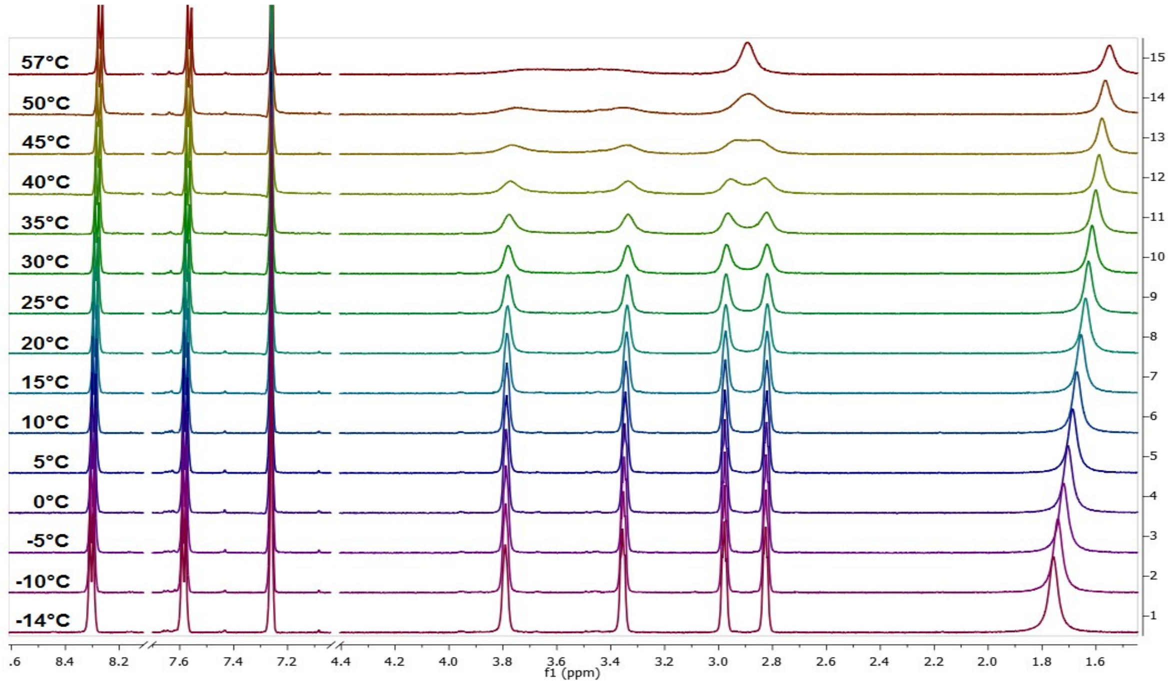

Figure S15. Temperature-depended ^{1}H NMR spectrum of compound **3h** measured in $CDCl_3$.

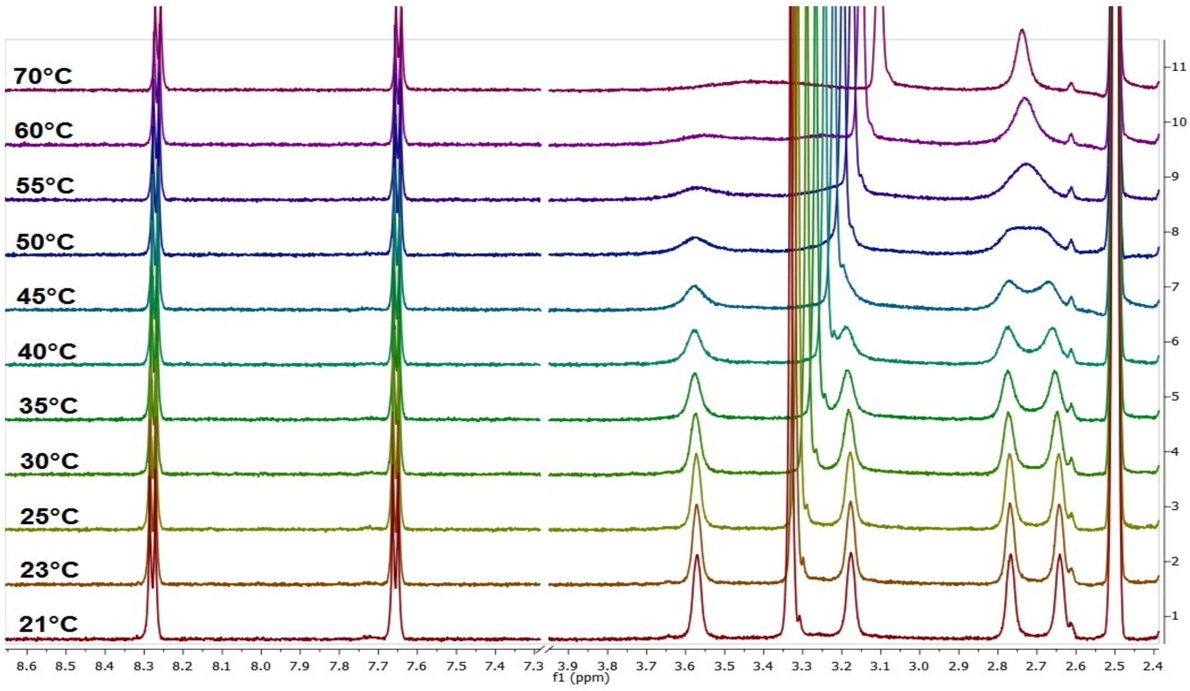

Figure S16. Temperature-depended ^{1}H NMR spectrum of compound **3h** measured in DMSO-d_6.

N-(3-Bromobenzoyl)piperazine (**3i**)

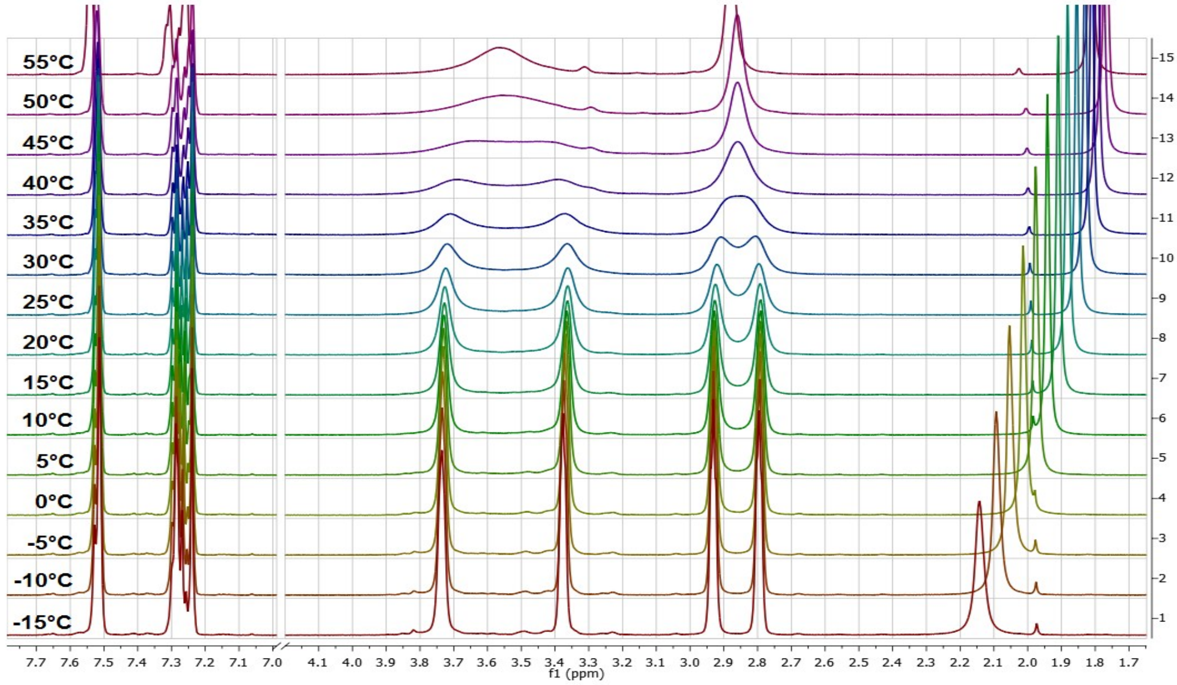

Figure S17. Temperature-depended ^{1}H NMR spectrum of compound **3i** measured in $CDCl_3$.

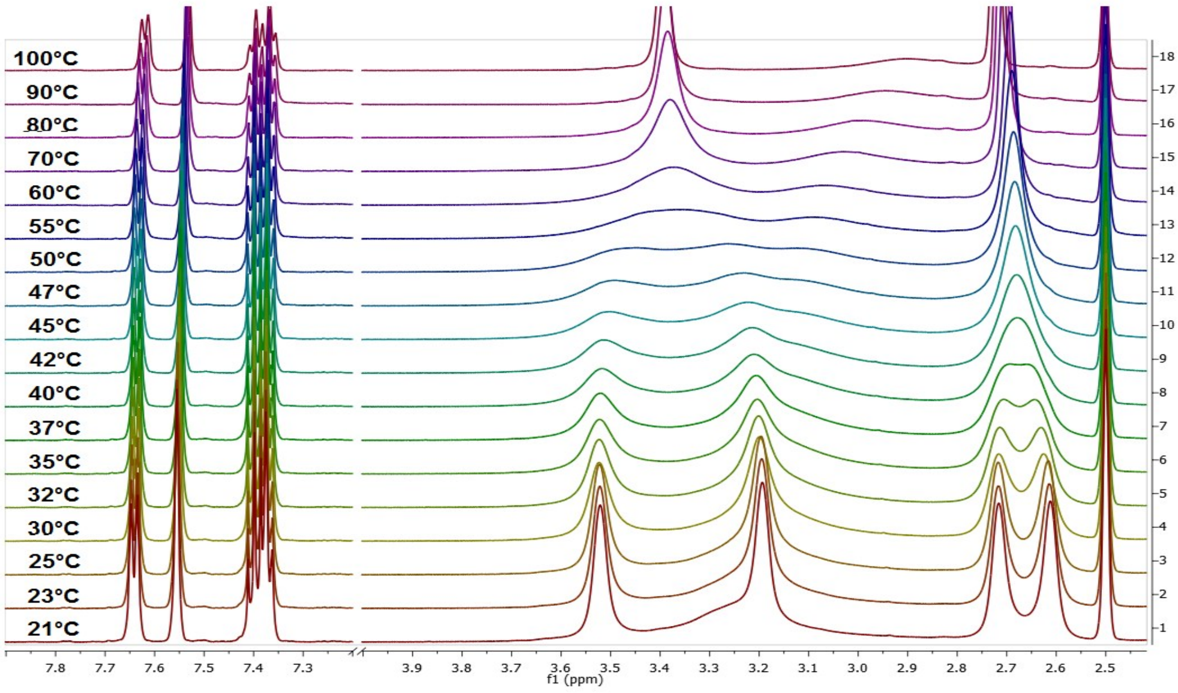

Figure 18. Temperature-depended ^{1}H NMR spectrum of compound **3i** measured in DMSO-d_6.

N,*N'*-Bisbenzoylpiperazine (**4a**)

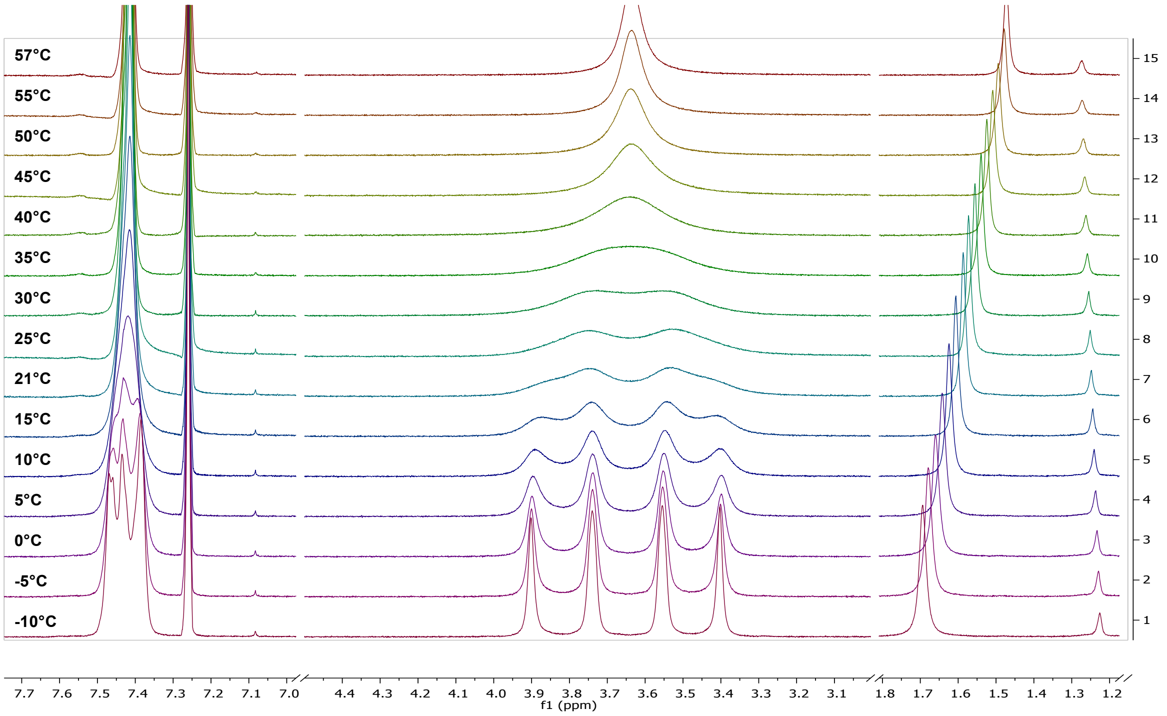

Figure S19. Temperature-depended ^{1}H NMR spectrum of compound **4a** measured in $CDCl_3$.

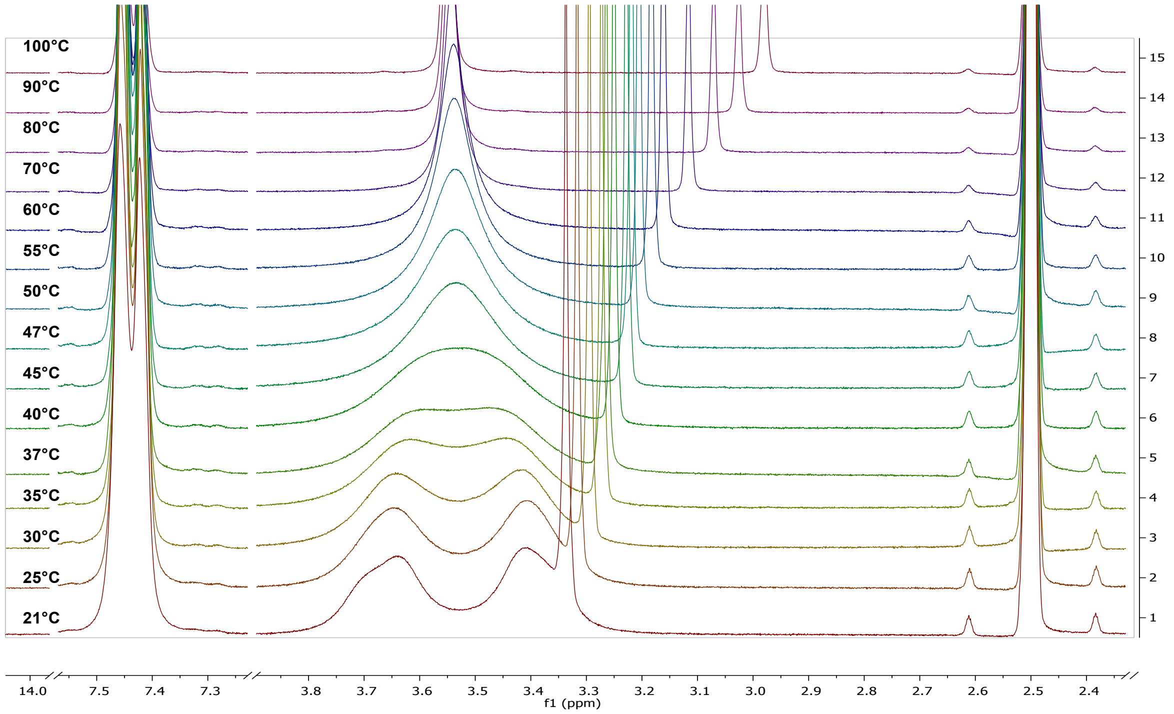

Figure S20. Temperature-depended ^{1}H NMR spectrum of compound **4a** measured in DMSO-d_6.

N,*N*'-Bis-(4-methylbenzoyl)piperazine (**4b**)

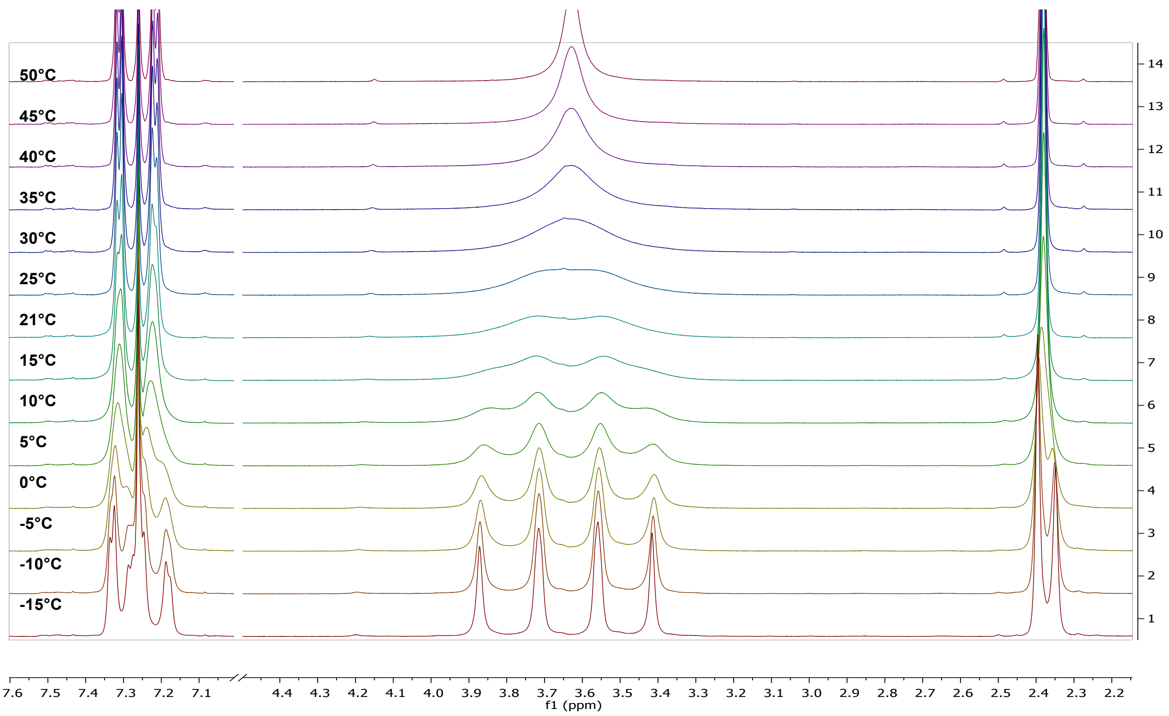

Figure S21. Temperature-depended ^{1}H NMR spectrum of compound **4b** measured in $CDCl_3$.

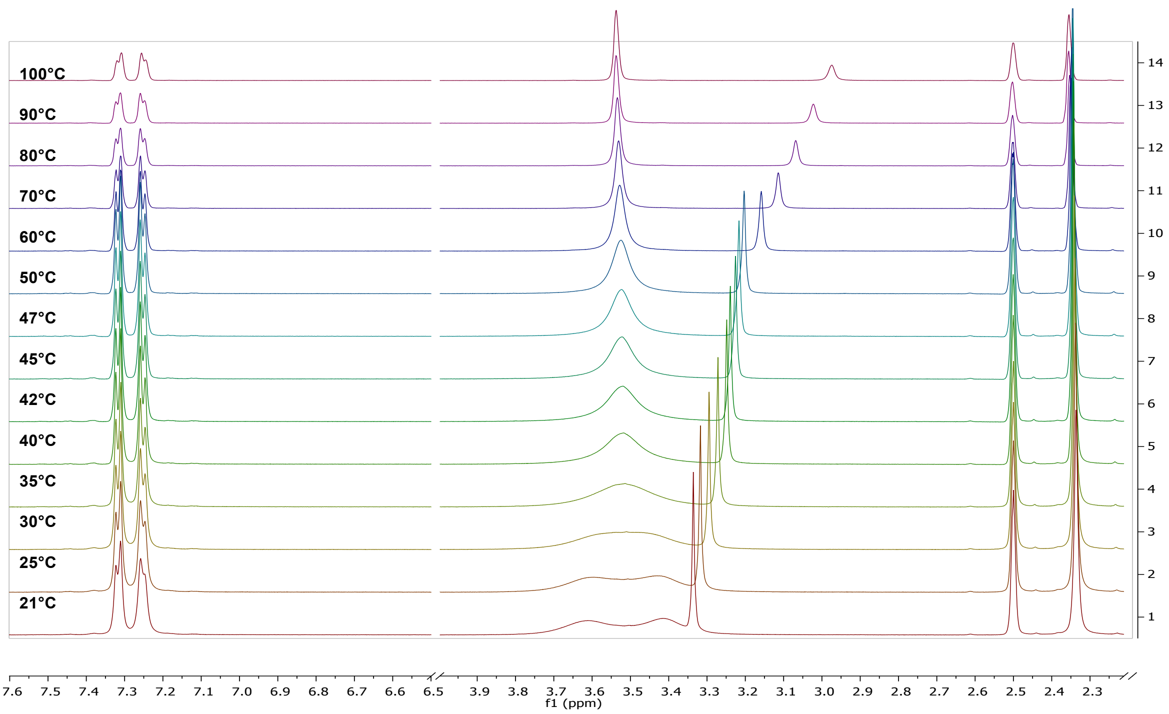

Figure S22. Temperature-depended ^{1}H NMR spectrum of compound **4b** measured in DMSO-d_6.

N,*N*‘-Bis-(4-methoxybenzoyl)piperazine (**4c**)

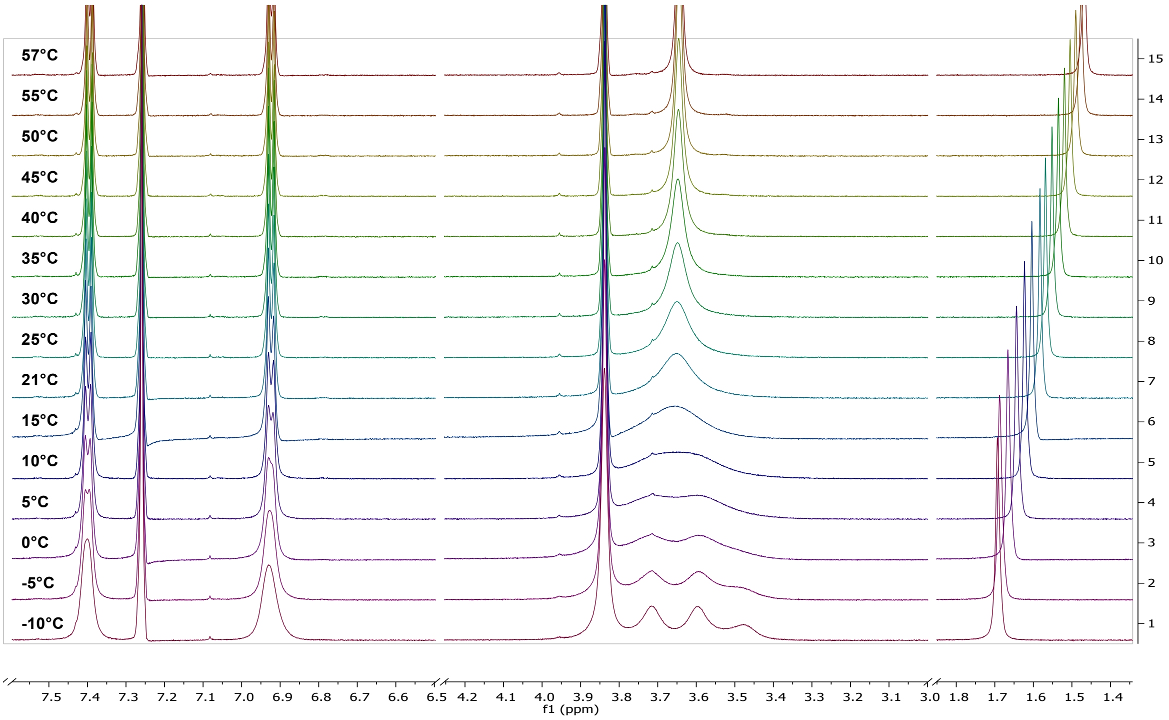

Figure S23. Temperature-depended ^{1}H NMR spectrum of compound **4c** measured in $CDCl_3$.

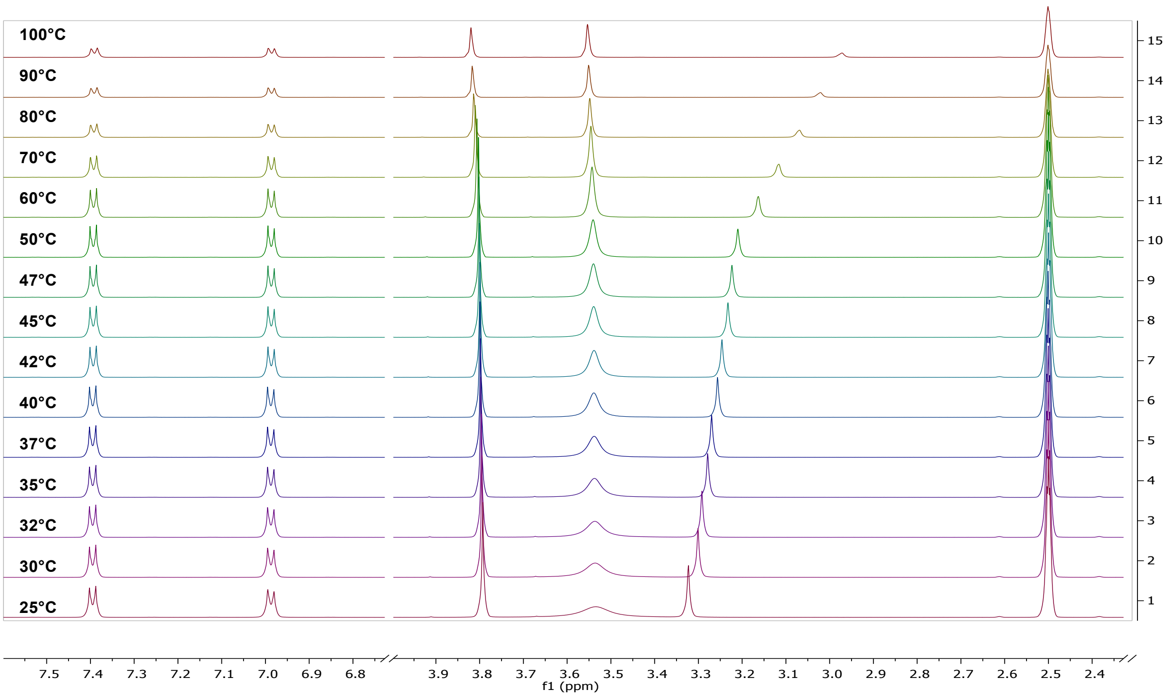

Figure S24. Temperature-depended ^{1}H NMR spectrum of compound **4c** measured in DMSO-d_6.

N,*N'*-Bis-(4-fluorobenzoyl)piperazine (**4d**)

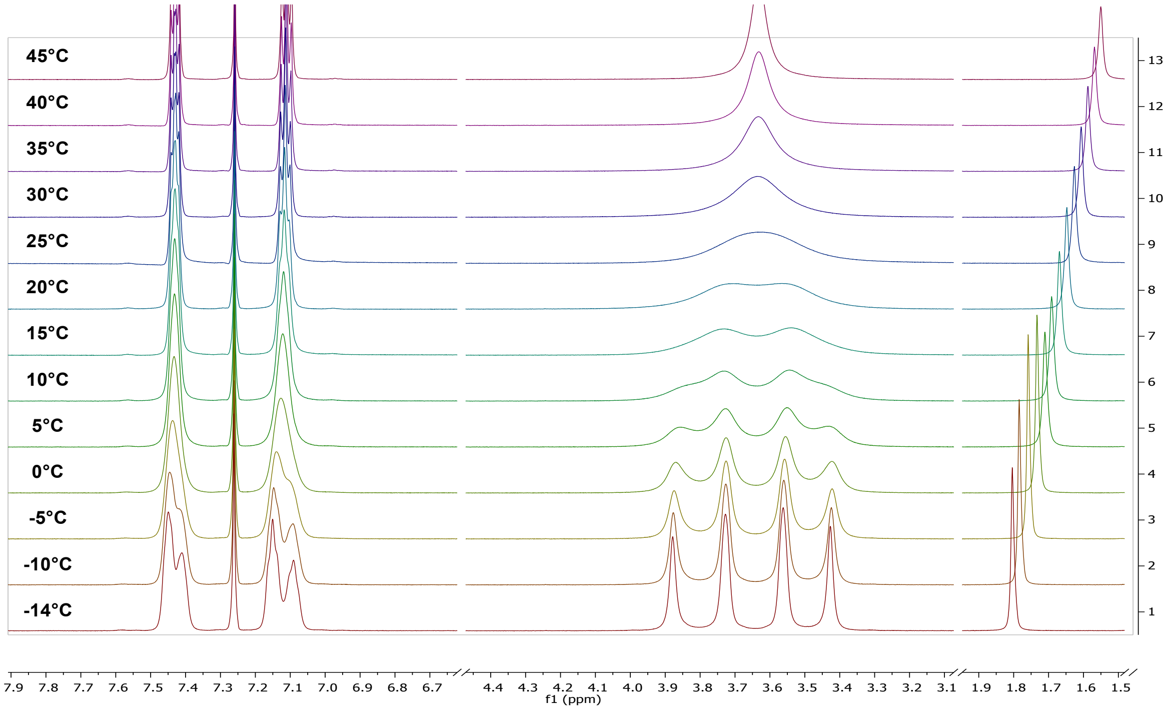

Figure S25. Temperature-depended ^{1}H NMR spectrum of compound **4d** measured in $CDCl_3$.

N,*N*‘-Bis-(4-chlorobenzoyl)piperazine (**4e**)

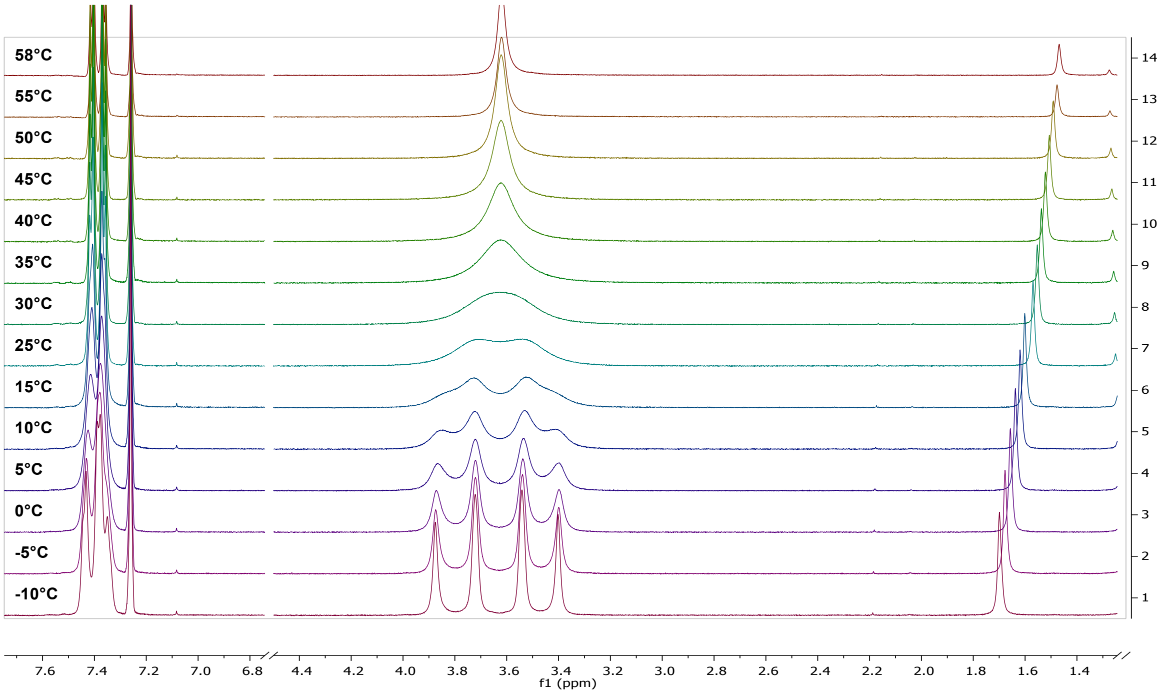

Figure S26. Temperature-depended ^{1}H NMR spectrum of compound **4e** measured in $CDCl_3$.

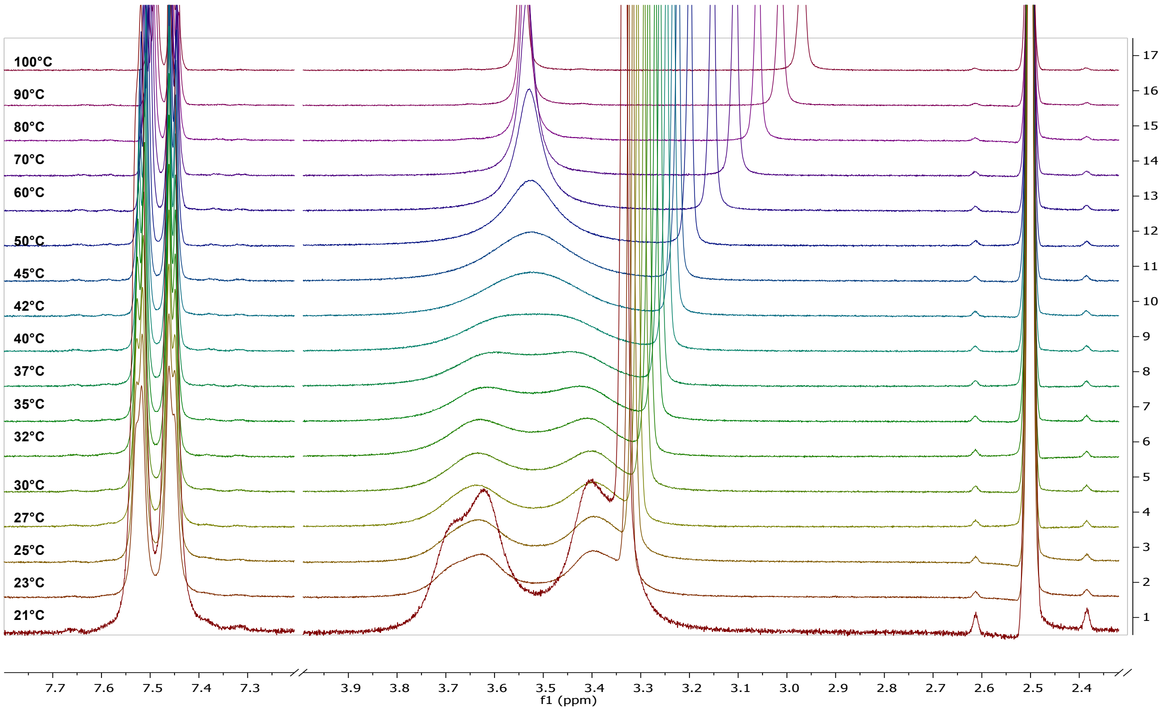

Figure S29. Temperature-depended ^{1}H NMR spectrum of compound **4e** measured in DMSO-d_6.

N,*N'*-Bis-(4-bromobenzoyl)piperazine (**4f**)

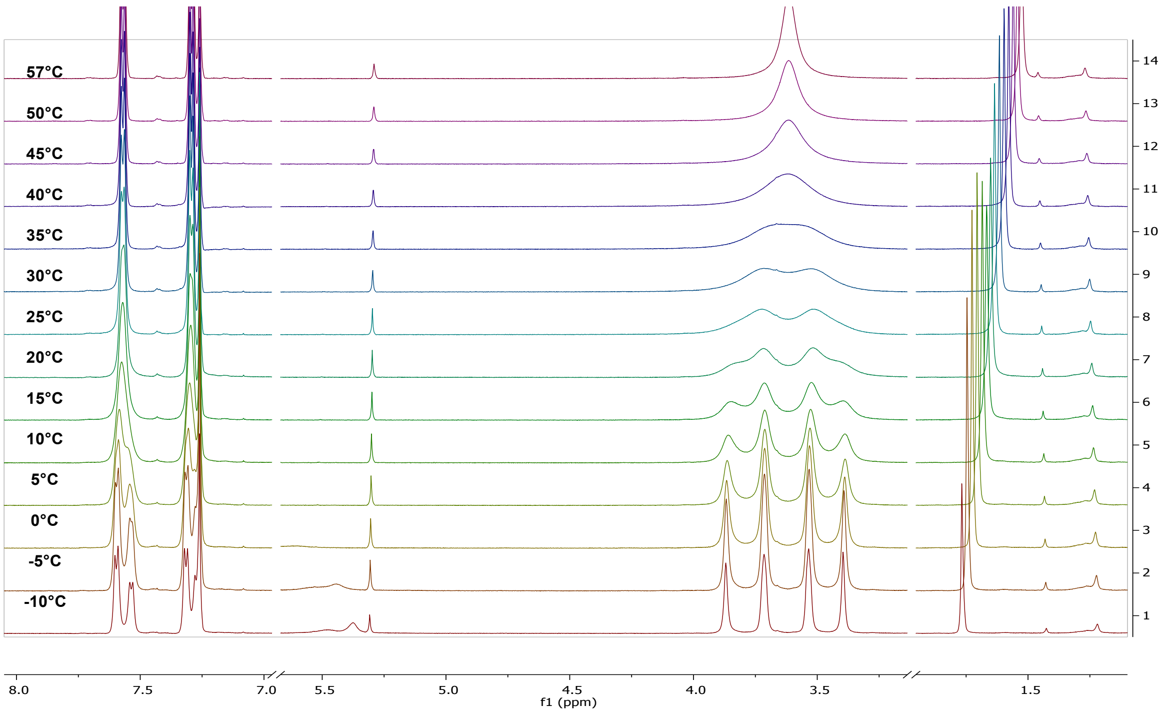

Figure S28. Temperature-depended ^{1}H NMR spectrum of compound **4f** measured in $CDCl_3$.

N,*N*‘-Bis-(4-iodobenzoyl)piperazine (**4g**)

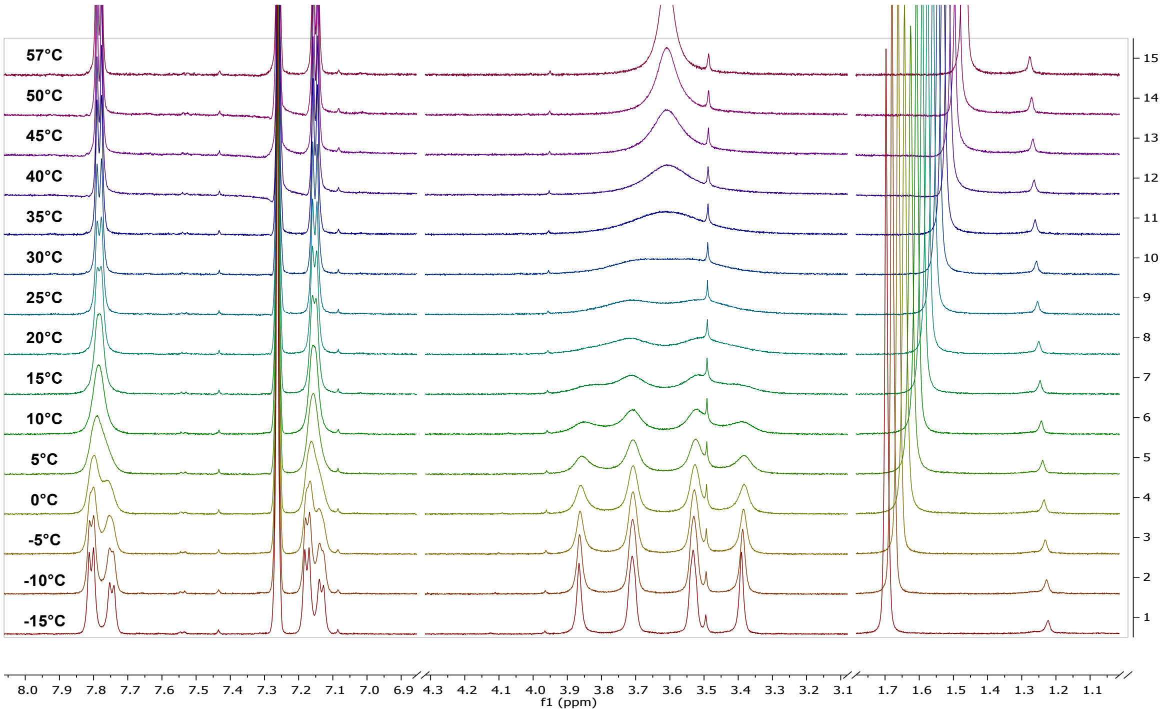

Figure S30. Temperature-depended ^{1}H NMR spectrum of compound **4g** measured in $CDCl_3$.

N-Benzoyl-*N′*-(4-nitrophenyl)piperazine (**6a**)

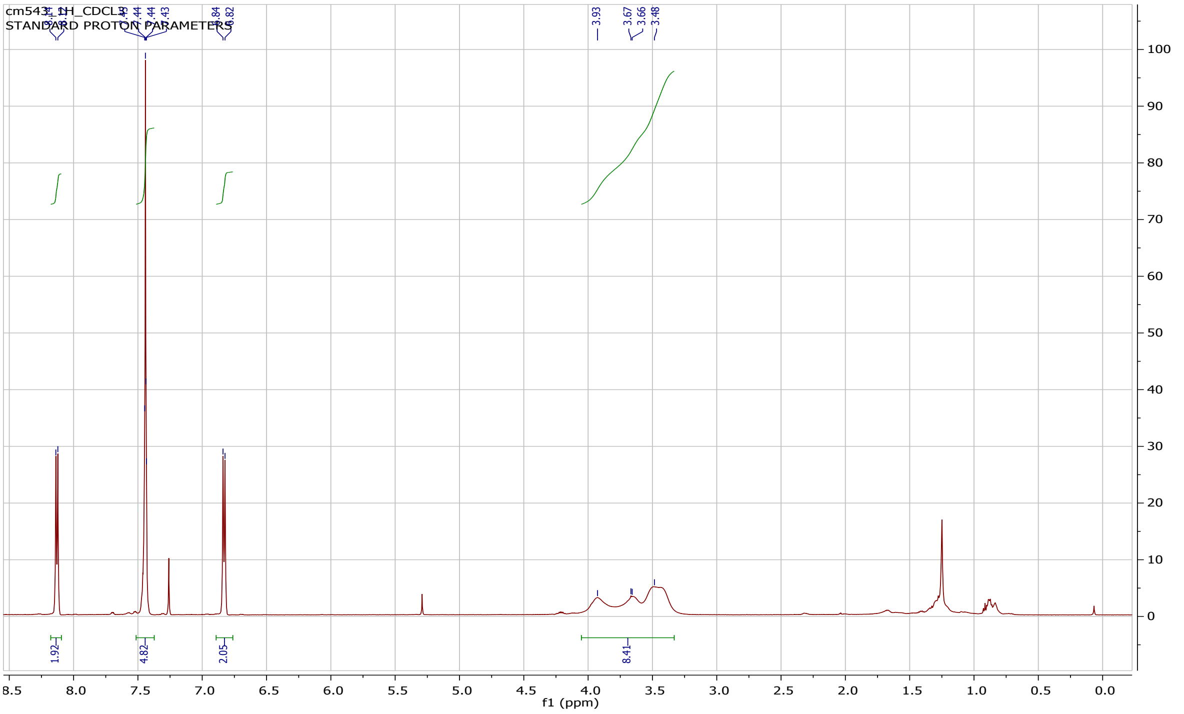

Figure S31. ^{1}H NMR spectrum of compound **6a** measured in $CDCl_3$.

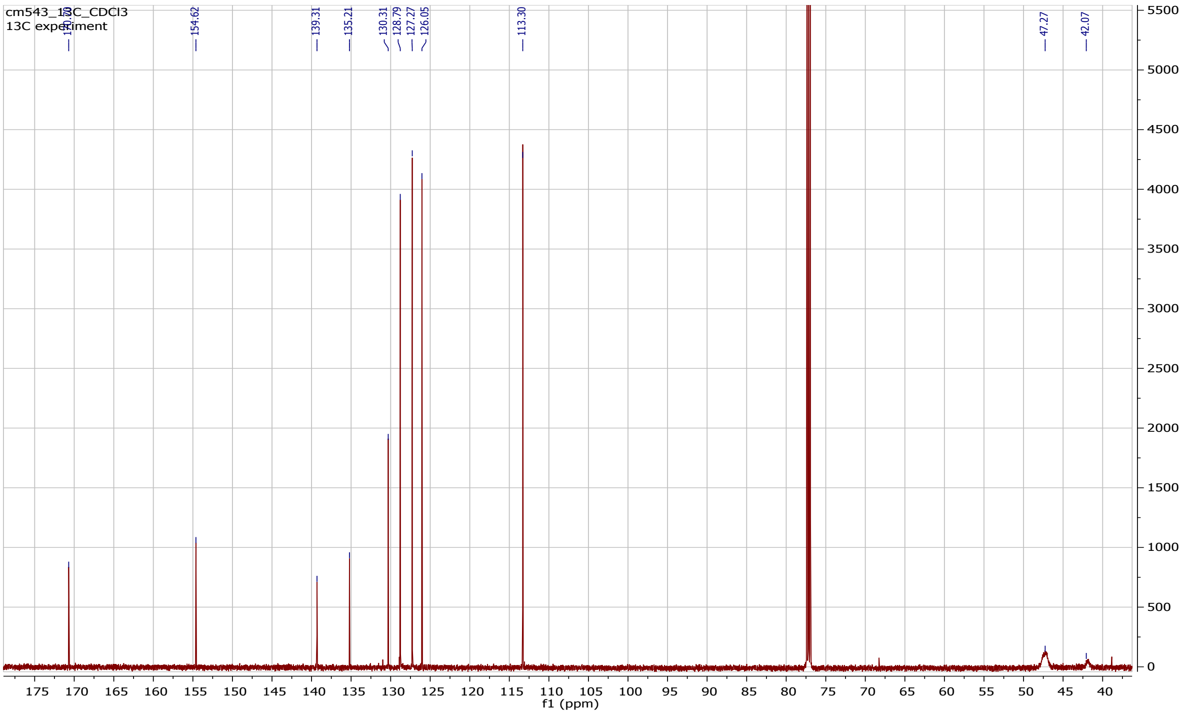

Figure S32. ^{13}C NMR spectrum of compound **6a** measured in $CDCl_3$.

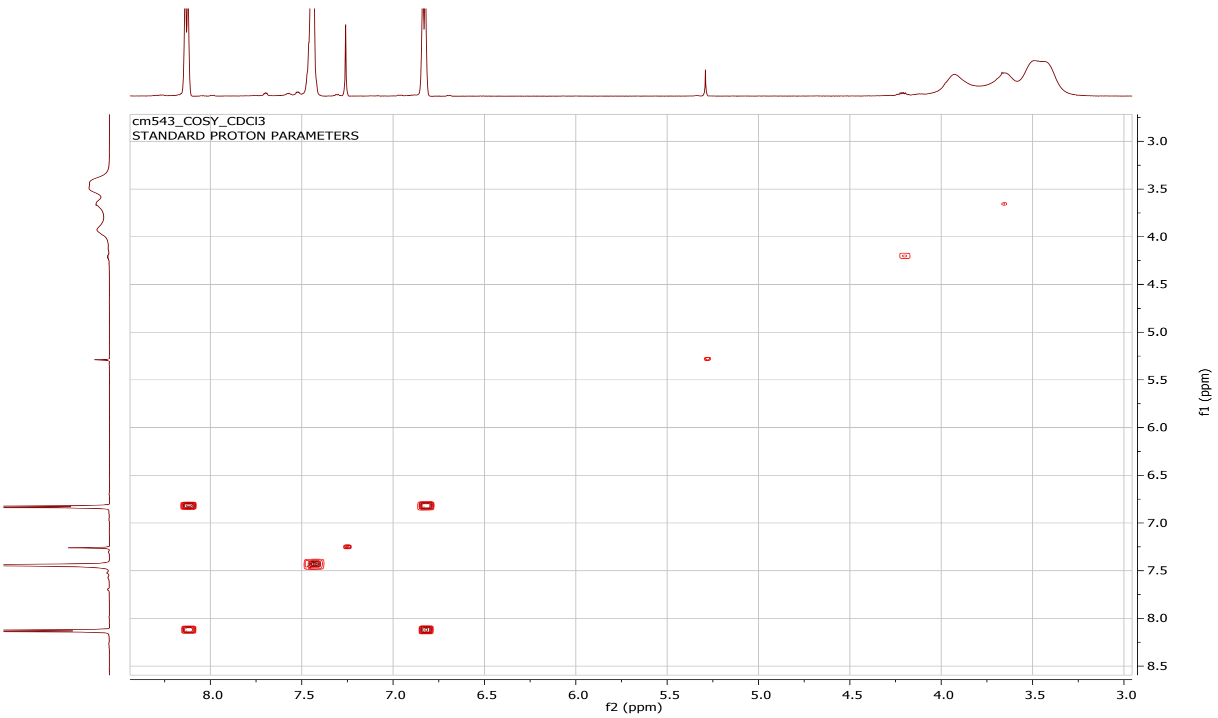

Figure S33. H-H-COSY spectrum of compound **6a** measured in $CDCl_3$.

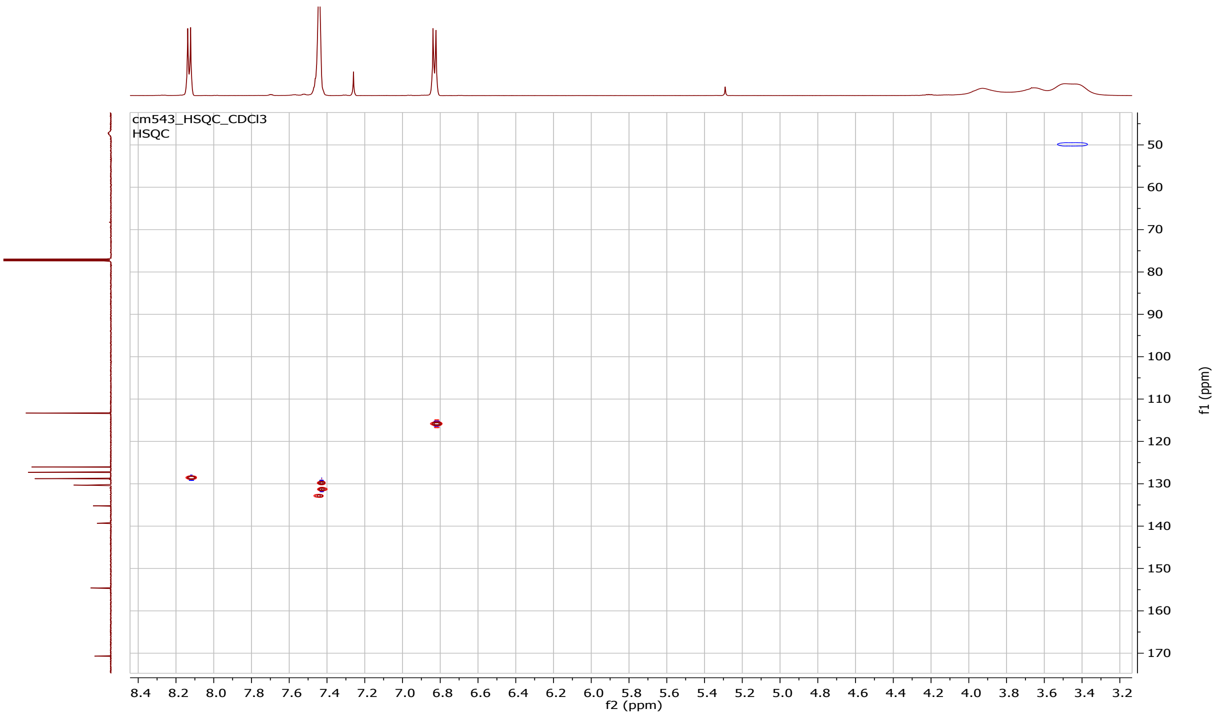

Figure S34. HSQC spectrum of compound **6a** measured in $CDCl_3$.

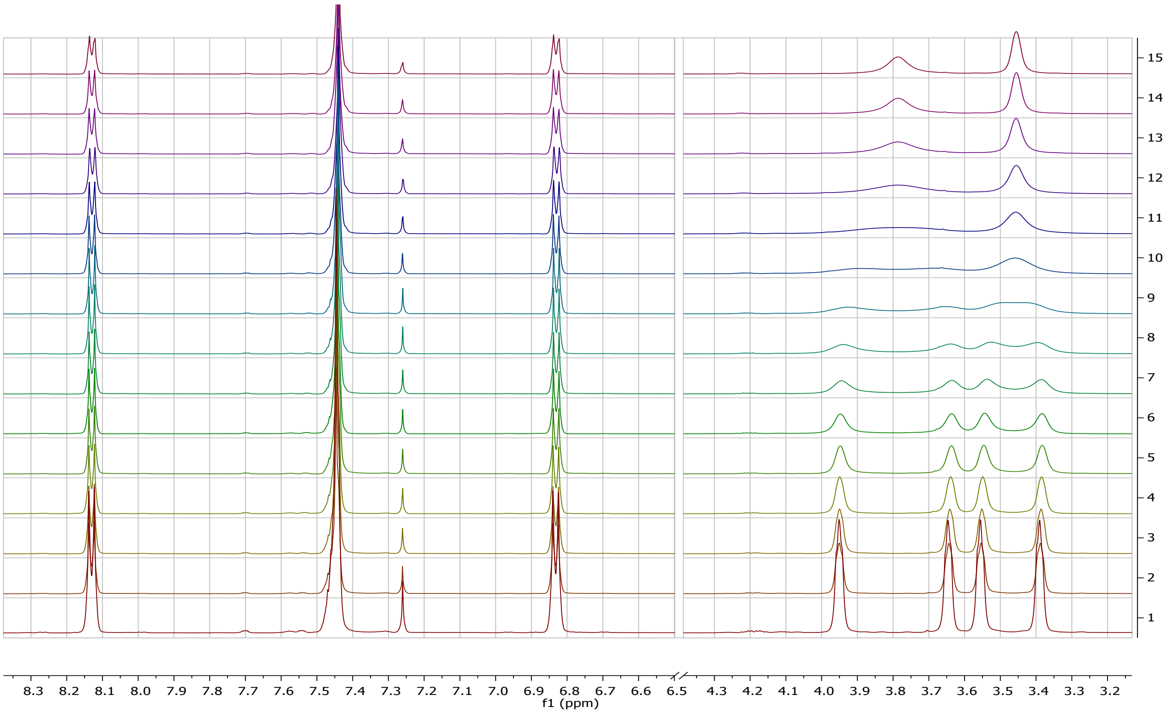

Figure S35. Temperature-depended ^{1}H NMR spectrum of compound **6a** measured in $CDCl_3$.

N-(4-Methylbenzoyl)-*N'*-(4-nitrophenyl)piperazine (**6b**)

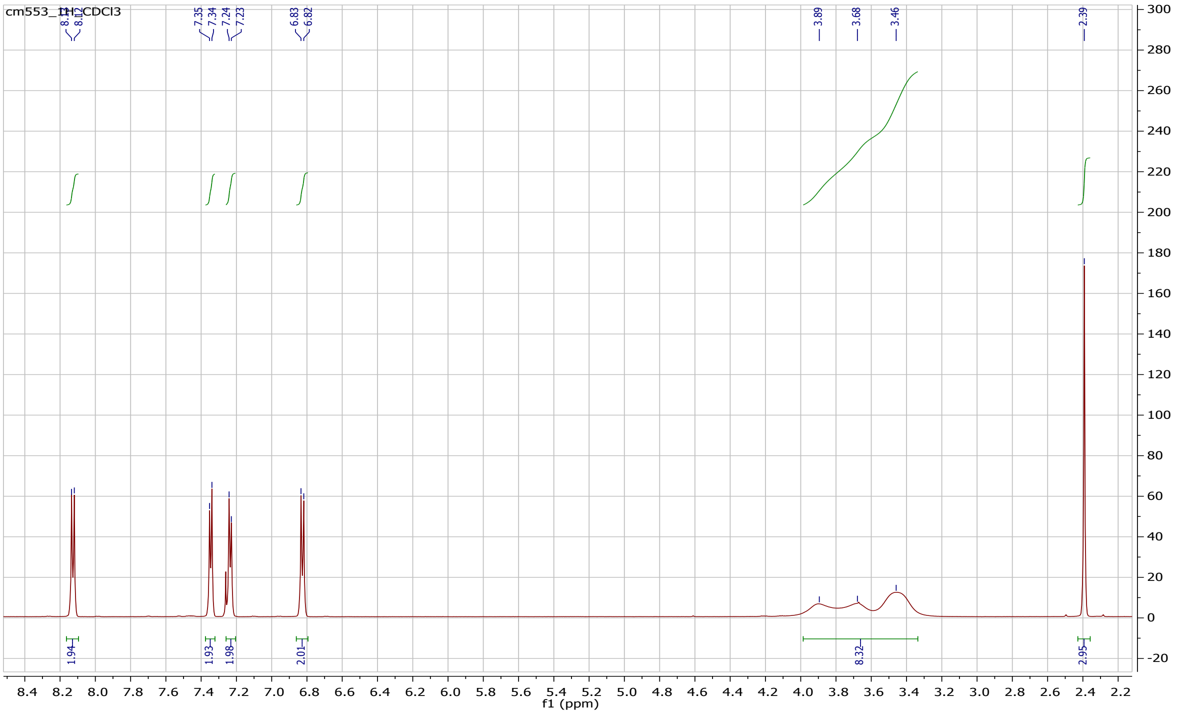

Figure S36. ^{1}H NMR spectrum of compound **6b** measured in $CDCl_3$.

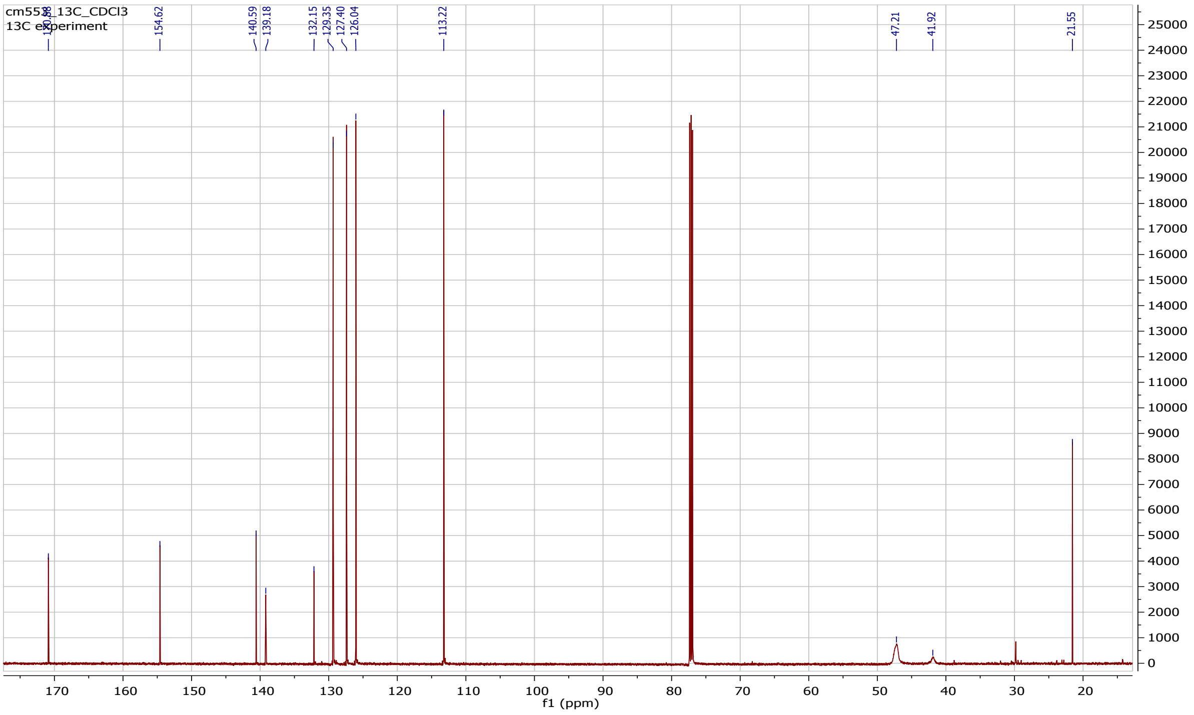

Figure S37. ^{13}C NMR spectrum of compound **6b** measured in $CDCl_3$.

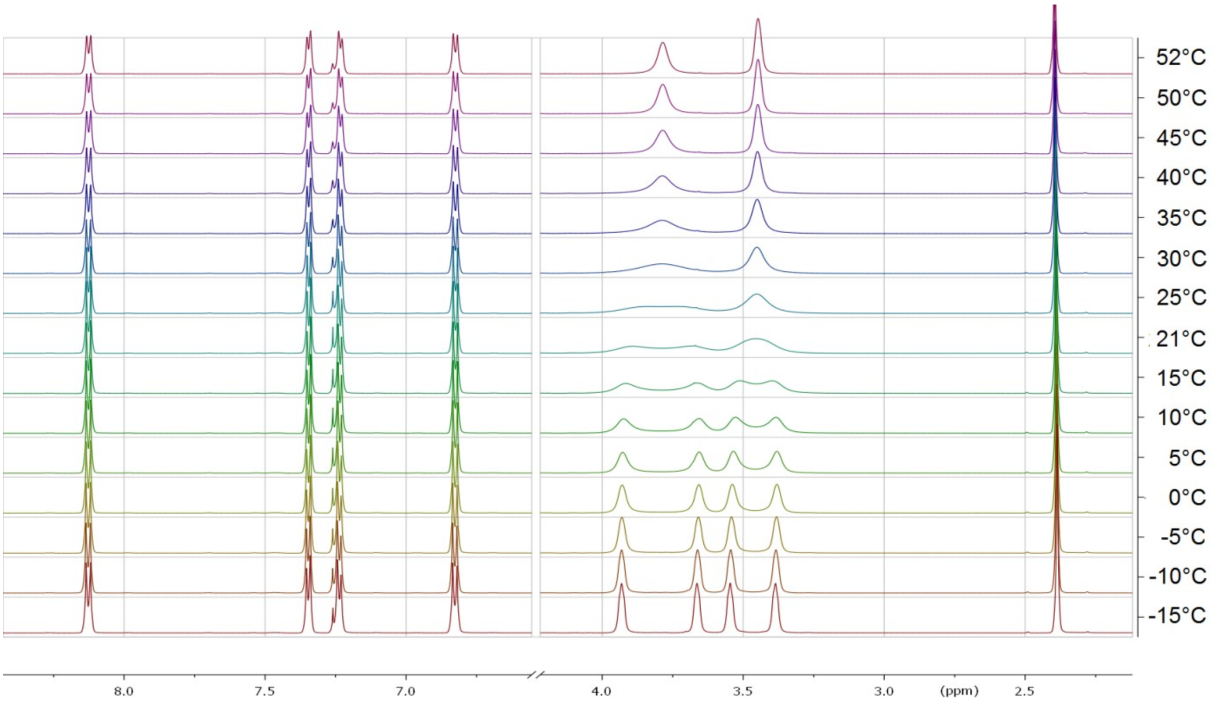

Figure S38. Temperature-depended ^{1}H NMR spectrum of compound **6b** measured in $CDCl_3$.

N-(4-Methoxybenzoyl)-*N'*-(4-nitrophenyl)piperazine (**6c**)

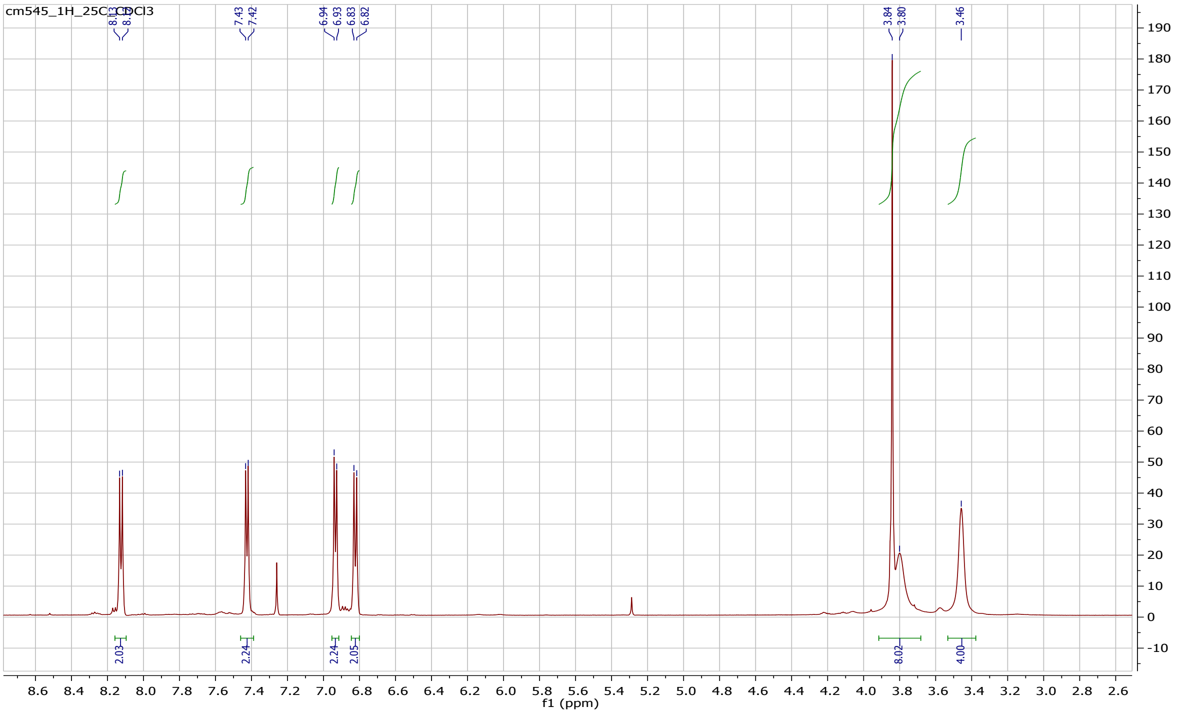

Figure S39. ^{1}H NMR spectrum of compound **6c** measured in $CDCl_3$.

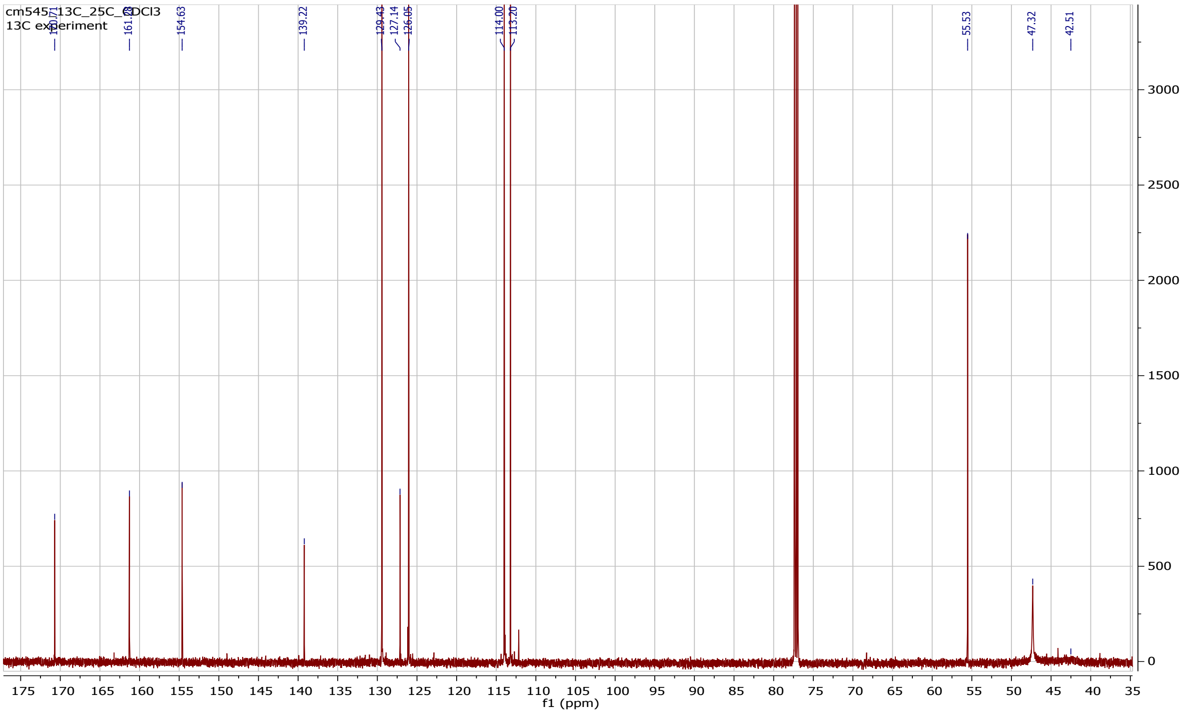

Figure S40. ^{13}C NMR spectrum of compound **6c** measured in $CDCl_3$.

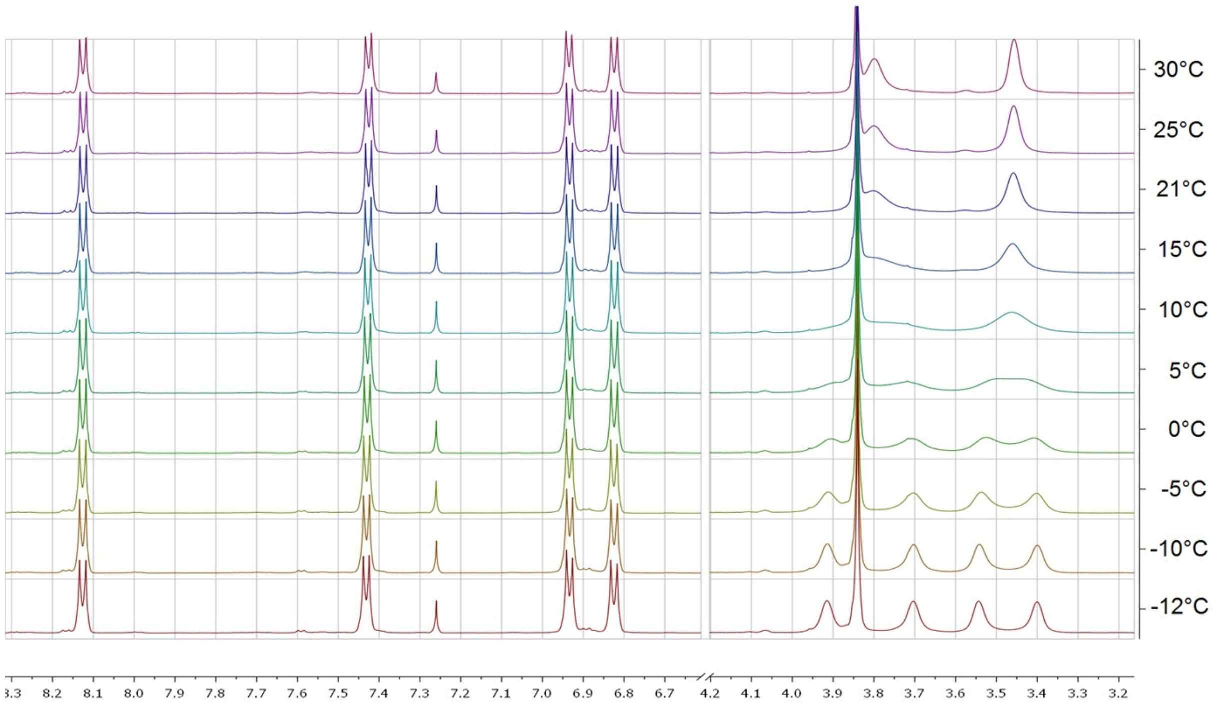

Figure S41. Temperature-depended ^{1}H NMR spectrum of compound **6c** measured in $CDCl_3$.

N-(4-Fluorobenzoyl)-*N'*-(4-nitrophenyl)piperazine (**6d**)

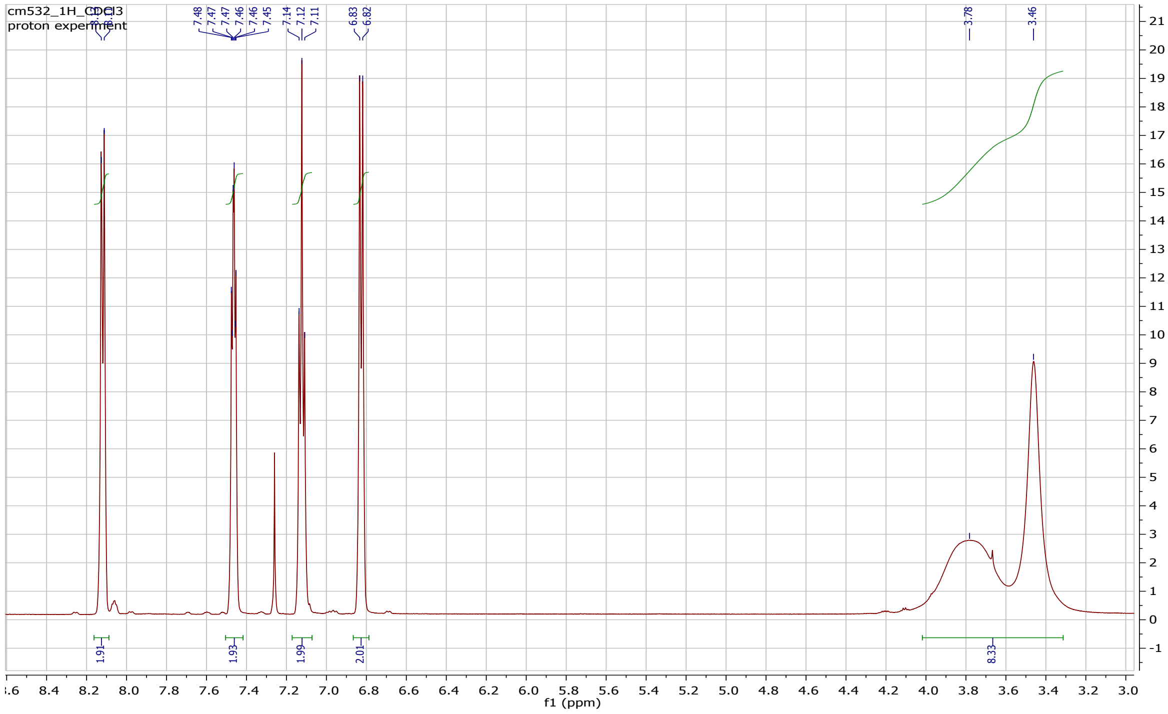

Figure S42. ^{1}H NMR spectrum of compound **6d** measured in $CDCl_3$.

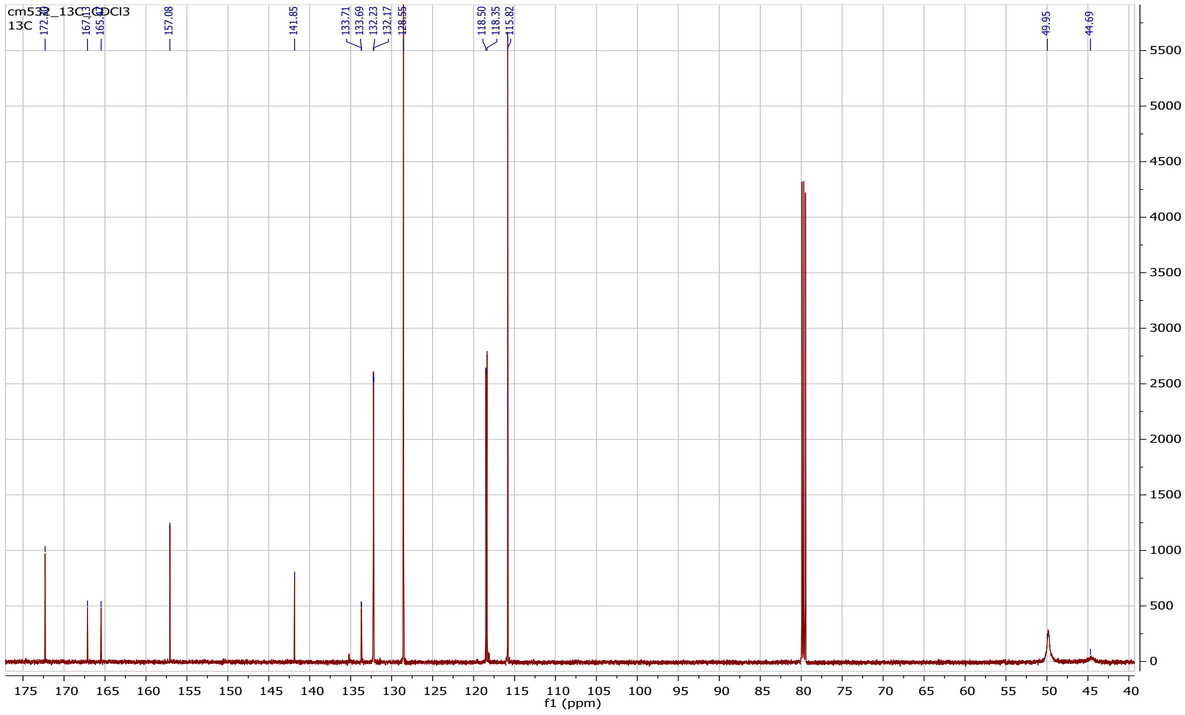

Figure S43. ^{13}C NMR spectrum of compound **6d** measured in $CDCl_3$.

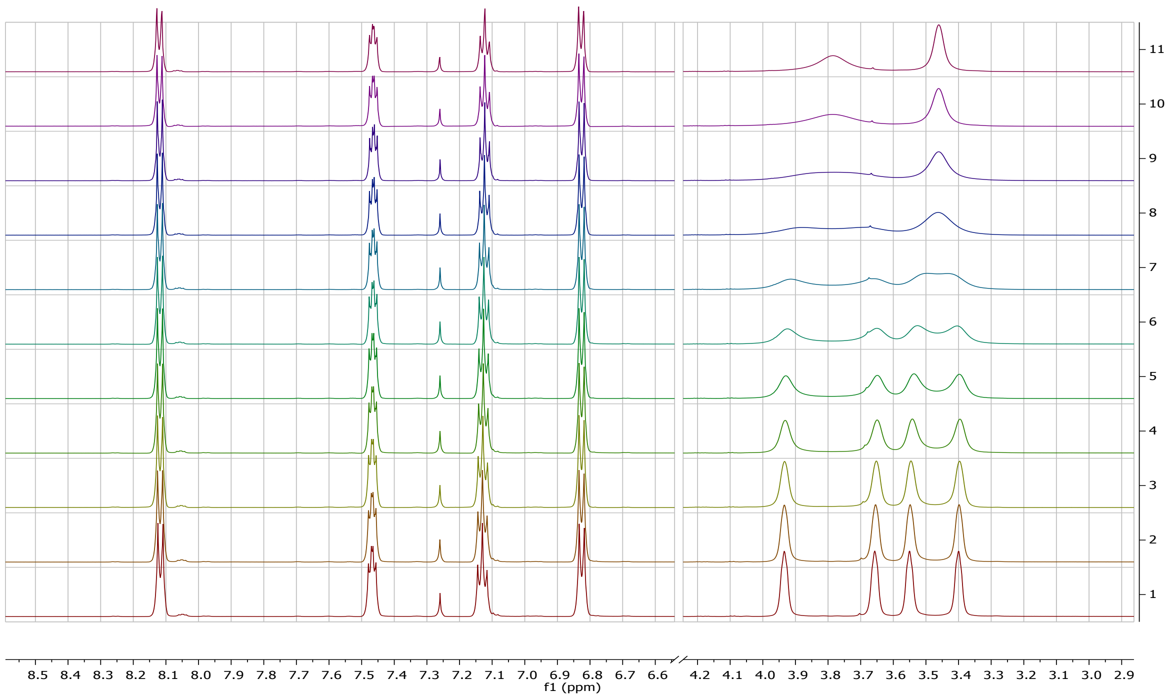

Figure S44. Temperature-depended ^{1}H NMR spectrum of compound **6d** measured in $CDCl_3$.

N-(4-Bromobenzoyl)-*N'*-(4-nitrophenyl)piperazine (**6f**)

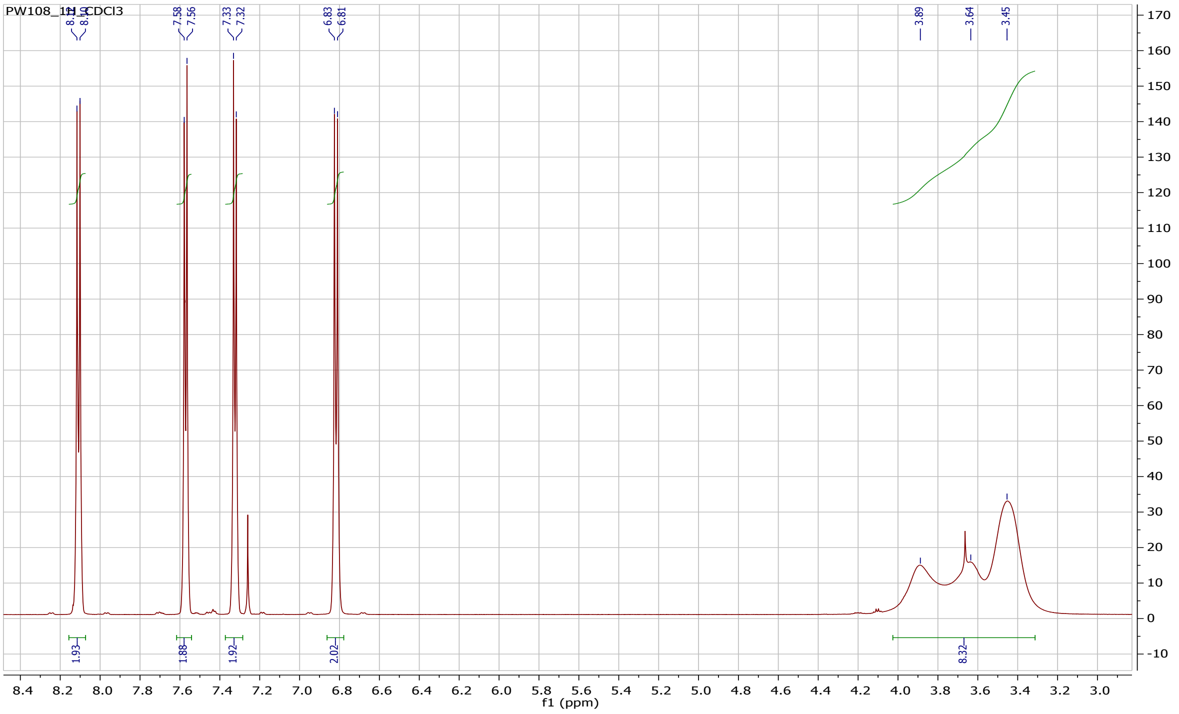

Figure S45. ^{1}H NMR spectrum of compound **6f** measured in $CDCl_3$.

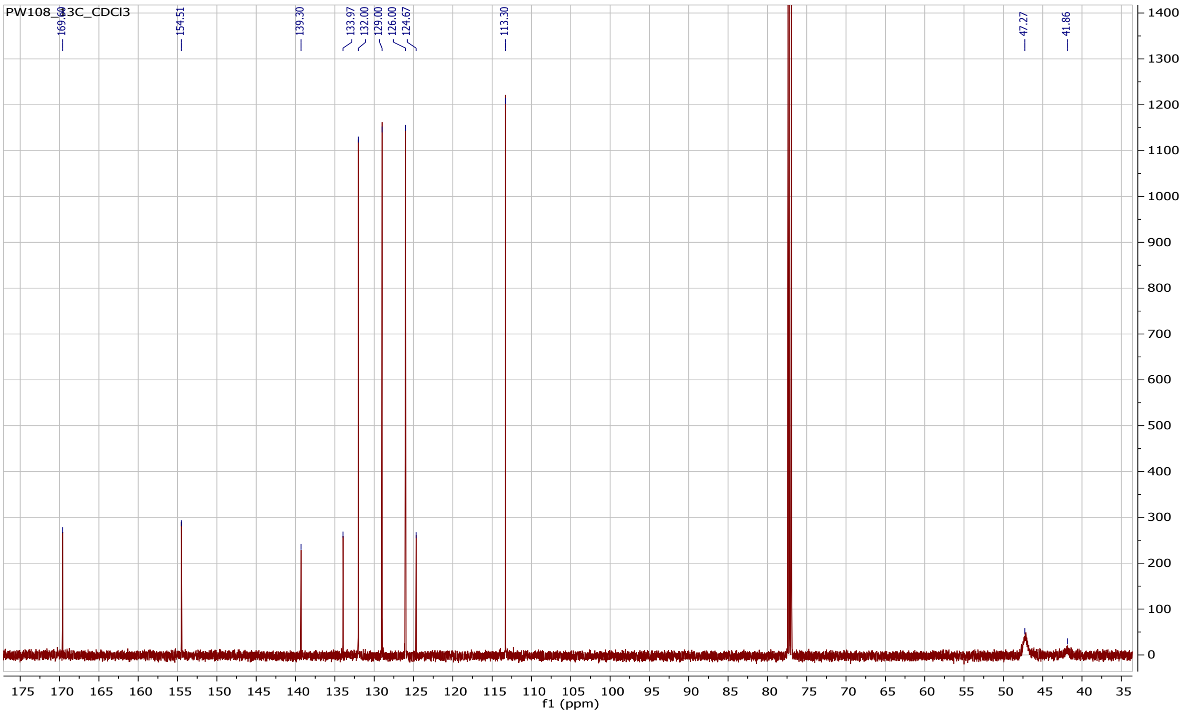

Figure S46. ^{13}C NMR spectrum of compound **6f** measured in $CDCl_3$.

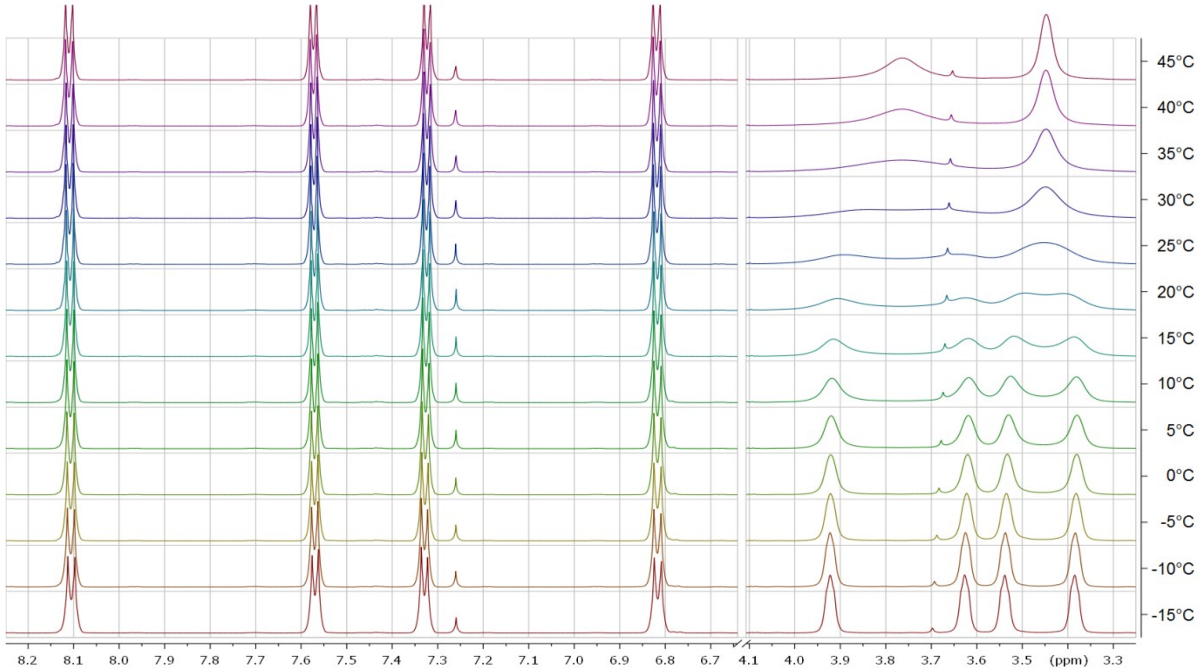

Figure S47. Temperature-depended ^{1}H NMR spectrum of compound **6f** measured in $CDCl_3$.

N-(4-Nitrobenzoyl)-*N'*-(4-nitrophenyl)piperazine (**6h**)

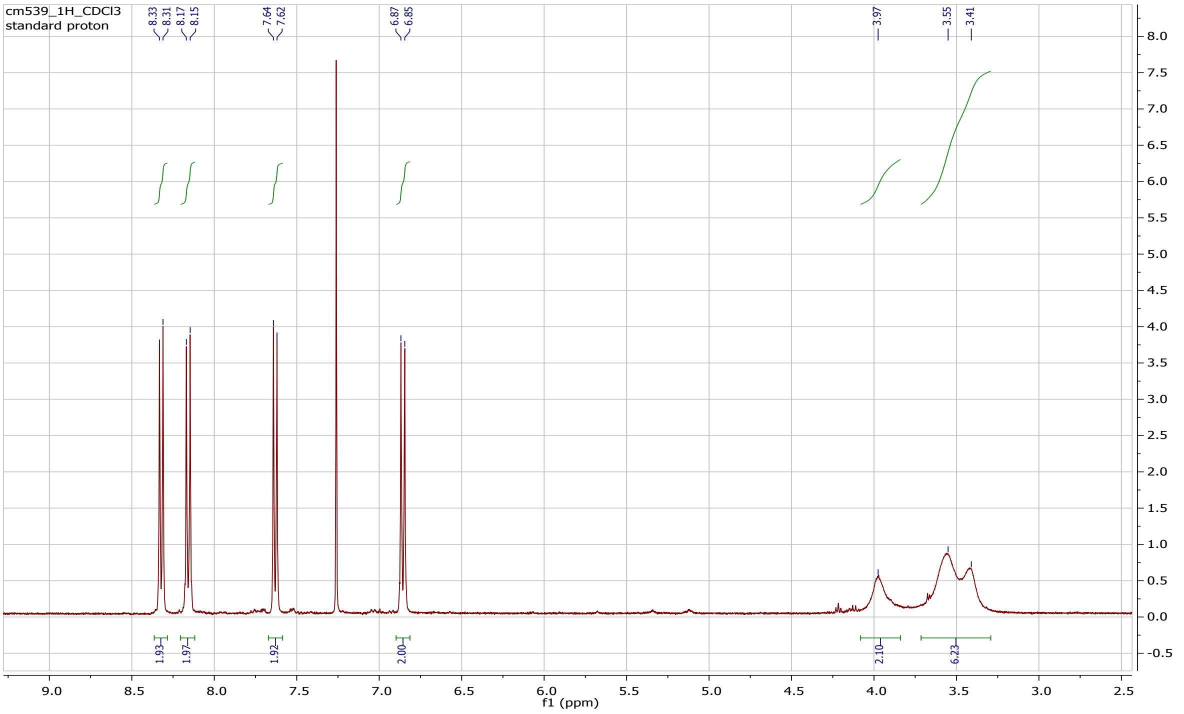

Figure S48. ^{1}H NMR spectrum of compound **6h** measured in $CDCl_3$.

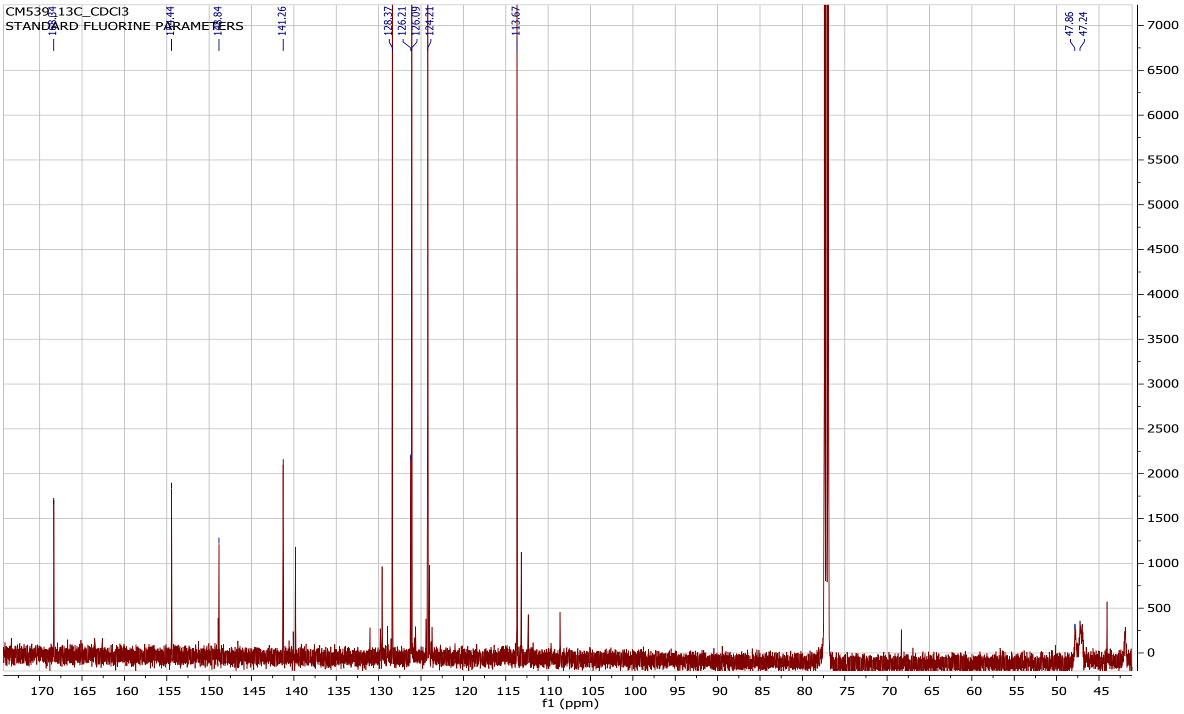

Figure S49. ^{13}C NMR spectrum of compound **6h** measured in $CDCl_3$.

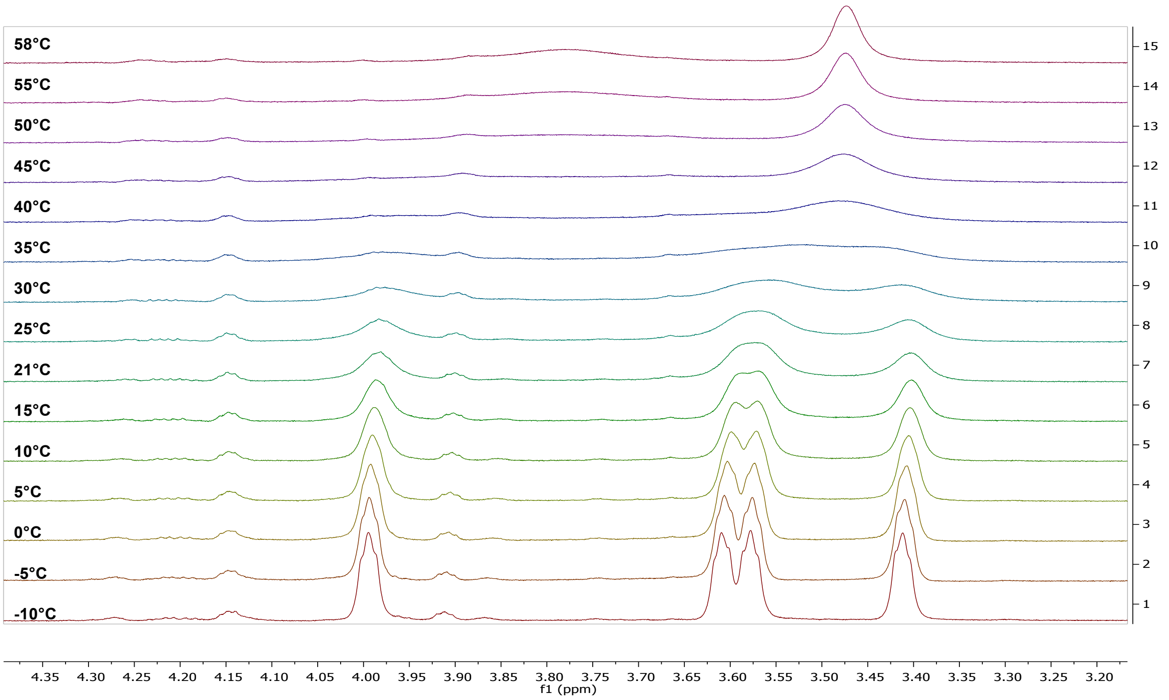

Figure S50. Temperature-depended ^{1}H NMR spectrum of compound **6h** measured in $CDCl_3$.

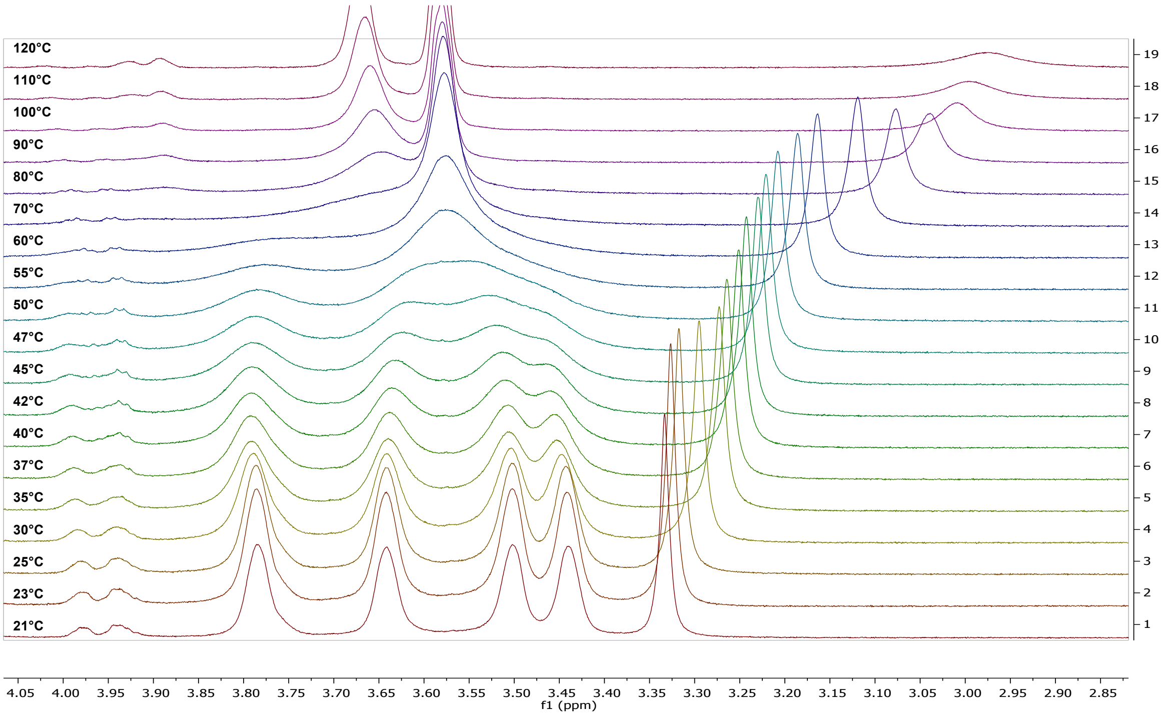

Figure S51. Temperature-depended ^{1}H NMR spectrum of compound **6h** measured in DMSO-d_6.

N-(3-Bromobenzoyl)-*N'*-(4-nitrophenyl)piperazine (**6i**)

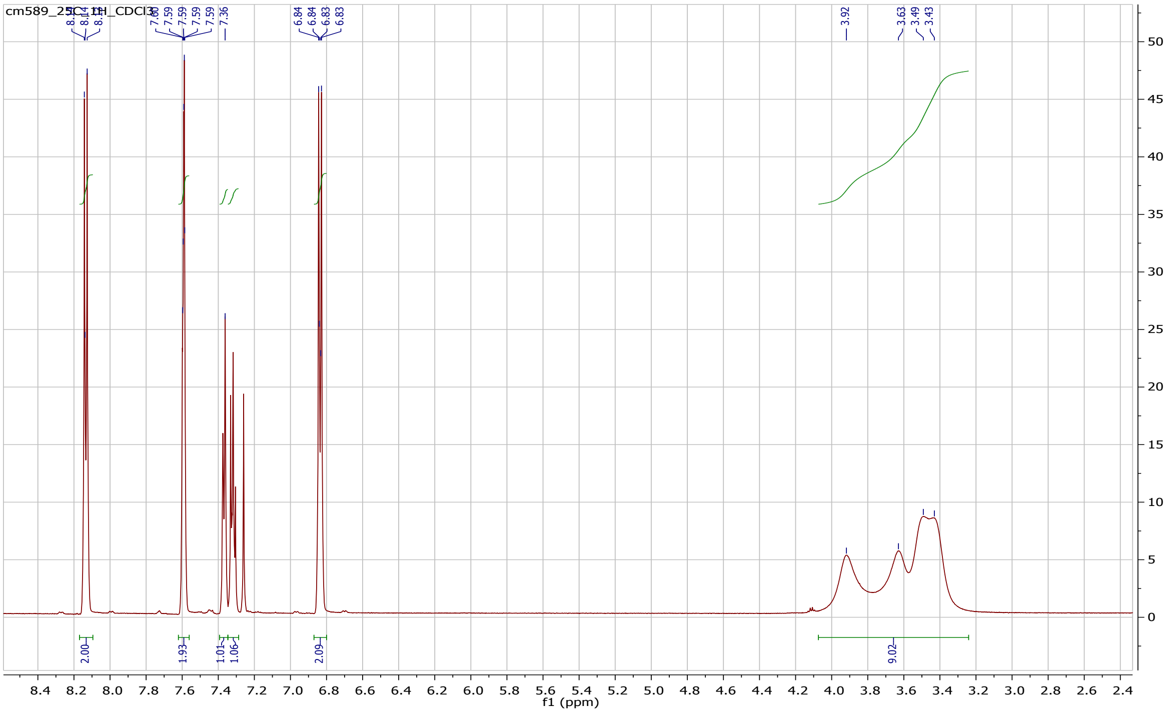

Figure S52. ^{1}H NMR spectrum of compound **6i** measured in $CDCl_3$.

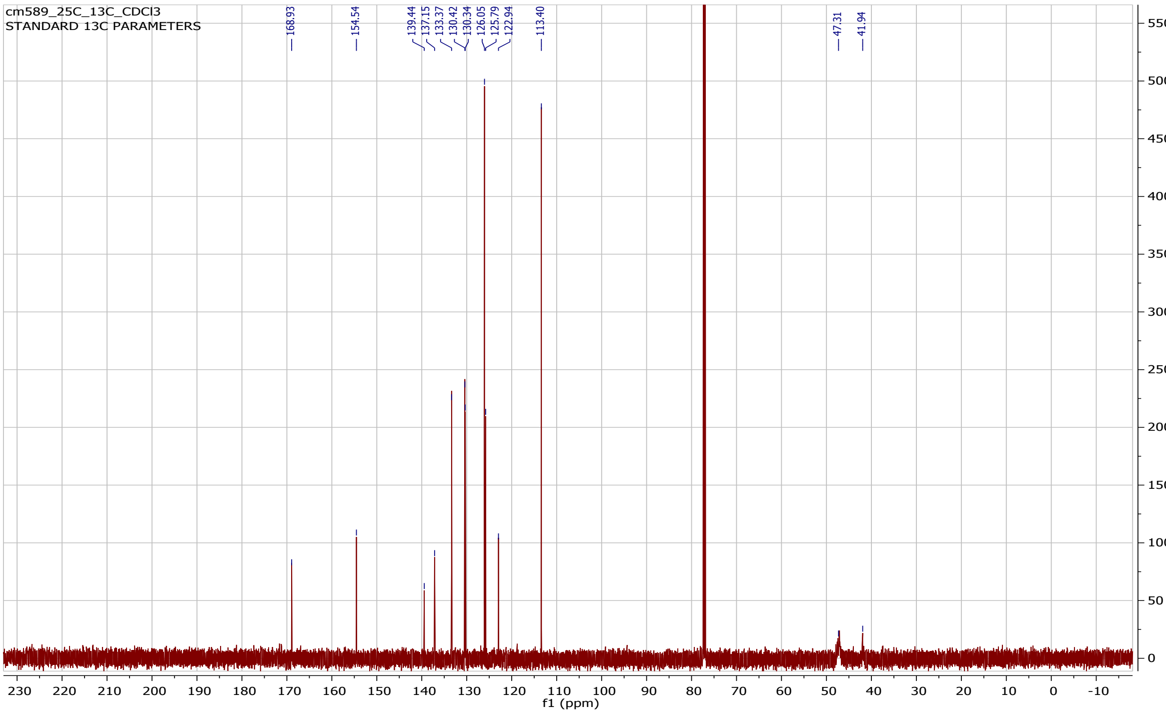

Figure S53. ^{13}C NMR spectrum of compound **6i** measured in $CDCl_3$.

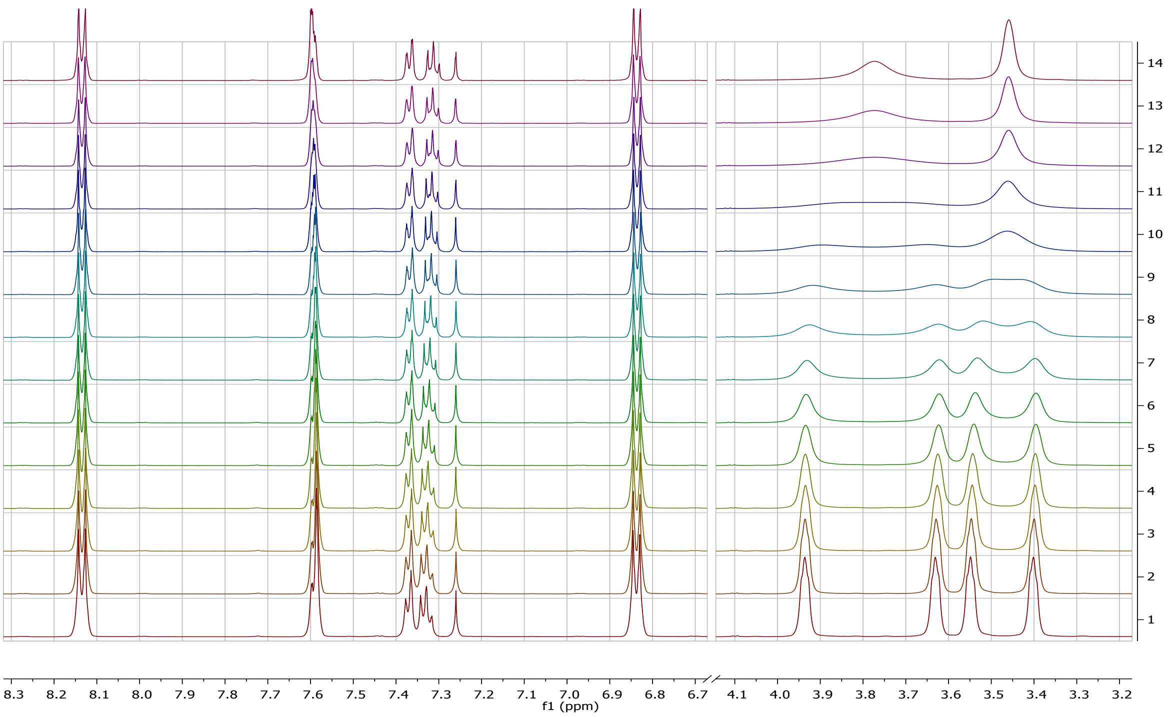

Figure S54. Temperature-depended ^{1}H NMR spectrum of compound **6i** measured in $CDCl_3$.

N-(2-Bromobenzoyl)-*N'*-(4-nitrophenyl)piperazine (**6j**)

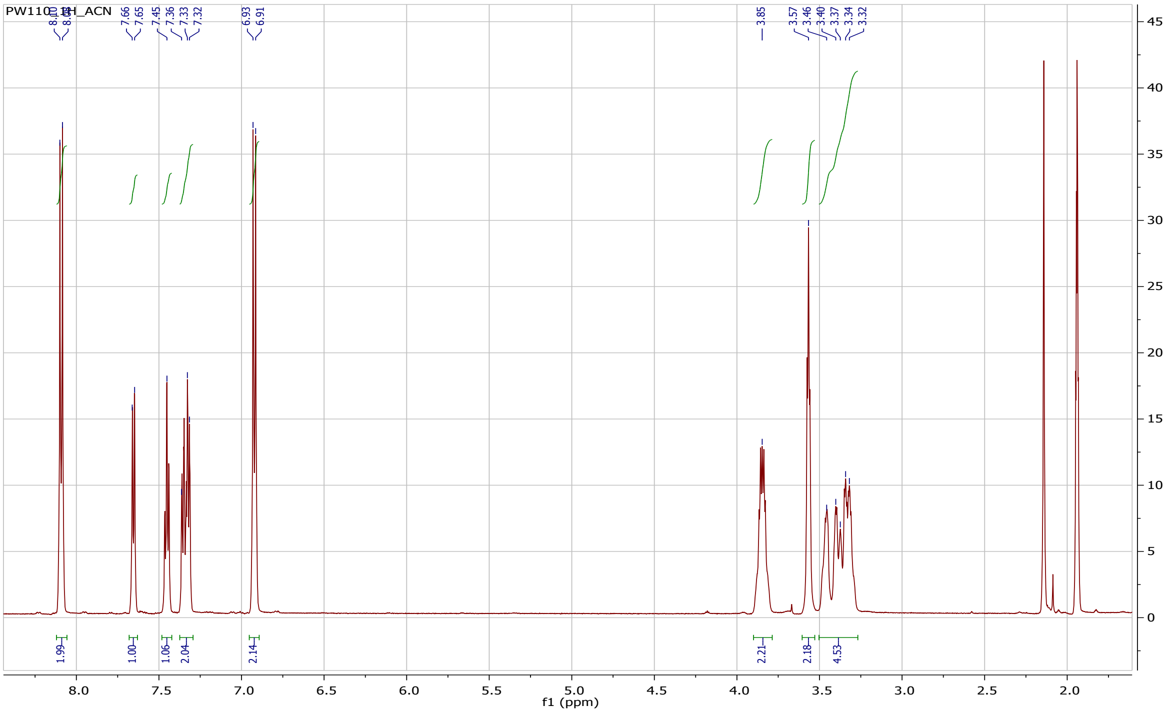

Figure S55. ^{1}H NMR spectrum of compound **6j** measured in acetonitrile-d_3.

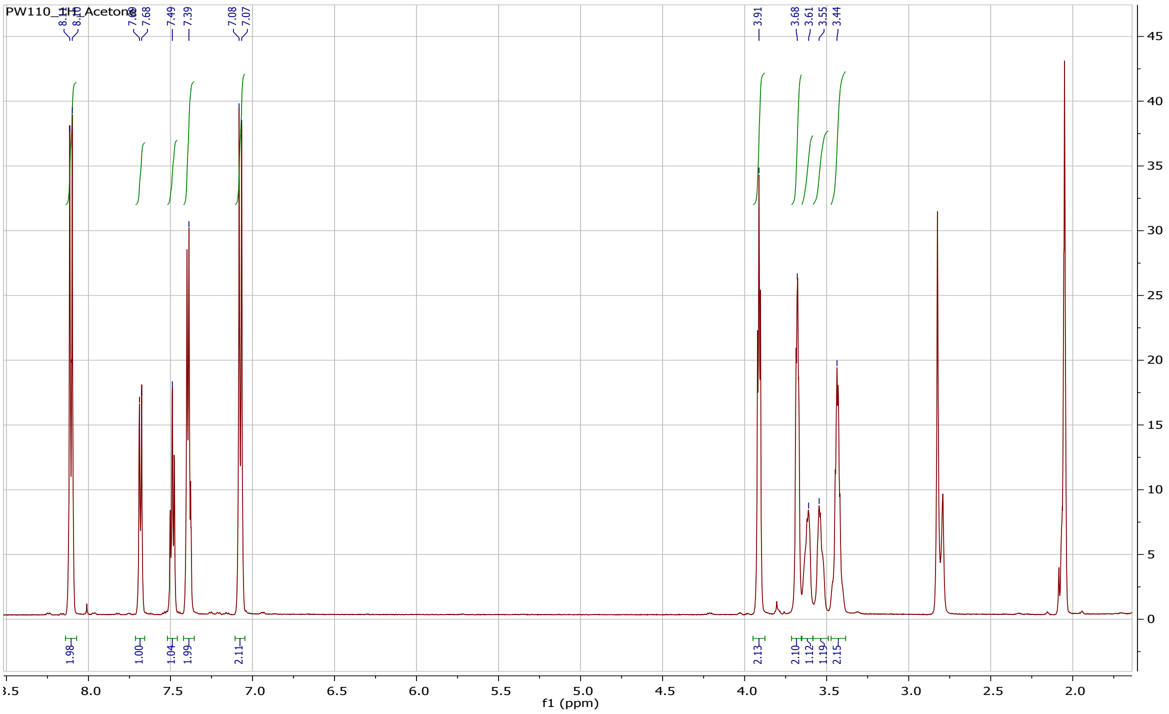

Figure S56. ^{1}H NMR spectrum of compound **6j** measured in acetone-d_6.

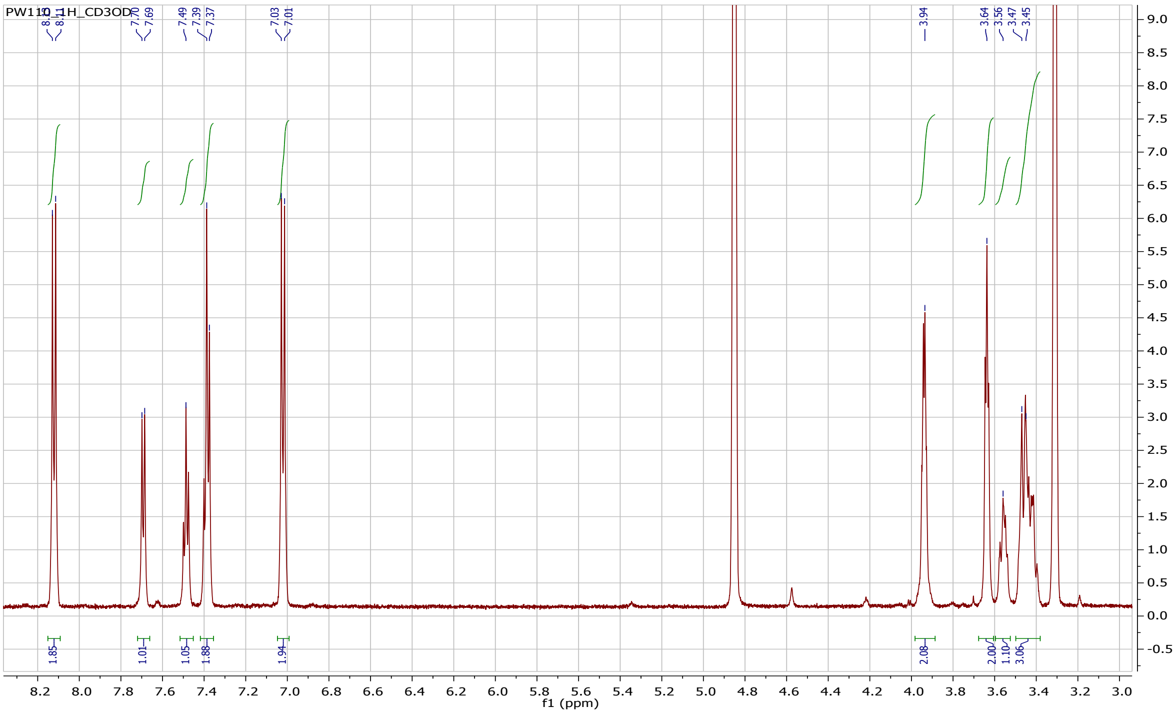

Figure S57. ^{1}H NMR spectrum of compound **6j** measured in methanol-d$_4$.

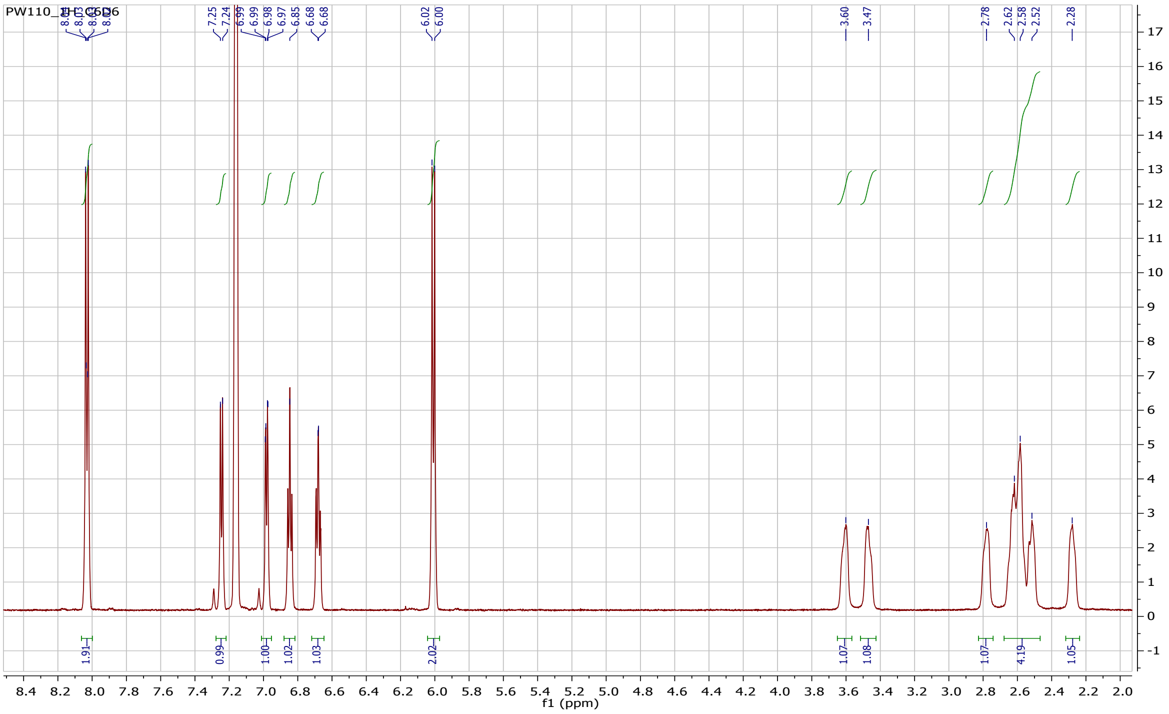

Figure S58. ^{1}H NMR spectrum of compound **6j** measured in benzene-d$_6$.

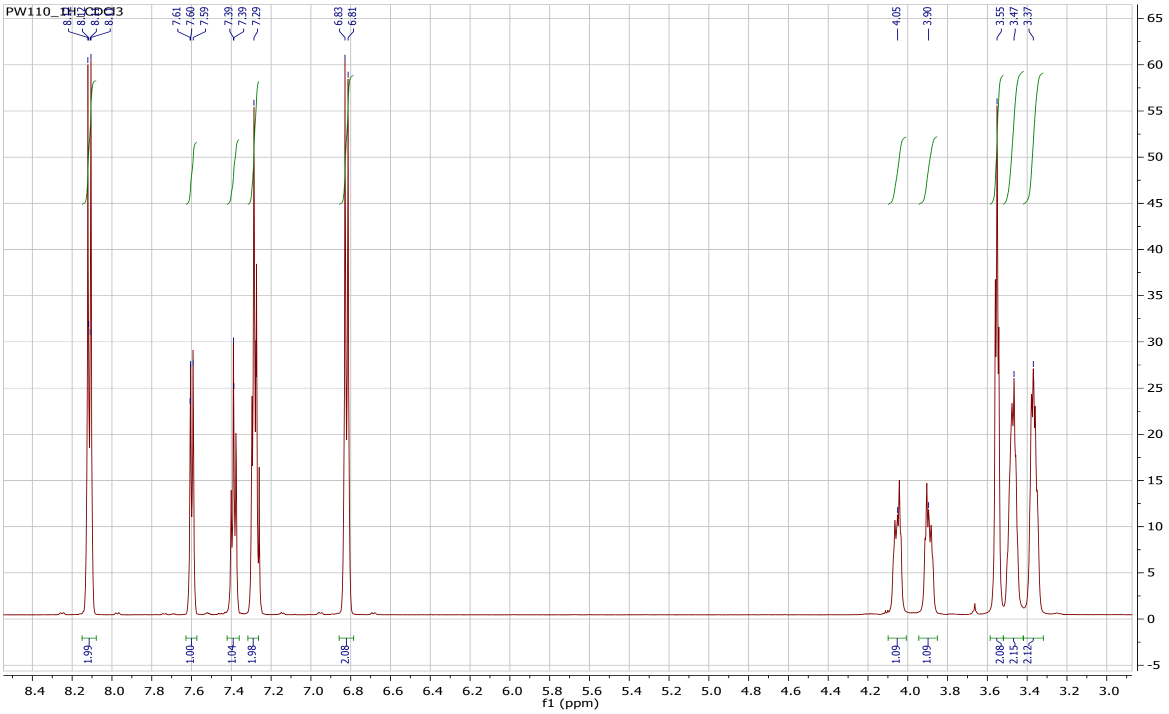

Figure S59. ^{1}H NMR spectrum of compound **6j** measured in $CDCl_3$.

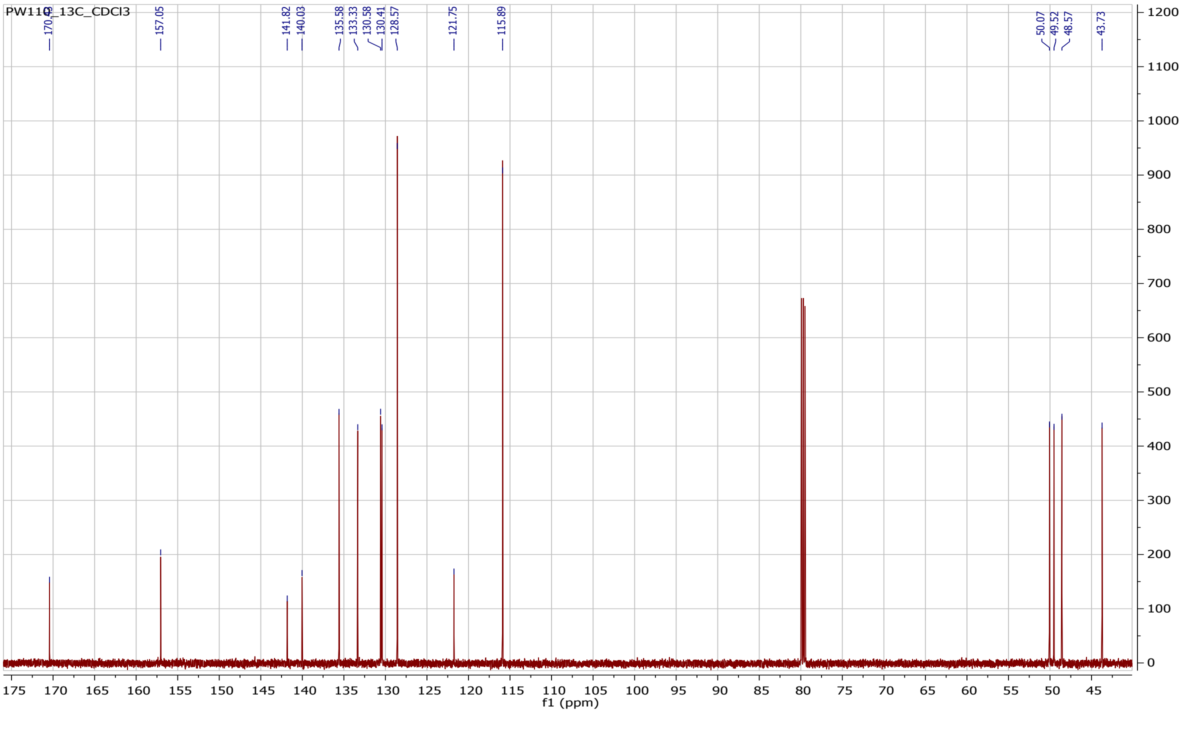

Figure S60. ^{13}C NMR spectrum of compound **6j** measured in $CDCl_3$.

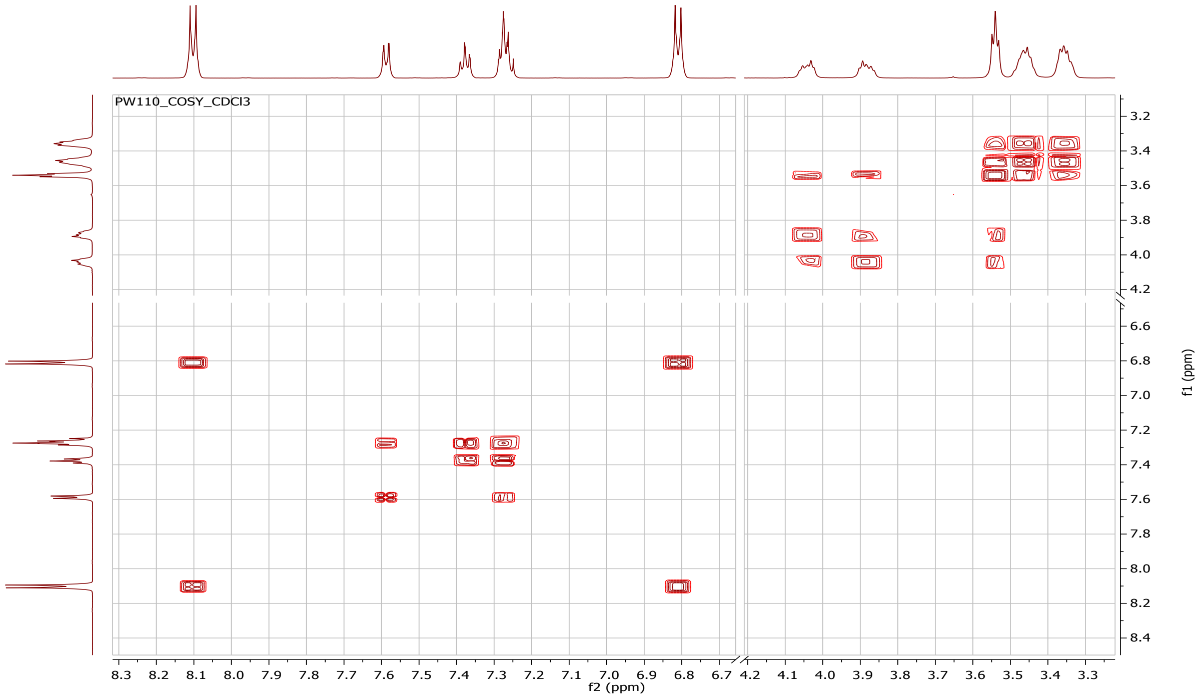

Figure S61. H-H-COSY spectrum of compound **6j** measured in $CDCl_3$.

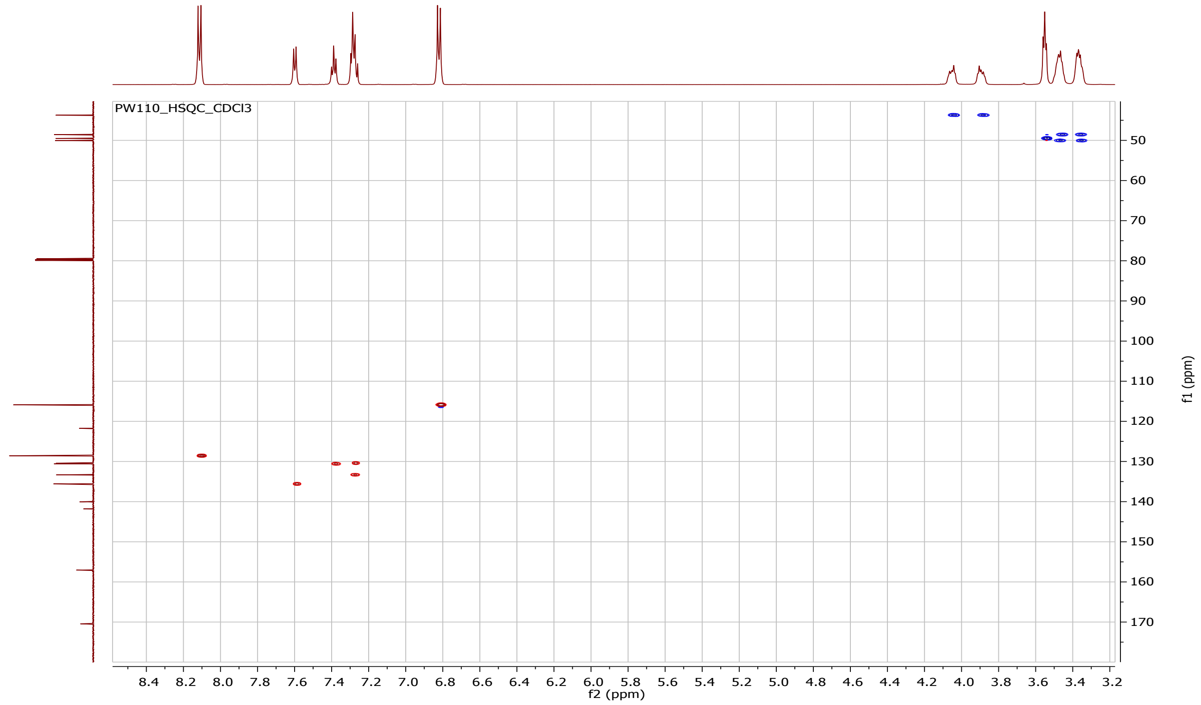

Figure S62. HSQC spectrum of compound **6j** measured in $CDCl_3$.

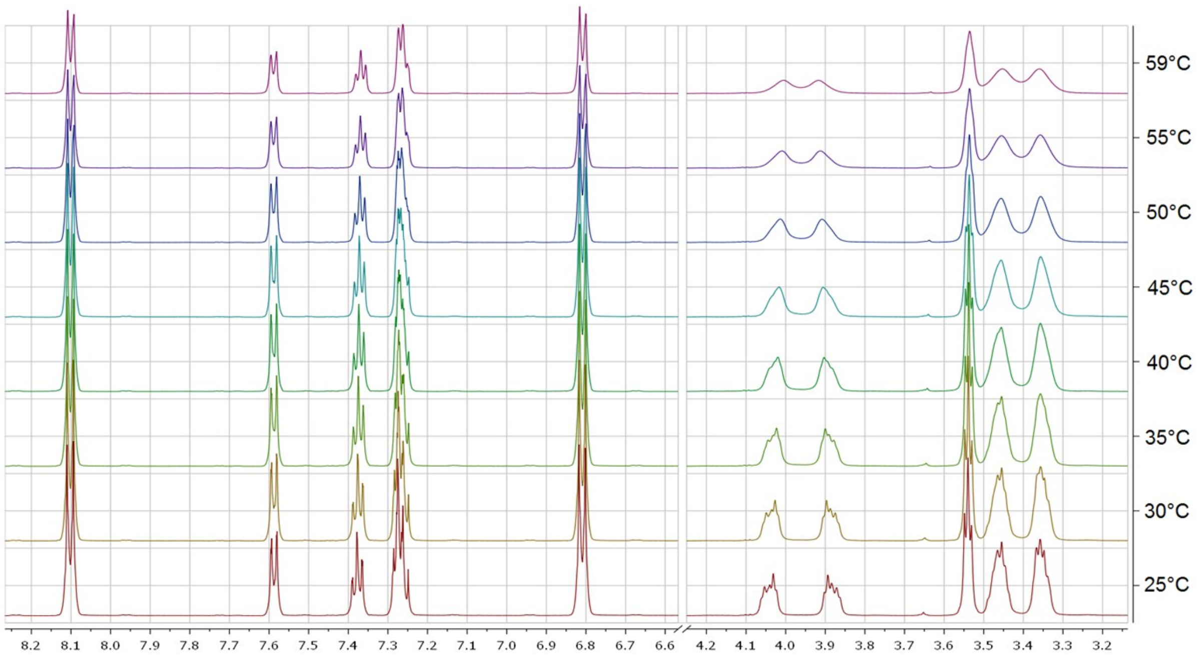

Figure S63. Temperature-depended ^{1}H NMR spectrum of compound **6j** measured in $CDCl_3$.

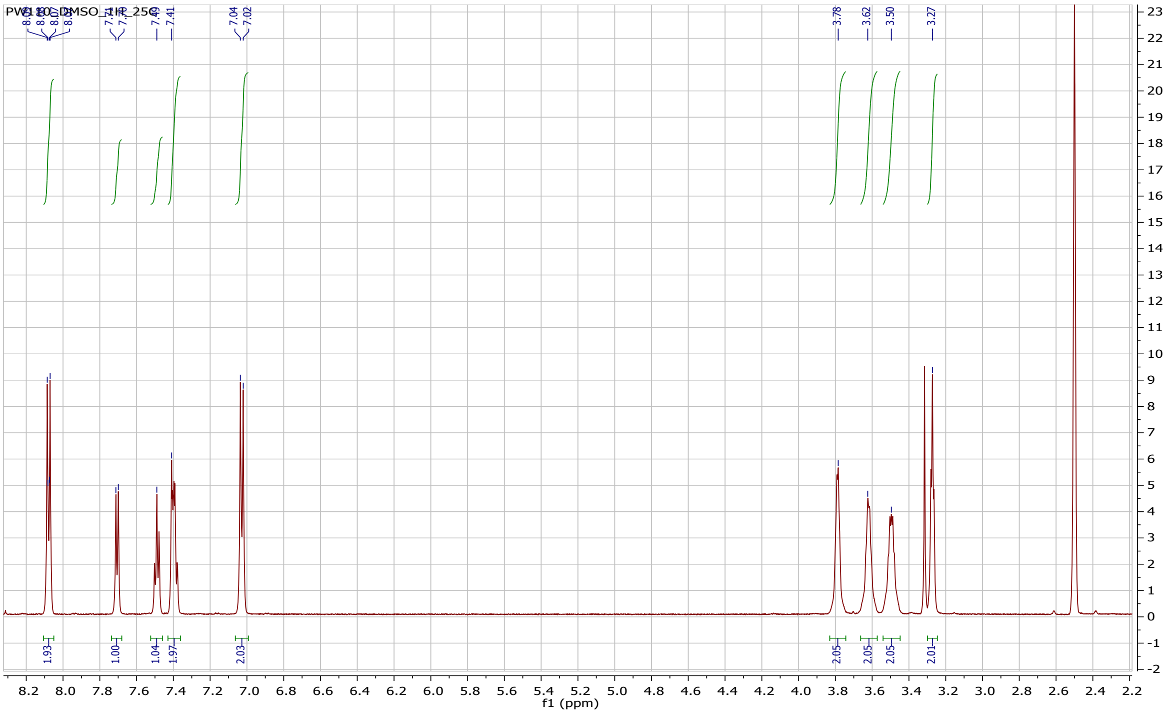

Figure S64. ^{1}H NMR spectrum of compound **6j** measured in DMSO-d_6.

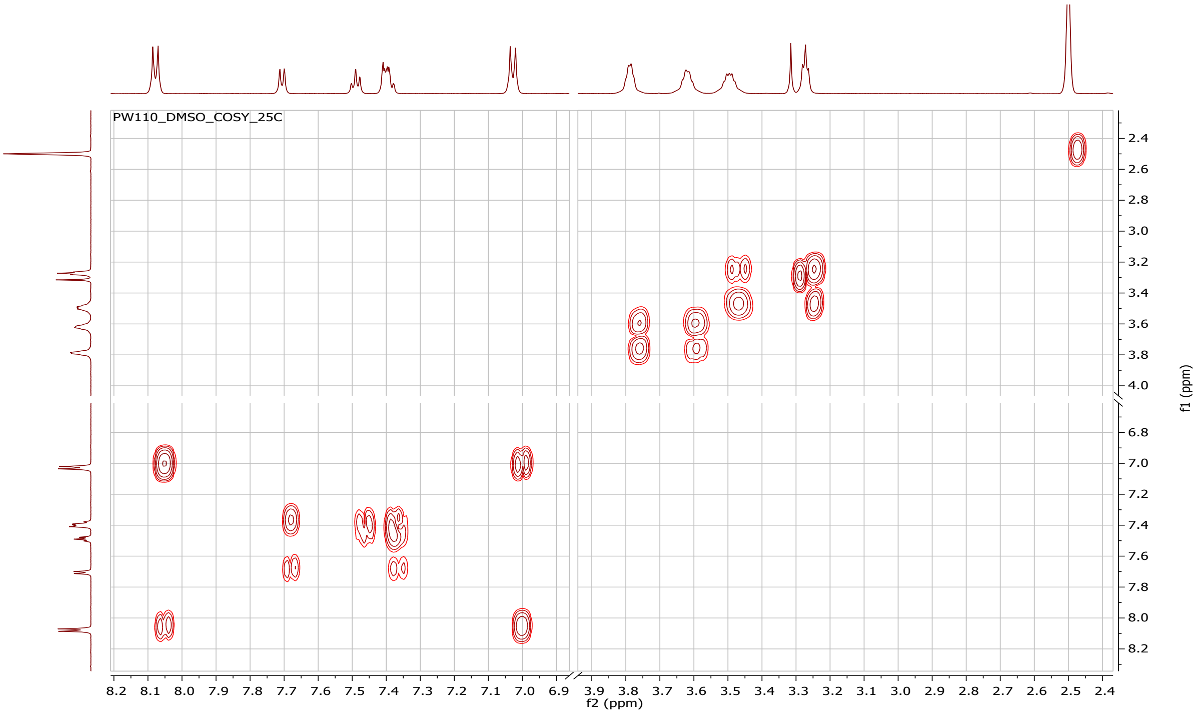

Figure S65. H-H-COSY spectrum of compound **6j** measured in DMSO-d$_6$.

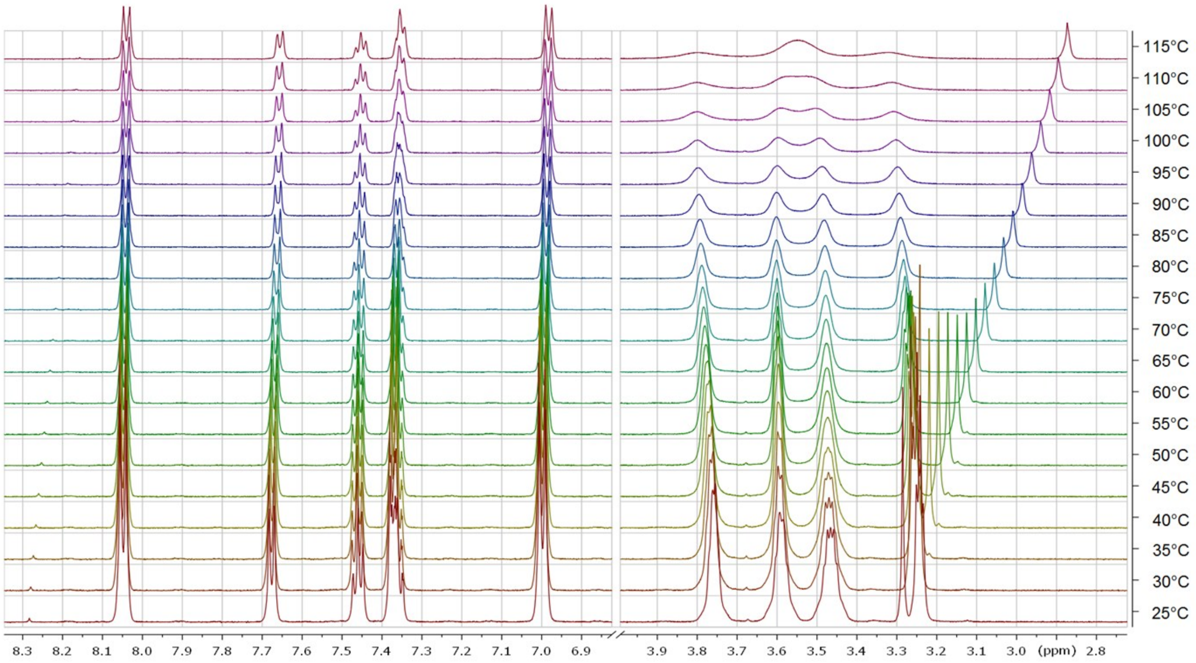

Figure S66. Temperature-depended ^{1}H NMR spectrum of compound **6j** measured in DMSO-d$_6$.

N-(2-Nitrobenzoyl)-*N*′-(4-nitrophenyl)piperazine (**6k**)

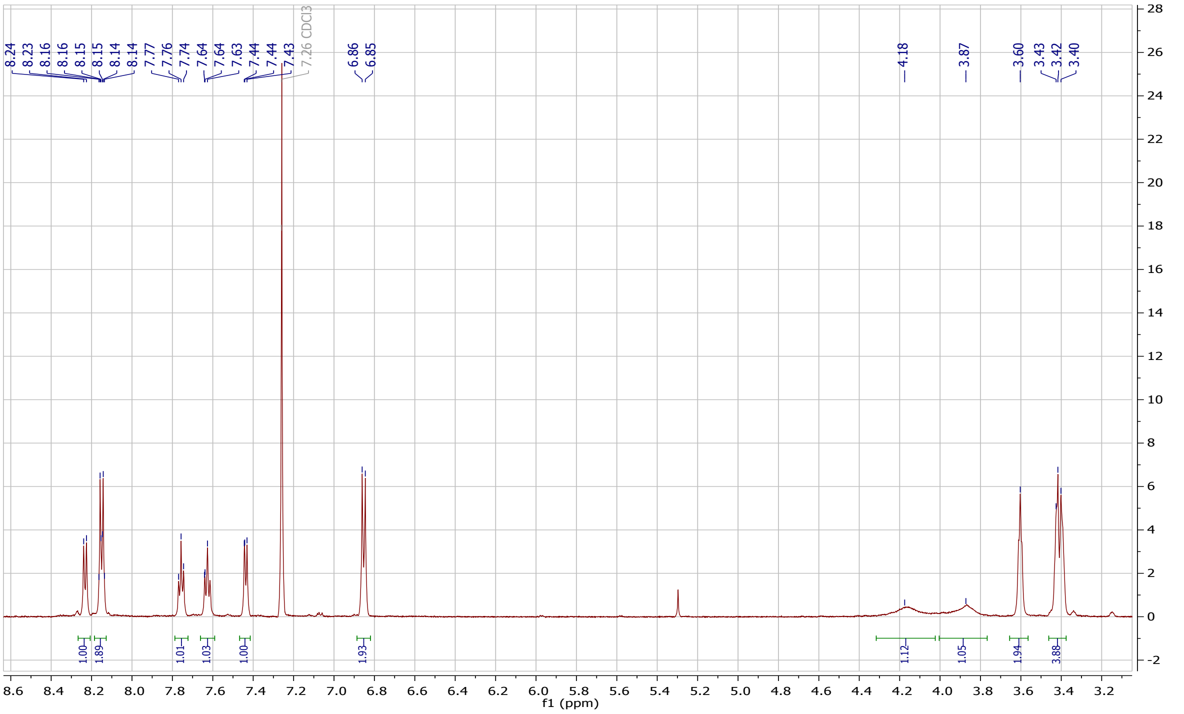

Figure S67. ^{1}H NMR spectrum of compound **6k** measured in $CDCl_3$.

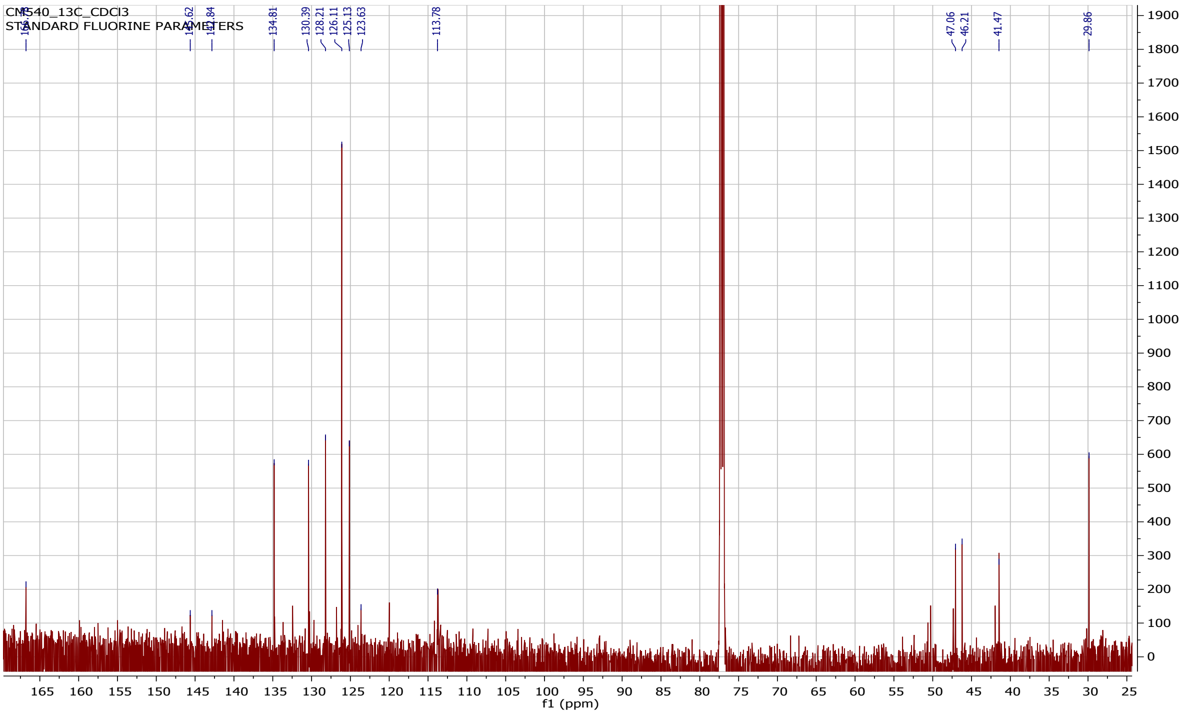

Figure S68. ^{1}H NMR spectrum of compound **6k** measured in $CDCl_3$.

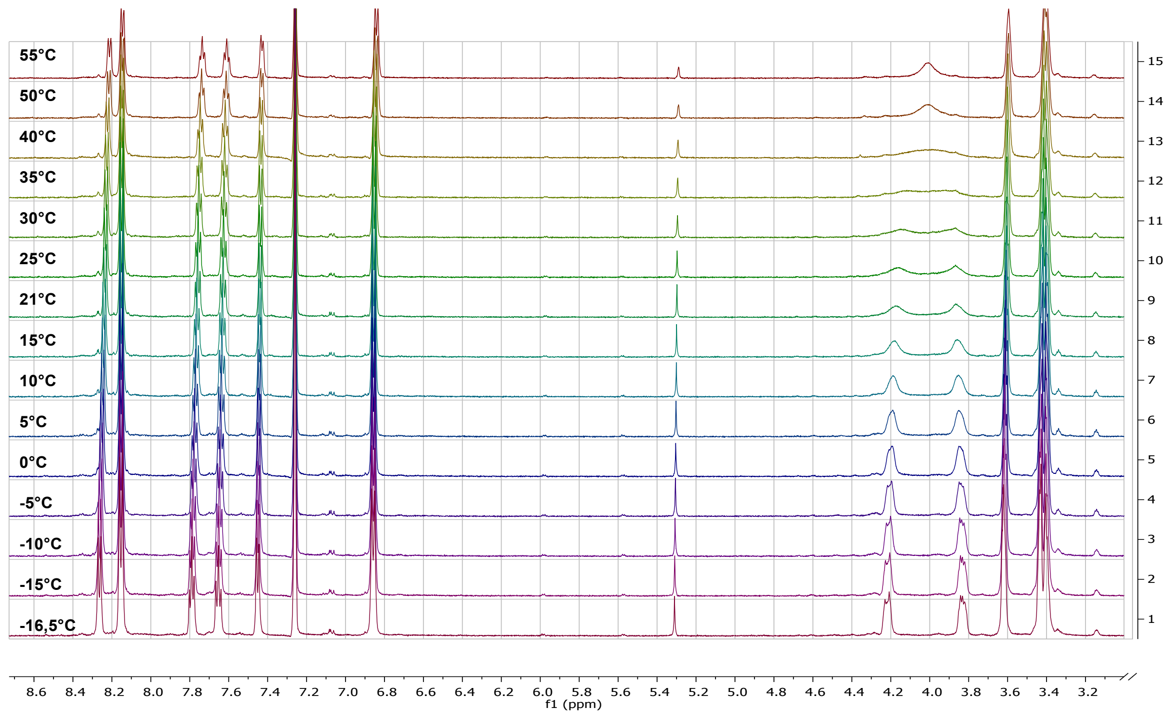

Figure S69. Temperature-depended ^{1}H NMR spectrum of compound **6k** measured in $CDCl_3$.

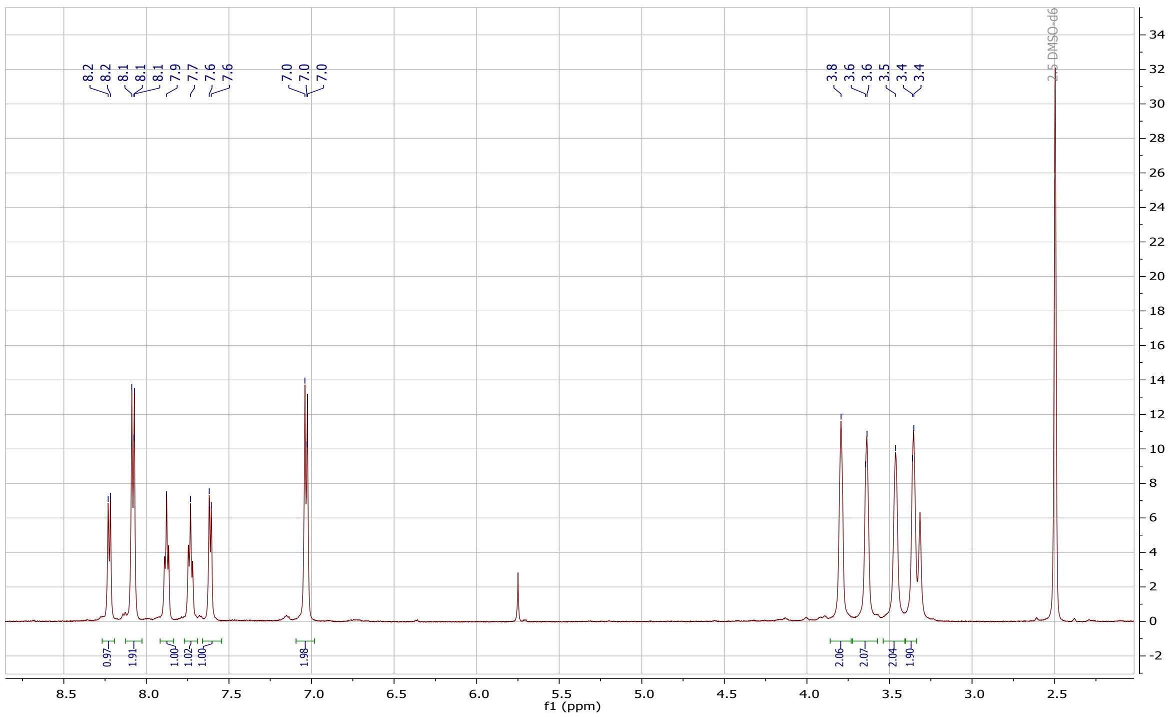

Figure S70. ^{1}H NMR spectrum of compound **6j** measured in DMSO-d_6.

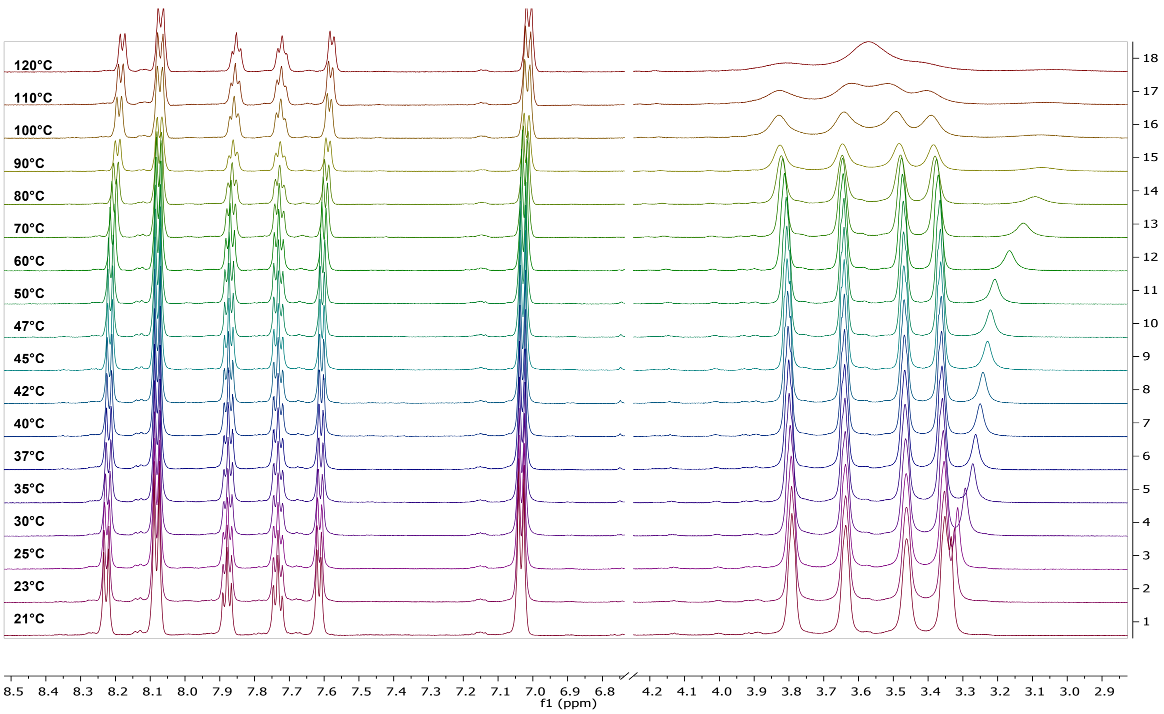

Figure S71. Temperature-depended ^{1}H NMR spectrum of compound **6k** measured in DMSO-d_6.

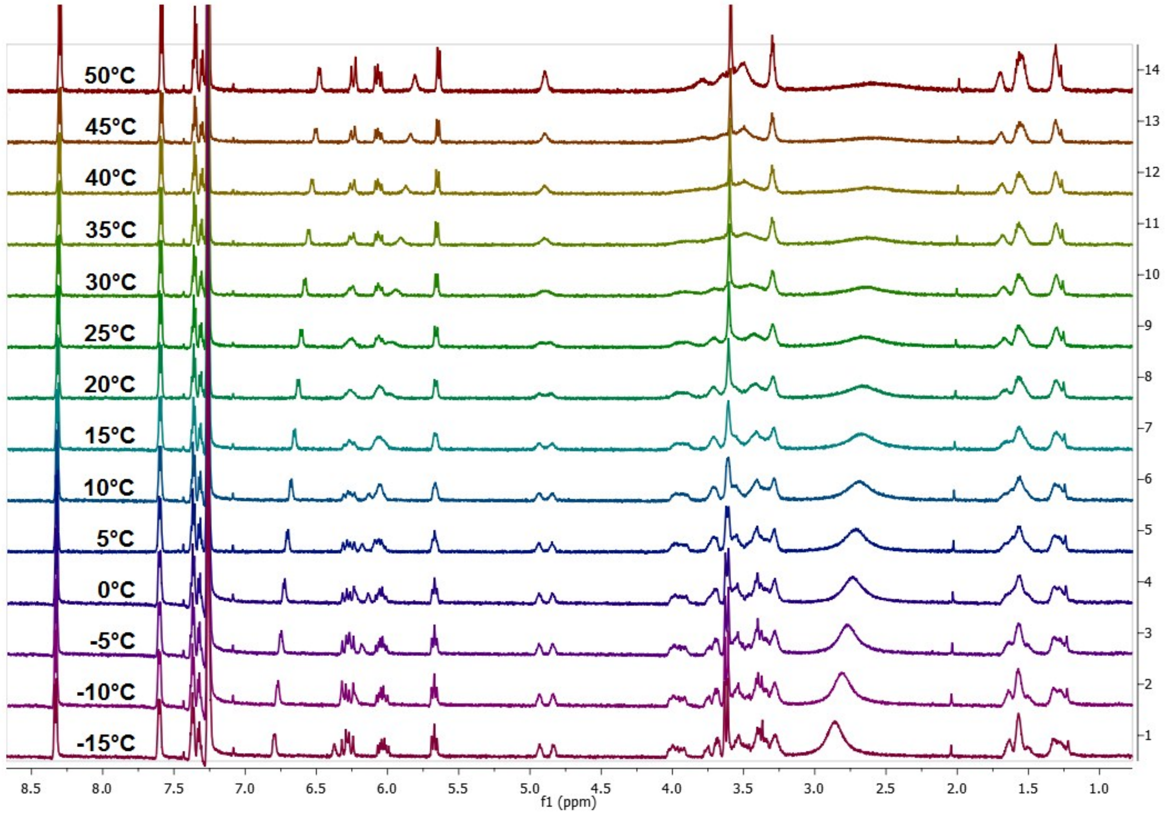

Figure S72. Temperature-dependent ^1H NMR spectra of fluorine compound **7** measured in $CDCl_3$.

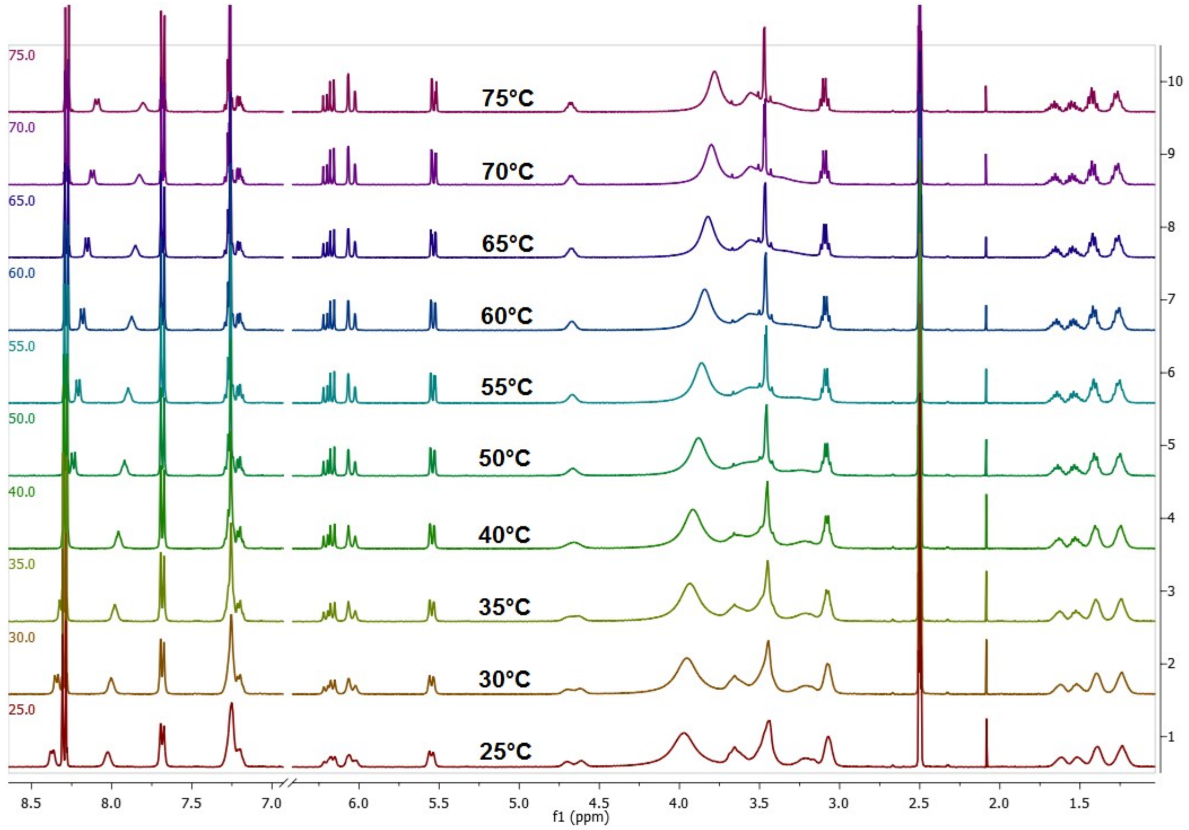

Figure S73. Temperature-dependent ^1H NMR spectra of fluorine compound **7** measured in DMSO-d_6.

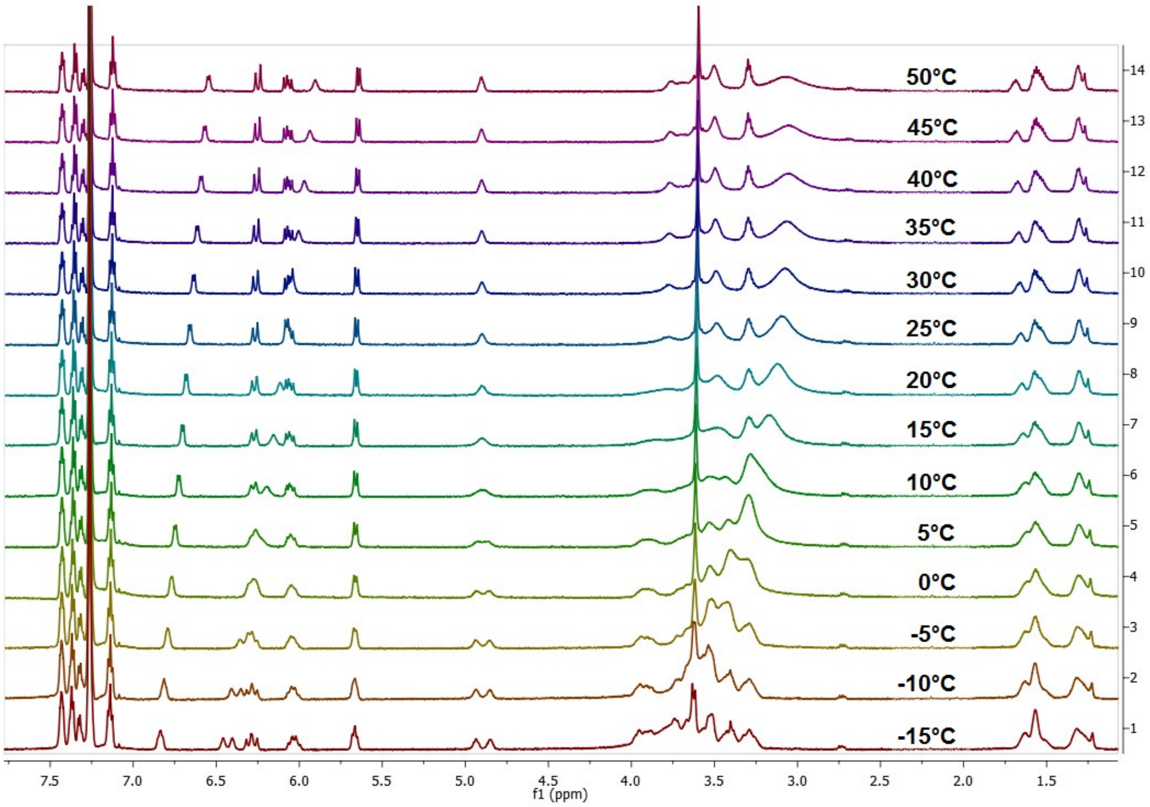

Figure S74. Temperature-dependent ^{1}H NMR spectra of fluorine compound **8** measured in $CDCl_3$.

Series 3: σ_p in DMSO-d_6

Amine $\Delta G^{\ddagger} = 4.86\sigma_p + 61.79$ $n = 6; s = 0.516; r = 0.956; R^2 = 0.914$

Amide $\Delta G^{\ddagger} = 5.90\sigma_p + 61.45$ $n = 7; s = 1.062; r = 0.873; R^2 = 0.762$

Series 3: σ_p^- in DMSO-d_6

Amine $\Delta G^{\ddagger} = 3.16\sigma_p^- + 61.96$ $n = 6; s = 0.460; r = 0.965; R^2 = 0.932$

Amide $\Delta G^{\ddagger} = 3.55\sigma_p^- + 61.79$ $n = 7; s = 1.216; r = 0.830; R^2 = 0.688$

Series 3: σ_{p+} in DMSO-d_6

Amine $\Delta G^{\ddagger} = 4.14\sigma_p^+ + 62.25$ $n = 6; s = 0.576; r = 0.945; R^2 = 0.893$

Amide $\Delta G^{\ddagger} = 5.07\sigma_p^+ + 62.02$ $n = 7; s = 1.037; r = 0.879; R^2 = 0.773$

Series 3: σ_p in $CDCl_3$

Amine $\Delta G^{\ddagger} = 4.78\sigma_p + 60.72$ $n = 9; s = 0.983; r = 0.851; R^2 = 0.724$

Amide $\Delta G^{\ddagger} = 4.73\sigma_p + 60.52$ $n = 8; s = 0.998; r = 0.750; R^2 = 0.562$

Series 3: σ_p^- in $CDCl_3$

Amine $\Delta G^{\ddagger} = 3.27\sigma_p^- + 60.78$ $n = 9; s = 1.009; r = 0.842; R^2 = 0.709$

Amide $\Delta G^{\ddagger} = 4.48\sigma_p^- + 60.54$ $n = 8; s = 1.019; r = 0.737; R^2 = 0.0544$

Series 3: σ_p^+ in $CDCl_3$

Amine $\Delta G^{\ddagger} = 3.76\sigma_p^+ + 61.31$ $n = 9; s = 0.679; r = 0.932; R^2 = 0.868$

Amide $\Delta G^{\ddagger} = 3.46\sigma_p^+ + 61.06$ $n = 8; s = 0.695; r = 0.888; R^2 = 0.788$

Series 6: σ_p in $CDCl_3$

Amine $\Delta G^{\ddagger} = 4.24\sigma_p + 58.74$ $n = 7; s = 0.888; r = 0.882; R^2 = 0.779$

Amide $\Delta G^{\ddagger} = 4.67\sigma_p + 58.90$ $n = 7; s = 0.982; r = 0.882; R^2 = 0.777$

Series 6: σ_p^- in $CDCl_3$

Amine $\Delta G^{\ddagger} = 2.81\sigma_p^- + 58.77$ $n = 7; s = 0.991; r = 0.851; R^2 = 0.724$

Amide $\Delta G^{\ddagger} = 3.18\sigma_p^- + 58.90$ $n = 7; s = 1.018; r = 0.872; R^2 = 0.761$

Series 6: σ_p^+ in $CDCl_3$

Amine $\Delta G^{\ddagger} = 3.32\sigma_p^+ + 59.28$ $n = 7; s = 0.524; r = 0.961; R^2 = 0.923$

Amide $\Delta G^{\ddagger} = 3.59\sigma_p^+ + 59.50$ $n = 7; s = 0.705; r = 0.941; R^2 = 0.885$

The following substituent parameters were used for correlation analyses.[1]

σ_m: 0.39 (3-Br)

σ_p: -0.17 (CH_3), -0.27 (OCH_3), 0.06 (F), 0.23 (Cl), 0.23 (Br), 0.18 (I) and 0.78 (NO_2)

σ_p^-: -0.17 (CH_3), -0.26 (OCH_3), -0.03 (F), 0.19 (Cl), 0.25 (Br), 0.27 (I) and 1.27 (NO_2)

σ_p^+: -0.31 (CH_3), -0.78 (OCH_3), -0.07 (F), 0.11 (Cl), 0.15 (Br), 0.14 (I) and 0.78 (NO_2)

1 C. Hansch and A. Leo, *Exploring QSAR Fundamentals and Applications in Chemistry and Biology*; American Chemical Society, Washington, 1995; p 1-24 ff.

Table S2. Crystal data and structure refinement for compounds **4d**, **6d**, **6f**, and **6i**.

Compound	**4d**	**6d**	**6f**	**6i**
Formula	$C_{18}H_{16}F_2N_2O_2$	$C_{17}H_{16}FN_3O_3$	$C_{17}H_{16}BrN_3O_3$	$C_{17}H_{16}BrN_3O_3$
Formula weight (g·mol^{-1})	330.33	329.33	390.24	390.24
Temperature (K)	123	123	296	123
Crystal system	monoclinic	monoclinic	monoclinic	monoclinic
Space group	$P2_1/n$	$P2_1/n$	$C2/c$	$P2_1/c$
Unit cell dimensions:				
a (Å)	6.5750(4)	9.7759(6)	17.379(2)	7.842(3)
b (Å)	10.4009(7)	14.3497(9)	11.788(2)	25.300(9)
c (Å)	11.2622(7)	10.452(2)	17.806(2)	8.024(1)
β (°)	101.313(2)	101.452(2)	116.727(7)	94.67(1)
Volume (Å^3), Z	755.21(8), 2	1497.2(2), 4	3257.8(7)	1586.7(9)
Data/restraints/param.	6803/0/109	7230/0/218	6516/0/217	3910/0/335
Measured reflections	44151	74137	33067	35509
ϑ_{max} (°)	23.4	36.4	31.4	27.2
GoF on F^2	1.07	1.08	1.00	1.08
R1 [$I > 2\sigma(I)$]	0.049	0.048	0.039	0.060
$wR2$ (all data)	0.165	0.129	0.096	0.107
Larg. diff. peak/hole (e·Å^3)	0.63/-0.37	0.51/-0.28	0.55/-0.56	0.82/-9,65
